# Teleost Fish Mount Complex Clonal IgM and IgT Responses in Spleen upon Systemic Viral Infection

**DOI:** 10.1371/journal.ppat.1003098

**Published:** 2013-01-10

**Authors:** Rosario Castro, Luc Jouneau, Hang-Phuong Pham, Olivier Bouchez, Véronique Giudicelli, Marie-Paule Lefranc, Edwige Quillet, Abdenour Benmansour, Frédéric Cazals, Adrien Six, Simon Fillatreau, Oriol Sunyer, Pierre Boudinot

**Affiliations:** 1 Virologie et Immunologie Moléculaires, INRA, Jouy-en-Josas, France; 2 UPMC Univ Paris 06, UMR 7211, “Integrative Immunology” Team, Paris, France; CNRS, UMR 7211, “Immunology, Immunopathology, Immunotherapy,” Paris, France; 3 UMR INRA 0444 Laboratoire de Génétique Cellulaire, GeT-PlaGe Core Facility, Castanet Tolosan, France; 4 IMGT, the International ImMunoGeneTics Information System, Laboratoire d'ImmunoGénétique Moléculaire LIGM, IGH, UPR CNRS 1142 and Université Montpellier 2, Montpellier, France; 5 Génétique Animale et Biologie Intégrative, INRA, Jouy-en-Josas, France; 6 INRIA Sophia-Antipolis - Méditerranée, Algorithms-Biology-Structure, Sophia-Antipolis, France; 7 Deutsches RheumaForschungszentrum, a Leibniz Institute, Berlin, Germany; 8 Department of Pathobiology, School of Veterinary Medicine, University of Pennsylvania, Philadelphia, Pennsylvania, United States of America; University of California San Francisco, United States of America

## Abstract

Upon infection, B-lymphocytes expressing antibodies specific for the intruding pathogen develop clonal responses triggered by pathogen recognition via the B-cell receptor. The constant region of antibodies produced by such responding clones dictates their functional properties. In teleost fish, the clonal structure of B-cell responses and the respective contribution of the three isotypes IgM, IgD and IgT remain unknown. The expression of IgM and IgT are mutually exclusive, leading to the existence of two B-cell subsets expressing either both IgM and IgD or only IgT. Here, we undertook a comprehensive analysis of the variable heavy chain (VH) domain repertoires of the IgM, IgD and IgT in spleen of homozygous isogenic rainbow trout (*Onchorhynchus mykiss*) before, and after challenge with a rhabdovirus, the Viral Hemorrhagic Septicemia Virus (VHSV), using CDR3-length spectratyping and pyrosequencing of immunoglobulin (Ig) transcripts. In healthy fish, we observed distinct repertoires for IgM, IgD and IgT, respectively, with a few amplified μ and τ junctions, suggesting the presence of IgM- and IgT-secreting cells in the spleen. In infected animals, we detected complex and highly diverse IgM responses involving all VH subgroups, and dominated by a few large public and private clones. A lower number of robust clonal responses involving only a few VH were detected for the mucosal IgT, indicating that both IgM^+^ and IgT^+^ spleen B cells responded to systemic infection but at different degrees. In contrast, the IgD response to the infection was faint. Although fish IgD and IgT present different structural features and evolutionary origin compared to mammalian IgD and IgA, respectively, their implication in the B-cell response evokes these mouse and human counterparts. Thus, it appears that the general properties of antibody responses were already in place in common ancestors of fish and mammals, and were globally conserved during evolution with possible functional convergences.

## Author Summary

In humans and mice, a broad and robust antibody response is the most effective system of defense against pathogens. While all vertebrates but the most ancient use an antibody system, it remains unknown if the clonal selection rules are conserved across vertebrates. Using high-throughput sequencing and antibody repertoire analysis, we show in teleost fish that virus infection induces a typical antibody response with clonal expansions. In spleen, the antibody response mainly consists in IgM, which is found across vertebrates, with a significant contribution of the fish-specific mucosal antibody IgT. IgD does not contribute as already observed in mammals. Although antibody molecules can be of different nature and structure across species, the general features of adaptive immunity were already in place in common ancestors of fish and mammals, and globally conserved during evolution while the anatomy and physiology of the immune system diverged.

## Introduction

The immune system of mammals is characterized by the presence of B and T lymphocytes, each carrying a single receptor for antigen generated through somatic rearrangements of V-(D)-J genes. Upon infection specific B and T lymphocytes develop protective responses against the intruding pathogen, and after disease resolution they maintain an increased resistance against the eliciting agent through persistence of memory cells. The two properties of specificity and memory proceed from the fundamental fact that a few T and B cell clones expressing receptors specific for the eliciting antigen expand and carry out the adaptive response, as formulated in the clonal theory of immunity [1], [2].

The origin and development of this remarkable defense system during the evolution of vertebrates remain poorly understood. The primordial adaptive immune system of extinct vertebrates is no longer accessible, but it can be approached indirectly through comparative analyses of B and T cell responses in distant contemporary species such as teleost fishes and mammals, which diverged more than 350 million years ago. Indeed, common fundamental features like typical B and T lymphocytes were already present in their last common ancestor [3]. However, it is important to note that the organization of lymphoid tissues, where lymphocytes develop, encounter with antigen, and get activated, is profoundly different between fish and mammals [4]. While in mammals B lymphopoiesis occurs in bone marrow, fish lack this tissue and their B cells differentiate in the anterior kidney or pronephros [5], [6]. Fish also lack lymph nodes, so that B and T cell responses occur mainly in spleen and mucosal territories. Due to these anatomical differences, it might be expected that fish and mammal T and B cell responses show distinct functional properties.

We previously showed that T cell diversity was extensive in central lymphoid organs of fish, and that antiviral T cell response was comparable to that of mammals, with typical public and private components [7]. However, no T cell response was observed at 10°C in trout, and at 16°C the kinetics of the response was slower compared to mammals. The TCRβ (TRB) repertoire of the gut was highly diverse even in adult fish, in contrast to the one of mouse and human [8]. Taken together, these studies indicate that some, but not all, features of T cell immunity are similar between fish and mammals.

Regarding B cell diversity and responses, the lack of germinal centers and lymph nodes could have more dramatic consequences than for T cells since B cells require a proper microenvironment for differentiation and maturation [9], [10]. While B cell responses have been observed after immunization with antigens in many fish species, allowing efficient vaccination against viral diseases, some species like Atlantic cod (*Gadus morhua*) do not show specific antibodies (Ab) responses (probably due to the lack of MHC class II molecules) despite high amount of serum “natural” Abs [11], [12]. In rainbow trout (*Oncorhynchus mykiss*), different types of Ab secreting cells have been distinguished, as in mouse and human [13]: plasmablasts, which produce low amount of Ab, replicate, and express low level of B cell receptor (BCR), and plasma cells (long- or short-lived), which produce high level of Ab, do not replicate nor express BCR [5], [6], [14]. Importantly, while B cells encounter their specific target in spleen or kidney, and differentiate into Ab secreting cells in these tissues, mature plasma cells migrate and persist in the anterior kidney [15], as observed in mouse or in human for the bone marrow [16]. However, fish B cells lack efficient affinity maturation, as observed also in amphibians [9], [10].

Teleost fish have three heavy chain isotypes: μ and δ that correspond to the IgM and IgD classes found in all vertebrates with jaws (gnathostomata), and τ that encodes the IgT class, which is specific to fish [17]. IgM and IgT are never co-expressed by the same B cell, which identifies two distinct lineages of B cells in fish [18]. In fact, the configuration of the IG locus ensures the exclusive expression of either IgM/IgD or IgT; isotypic commutation and switch recombination do not occur in fish [19]. It is currently thought that IgM is primarily a systemic immunoglobulin. The function of fish IgD is still elusive, yet it may have a role in innate immunity. In the channel catfish (*Ictalurus punctatus*), secreted IgD lacking the antigen-specific V domain could bind to basophils to induce proinflammatory cytokines [20]. IgT is specialized in gut mucosal immunity in rainbow trout: a gut protozoan parasite elicited a local IgT response, while the IgM response was restricted to the serum [21]. Additionally, intestinal bacteria were coated by IgT, suggesting that this IG class might play a role in the interactions between the host intestinal mucosa and microflora [21].

While the diversity of antibody response was initially considered lower in fish and amphibians compared to mammals, the recent discoveries of fish genomics revealed that the potential combinatorial Ab repertoire is probably bigger in many fish species than in humans and mice [22]. The first complete description of the V domain repertoire expressed by a vertebrate organism was recently achieved from whole healthy zebrafish [23], with the usage of 454 GS FLX high-throughput pyrosequencing. Focused on the naive IgM repertoire, this study showed that most of the possible combinations of IGH V, D and J genes were expressed in all individuals with a similar frequency distribution. A network analysis of these data found a common architecture of sequence diversity in different fish, beyond the individual differences [24], [25].

The implication of the different isotypes in the defense against pathogens is still largely unknown in fish, as well as the clonal complexity of their respective responses. Here, we have performed a comprehensive study of the clonal complexity of B cell repertoire of healthy fish as well as after systemic infection, for all expressed isotypes. To investigate if the clonal nature of the B cell response and the various isotype contributions were conserved across species, we characterized not only the IgM, but also IgT and IgD responses of the rainbow trout (*Oncorhynchus mykiss*) against a rhabdovirus, the Viral Hemorrhagic Septicemia Virus (VHSV). We considered the available repertoire expressed by the cells that can respond to the antigens at a given moment in a given tissue of the individual. In this line, the available repertoire is not a mere list but also refers to the frequency of the different specificities. As in a recent report by Ademokun *et al.* [26], we used a combination of CDR3 spectratyping and 454 GS FLX pyrosequencing. Our data reveal a highly diversified available VH domain repertoire, and demonstrate that fish Ab response presents features typical of adaptive responses previously described for mammals, as we previously observed for T cells. Remarkably, we observed a broad polyclonal response implicating a large number of VH subgroups.

## Results

### B cells from the spleen of healthy fish express highly diverse IgM, IgD and IgT repertoires

**Figure 1:**
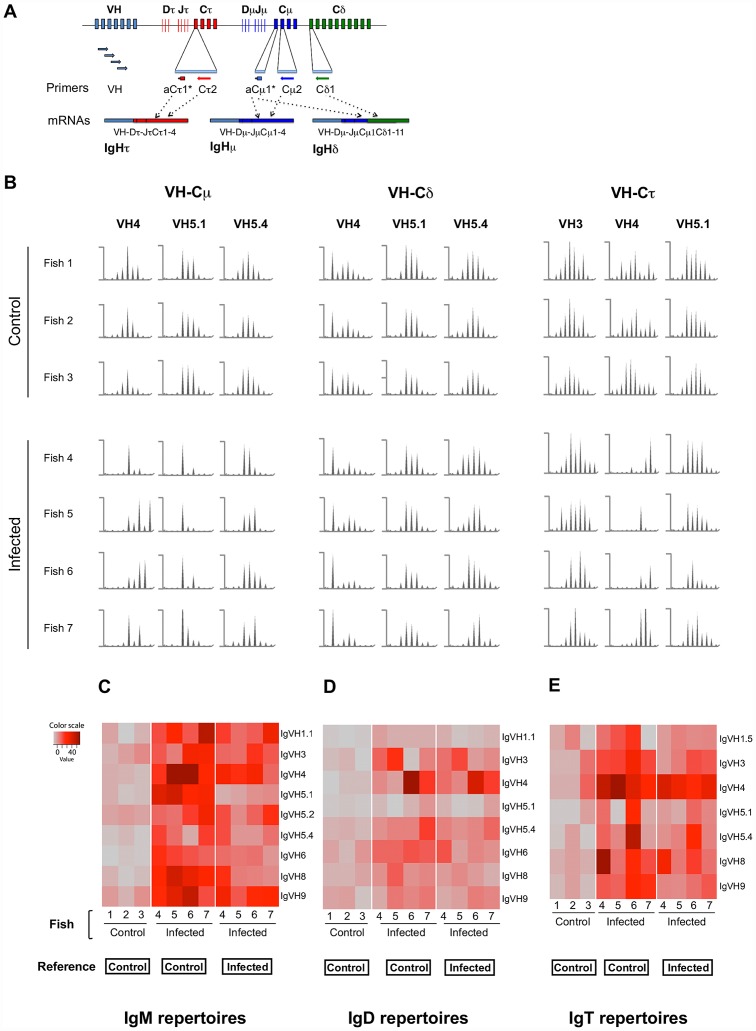
Spectratyping analysis of the spleen B cell response to VHSV. (A) Schematic representation of rainbow trout immunoglobulin heavy (IGH) locus, Immunoscope primers and typical IGH μ, δ and τ transcripts. * denotes the fluorescent primers used in run-offs. (B) CDR3 length distribution profiles of μ, δ and τ transcripts for selected VHC combinations in the spleen of naïve and infected animals. Y-axis: fluorescence arbitrary units, x-axis: length of run-off products; for the sake of clarity, Max and Min values for each spectratyping profile are given in Figure S1C. (C–E) Perturbation scores calculated to reference profile representing the average of the VHCμ, VHCδ or VHCτ distributions, for the IgM, IgD or IgT, in control or infected animals. Scores are represented on grey to red-scale, red color indicating a strong difference of a given profile to the reference.

As a start, we characterized the expressed Ab repertoire from three healthy homozygous isogenic fish. To this end, we used a spectratyping approach that determines the profile of CDR3 length distribution for each VHC combination, based on PCR reactions between Ig constant (C) and variable (V) regions [27]. First, heavy chain rearranged transcripts (IGH V-D-J-C) were amplified using a set isotype-specific primers for IgM, IgD and IgT, respectively, and a set of IGHV subgroup-specific primers that can amplify all members of the 11 known IGHV groups (Figure 1A and Figure S1A) [28]. Of these 11 VH groups, we found that seven were used by all three isotypes (Figure S1B), suggesting that a large fraction of the potential repertoire was expressed in peripheral B cells from healthy fish. To assess the diversity of these VH (V-D-J) rearrangements, each VHC PCR product was then subjected to a run-off reaction using a labeled internal C-specific primer. The resulting products, which reflect the variable numbers of nucleotides present in the CDR3 region, were analyzed on a sequencing apparatus to determine their size distribution. These analyses produced bell-shaped profiles for all VHC primer combinations in healthy fish (Figure 1B), which is a typical feature of immune repertoire expressed by naïve lymphocytes in mammals [27]. The profiles consisted of 4–10 peaks for VHCμ (IgM) and VHCδ (IgD), and 5–13 peaks for VHCτ (IgT), separated by intervals of three nucleotides as expected from in-frame transcripts (Figure 1B; profiles for all expressed VH subgroups are shown in Figure S2A). IgT junctions were therefore longer than those associated with IgM or IgD, as previously noted by others [18]. The only rearrangement that displayed a non-bell shaped profile in all naïve fish was VH4-Cδ, which had an extra peak associated with a unique CDR3 (ARGTEYYFDY) (Figure 1B).

To analyze the variability of these profiles in a quantitative manner we used the ISEApeaks statistical analysis software [29], [30]. ISEApeaks computes the “perturbation” of a given VHC spectratype profile in comparison with a reference as described in Material and Methods in the section “CDR3 length spectratyping analysis”. Taking the average of control fish profiles as a reference, we quantified the inter-individual variability of VHC spectratype profiles among healthy fish for each isotype. These results were represented by a matrix of distance in red color scale (Figure 1C-D-E, left panels). The perturbation scores calculated in this way were low, as evidenced by their appearance in grey or light pink, indicating that the CDR3 length profiles were remarkably similar for the different healthy fish as expected for truly naïve animals belonging to the same fish clone.

We also compared the CDR3 length diversity of IgM, IgD, and IgT through an index based on the concept of Shannon entropy that takes into account both the number of peaks and the evenness of their area for each VHC combination (Figure S2B). This analysis did not reveal any significant difference of CDR3 length diversity between IgM, IgD, and IgT in the spleen of naïve fish, reflecting that these expressed repertoires were all diverse.

We then further compared the expressed IgM, IgD, and IgT repertoires to each other using ISEApeaks. For this, we computed the perturbation scores of each individual fish for a given isotype (e.g. IgM), and compared it to the average score of the three fish for a distinct isotype (e.g. IgD or IgT) (Figure S2C). This analysis revealed that VHCμ and VHCδ profiles were similar to each other for most VH, while VHCτ rearrangements were clearly distinct from both VHCμ and VHCδ profiles. These findings likely reflect the fact that μ and δ IGH transcripts can be produced through alternative splicing of the same primary transcripts, while IGHτ rearrangements utilize distinct D and J genes. This is also consistent with the fact that trout naïve B cells either co-express IgM and IgD, as known for mammalian B cells, or only IgT as previously reported [18], [21].

### Acute systemic viral infection induces strong IgM and IgT responses while the IgD repertoire remains almost undisturbed

To analyze the B cell response to a viral infection, four clonal fish (genetically identical to the healthy fish described above) were challenged two times with VHSV, on days 1 and 21. After three more weeks (day 42), all fish contained neutralizing VHSV antibodies in serum (see below). Also, FACS analysis indicated that the spleen B cells of these clonal fish contained about 75–90% IgM^+^IgT^−^ and 25–10% IgM^−^IgT^+^ B cells as previously reported. This ratio was not significantly modified after infection (Figure S3A). The B cell response was characterized at this time point after challenge (day 42) by spectratyping analysis of the expressed IgM, IgD, and IgT repertoires.

#### IgM

We first analyzed IgM that is typically associated with systemic immune responses in fish. Compared to healthy fish, many VHCμ profiles were strongly perturbed after infection, especially those associated with VH3, VH4, VH5.1, and VH9. In addition, other VH were also altered, albeit in milder way (Figure 1B and Figure S3B). To quantitatively evaluate these modifications, we calculated the ISEApeaks perturbation index for each VHC combination, comparing the profiles of infected fish to the average profiles of healthy animals (Figure 1C, middle panel). Remarkably, this statistical analysis revealed that all VHCμ profiles were significantly altered by the infection (Table 1a). To assess the inter-individual variability of this response, we then compared the VHCμ profile of each infected fish to the corresponding average profile of all infected animals. We found that the IgM response varied markedly from fish to fish (Figure 1C, right panel), implying that it contained private responses, *i.e.* responses observed only in some individuals. Despite this diversity, some peaks were amplified in all infected fish, as for example the peak for CDR3 of ten amino acids in the VH5.1-Cμ profiles, evoking a public response (Figure 1B, VHCμ panel).

**Table 1:** Statistical significance of the spectratype differences between infected and control groups (A) or between infected groups for different isotypes (B), for each VH family.

A.	Inf IgM vs ctr IgM	Inf IgD vs ctr IgD	Inf IgT vs ctr IgT
**IgVH1.1**	**0.0031** 1	0.0126	
**IgVH1.5**			0.1729
**IgVH3**	**0.0060**	0.0526	0.0148
**IgVH4**	**0.0005**	0.0784	**0.0005**
**IgVH5.1**	**0.0000**	0.1925	0.1577
**IgVH5.2**	**0.0094**		
**IgVH5.4**	**0.0080**	0.0144	0.0636
**IgVH6**	**0.0001**	0.0126	
**IgVH8**	**0.0000**	0.0269	0.0195
**IgVH9**	**0.0012**	0.0526	**0.0052**

B.	Inf IgM vs Inf IgD	Inf IgM vs Inf IgT	Inf IgD vs Inf IgT
**IgVH1.1**	0.0275	na	na
**IgVH3**	0.5221	**0.0015**	**0.0028**
**IgVH4**	**0.0000**	**0.0017**	**0.0084**
**IgVH5.1**	**0.0000**	**0.0000**	**0.0061**
**IgVH5.4**	0.4324	**0.0018**	**0.0061**
**IgVH6**	0.1371	na	na
**IgVH8**	0.0275	**0.0015**	**0.0061**
**IgVH9**	0.3323	**0.0087**	**0.0028**

1 Adjusted p-values by a Benjamini-Hochberg correction for multiple testing of an empirical Bayesian test (H0 = “perturbation” index similar in the two groups). p-values indicating a significant difference between the tested groups (Type I error level = 0.01) are in bold. For more details, see M.&M., section “CDR3 length spectratyping analysis.

#### IgD

In trout IgD transcripts are produced by alternative splicing of the IG rearrangement expressed in the μ chain, as in humans and mice (Figure 1A). Hence, if every responding B cell produces both IgM and IgD, the corresponding spectratypes should be skewed in the same way upon infection. Using ISEApeaks to compute and compare the perturbation index between IgD and IgM in infected fish (Table 1b), we found a significant difference for the most responding VH4 and VH5.1, but not for the profiles that were less perturbated in IgM after infection. Additionally, while the VHCμ profiles were extensively modified after infection, we observed only weak modifications of the VHCδ profiles after infection (Figure 1B and 1D, Figure S3B). In fact, ISEApeaks analyses revealed that the perturbations between naïve and infected fish were not significant for any of the VH for IgD (Table 1a). Since IgM and IgD profiles were determined on the same individuals, this suggests that responding B cell clones expressed IgD to a much lower level than IgM. This could be due to down-regulation of IgD expression on IgM^+^IgD^+^ B cells upon activation, as observed in mammalian B cells.

#### IgT

IgT rearrangements are independent of IgM and IgD, and carried by a distinct population of IgT^+^IgM^−^ B cells (Figure S3A). Intriguingly, the splenic IgT repertoire displayed clear perturbations compared to controls, which were statistically significant for VH4 and VH9 (Figure 1B and 1E, Figure S3B and Table 1a). However, no VHCτ (IgT) perturbation was shared by all fish, indicating the activation of distinct sets of IgT^+^ B cell clones in each fish. When comparing with ISEApeaks the perturbation index between the different isotypes from the infected fish group for each expressed VHC combination (Table 1b), all VH_i_Cτ profiles were significantly different from the corresponding VH_i_Cμ and VH_i_Cδ profiles. The VH genes involved in the IgT response did not match those dominating the IgM response, VH3Cτ and VH5.1-Cτ profiles being unmodified. Our observations indicate that IgT^+^IgM^−^ B cells can mount robust responses to systemic viral infection, in addition to their previously described role in mucosal immunity [21].

From these results, we conclude that viral challenge induces a broad IgM response in spleen, which includes public and private components, and involves all expressed VH families. We also found that IgT^+^ B cells can make a clear response in spleen, indicating their implication in systemic immunity.

### Molecular analysis of the diversity of anti-viral IgM and IgT responses in spleen through 454 pyrosequencing

To characterize the molecular diversity of this anti-viral B cell response at the CDR3 sequence level, we performed deep sequencing analyses of a number of VHC combinations involved in major (VH4 and VH5.1 for IgM; VH4 for IgT), moderate (VH1.1 for IgM; VH5.1 for IgT) or weak (VH5.4 for IgM and IgT) responses at the mRNA level. IgD was not analyzed further because of its minor contribution to the response. The sequence reads obtained through 454 pyrosequencing were analyzed by IMGT/HighV-QUEST.

Sequences encoding different V-D-J rearrangements were assembled into junction sequence types (JST) for statistical analysis (Figure S4). We hereafter refer to JST in our analysis, defined as a CDR3 amino acid sequence associated to a given (V, J) pair. As a preliminary study, we estimated the error rate to be around f = 3×10^−3^ per base pair using a known VH sequence (see methods), which was close to the estimations previously reported varying between 0.4 and 1% [23], [31]. Using this average error rate per site, we corrected the number of reads for each junction nucleotide sequence (JNS) by adding the sequences lost and subtracting those gained due to erroneous mutations introduced by the PCR and sequencing procedure (Figure S5). These corrected datasets were then translated and aggregated into corrected JST datasets, which were analyzed in parallel to the observed, unprocessed JST datasets.

For each VHC sequencing analysis, we first classified the observed JST according to their CDR3 size to produce “virtual spectratypes”. These “virtual spectratypes” were consistent with the PCR-based spectratypes (Figure S6), indicating that the deep sequencing analysis of the PCR products did not add biases in IgM and IgT CDR3 length distributions, as previously noted for human IgM and IgA in [26].

#### IgM

**Figure 2:**
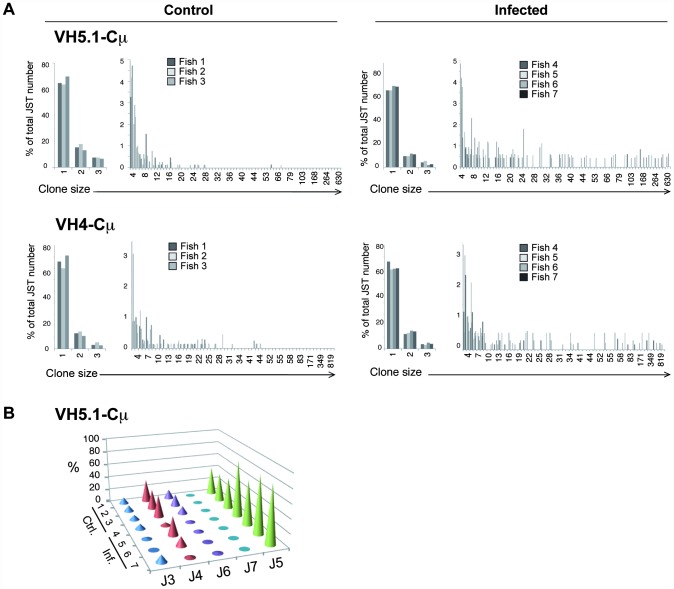
454 pyrosequencing-based analysis of the spleen IgM response to VHSV. A. Normalized distributions of JST observed n times in the sequence datasets from control and virus infected fish are represented for VH5.1 and VH4. Large clonal expansions are indicated by high number of occurrences of expressed junction types in infected animals. (Total number of sequences on which each distribution is based: VH5.1Cμ control: 4306; VH5.1Cμ infected: 6245; VH4Cμ control: 4107; VH4Cμ infected: 7755). B. J usage among VH5.1Cμ transcripts in control and VHSV infected fish.

In healthy fish, 90 to 99% of the JST were found less than 5 times for both unprocessed and corrected datasets (Figure 2A and Figure S7B), indicating a high degree of repertoire diversity in naïve IgM^+^ B cells. The viral infection induced a dramatic shift in the expressed IgM repertoire, as shown by the increased frequency of VH5.1Cμ transcripts carrying JH5 (Figure 2B), and of VH4Cμ transcripts carrying either J5 or J7 (Figure S7A) compared to healthy fish. In infected fish, some JST were found at a high frequency (up to 800 times out of 2200 JST for VH4Cμ), while in naïve animals the most abundant JST were seldom found more than 50 times (Figure 2A; Figure S7B), both in unprocessed and in corrected datasets. The frequency distributions from control and infected fish were significantly different not only for the highly responding VH4 and VH5.1, but also for VH5.4 (Table 2). As a negative control, we compared all distributions within control fish (control-control) or within infected fish (infected-infected) for each VH. These tests never provided any significant difference among controls, and only once among infected (*p* = 4.9%), further supporting that the differences between JST distributions of infected and control fish were real (Table 2 and Figure S8). The same tests performed on corrected datasets led to similar conclusions (Figure S5C).

**Table 2:** Kolmogorov-Smirnov tests on junction type distributions from 454 pyrosequencing of spleen VHCμ expressed rearrangements.^1^

	Combination of fish subjected to the KS test^2^	Two-sided^3^ p-values	One-sided^4^ p-values
	**Infected vs controls**		
VH4_Cμ	All Ctrl vs All infected	1.41E−03 **	7.03E−04 ***
VH5.1_Cμ	All Ctrl vs All infected	1,52-E04 **	7.62E−05 ***
VH5.4_Cμ	All Ctrl vs All infected	NS	2.95E -02*
VH4_Cμ	One Ctrl vs One Inf (all combinations)	All ** but {2 vs 5} *	All **
VH5.1_Cμ	One Ctrl vs One Inf (all combinations)	All **	All **
VH5.4_Cμ	One Ctrl vs One Inf (all combinations)	All* but {1 vs 5;1 vs 6;1 vs 7; 3 vs 6; 3 vs 7}	All* but {1 vs 6;1 vs 7;3 vs 6}
	**Within controls:**		
VH4_Cμ	all combinations	NS^5^	NS
VH5.1_Cμ	all combinations	NS	NS
VH5.4_Cμ	all combinations	NS	NS
	**Within infected:**		
VH4_Cμ	all combinations	All NS but {4 vs 6} *	All NS but {4 vs 5;4 vs 6; 4 vs 7; 4 vs 5_7; 4 vs 6_7} *
VH5.1_Cμ	all combinations	NS	NS
VH5.4_Cμ	all combinations	All NS but {4 vs 6; 4 vs 7; 5 vs 6; 4 vs 6_7 } *	All NS but {6 vs 4_5; 6 vs 4_7} *

1 The procedure to compute KS tests and to aggregate JST from different fish is detailed in Figure S8. Aggregated distributions are denoted by «_» linking the corresponding fish number. Control fish are 1–3 and infected fish 4–7.

2 KS test was applied to different combinations of distributions: (1) All control (aggregated) versus all infected (aggregated) (2) One Ctrl vs One Inf (all combinations): each control versus each infected individual distribution; (3) All combinations: each individual or aggregated distribution versus all other individual or aggregated distributions, within control or infected respectively. For example {1 vs 2; 1 vs 3; 2 vs 3; 1 vs 2_3; 2 vs 1_3; 3 vs 1_2 } within control fish.

3 ‘Two-sided’ analysis tests if the distribution in Infected samples is equal to the distribution of Control samples (i.e.H0, null hypothesis of the KS test). P-values< 5% *; <1% **; <0.1% *** indicate that the distributions are significantly different.

4 ‘One-sided’ analysis tests if the distribution in Infected samples is shorter than in Control samples (H0). P-values<5% *; <1% **; <0.1% *** indicate that the distributions in control fish are significantly shorter than in infected fish.

5 NS: non significant, i.e. H0 = “equal distribution” is not rejected.

**Figure 3:**
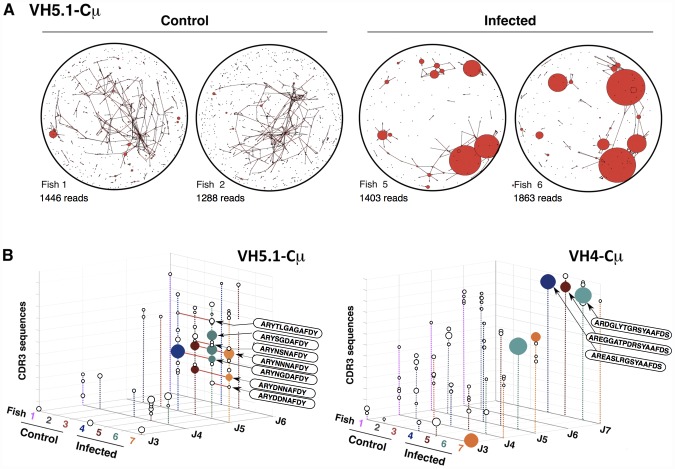
Representation of clonal complexity of spleen VH5.1-Cμ and VH4-Cμ responses. A. The program Pajek distributes different JST on the surface of a sphere. Large clones are represented by red beads with a diameter proportional to the number of junctions present in the sequence dataset, and connections link junctions that differ at only one position. Each vertex in the network refers to a different CDR3 amino acid sequence, and the size of the vertex represents the number of identical sequences. Two vertexes are therefore connected if the CDR3 amino acid sequences they represent differ at one and only one position at the AA level. VH5.1Cμ JST distribution is represented. The total number of sequence reads on which each graph is based is indicated. No global re-normalization was performed. B. 3D-Representation of VH5.1-Cμ and VH4-Cμ clonal expansions found in control and infected fish. The three axes enumerate observed V amplification primer (x), J (y), and CDR3 sequences (z) values, allowing comparison between fish. The size of the sphere at each point corresponds to the number of reads matching a particular JST. JST for which H0 was not rejected are represented in fish–specific colors; other repeated JST are also shown as white spheres to illustrate the structure of the dataset. Large clonal expansions of identical junctions found in different individuals are linked in red and the IMGT-CDR3 AA sequences indicated inside a plain ellipse. These graphs represents clonal expansions revealed by sequence types present more than 15 times among datasets of the following sizes (reads with productive junctions: VH5.1Cμ control = 1446, 1288, 1572; VH5.1Cμ infected = 1442, 1403, 1863, 1537; VH4Cμ control = 1428,1441,1238; VH4Cμ infected = 2213, 1212, 2603, 1727). No global re-normalization was performed between datasets.

To better visualize the structure of IgM response in infected fish, we used the network analysis and visualization program Pajek [32] (Figure 3A and Figure S10). Pajek represents each JST by a bead (vertex) of diameter proportional to its number of occurrences, and it links by a line the vertexes corresponding to JST differing by only one amino acid. In each infected fish this analysis revealed the presence of large vertexes linked by numerous connections, which were absent in naïve animals (Figure 3A). To test if JST comprising high numbers of reads did not represent sampling artifacts, we modeled the sequencing process as a successive sampling in urns (Figure S9). This analysis suggested that at least the high read numbers of the most abundant JST were not explained by sampling artifacts. Highly repeated JST for which the null hypothesis “H0 = the JSTj is expressed by a responding amplified clone” was not rejected were represented in color on Figure 3B; while other repeated JST were shown as white spheres to illustrate the structure of the dataset. When comparing the JST expanded in the different infected fish (Figure 3B), it appeared that some similar rearrangements were expanded in all animals. In particular, related VH5.1-J5 rearrangements with CDR3 of 10 amino acids (IMGT numbering) were present in all fish. The CDR3 ARYNNNAFDY was the most frequent, being found in more than 20% of VH5.1Cμ rearrangements in infected fish #5, 6 and 7. A number of other related JST were found repeated, with small or polar amino acids: ARYNNDAFDY (infected fish#6 and 7), ARYDNNAFDY (infected fish#5 and 7), ARYNSNAFDY (infected fish#6 and 7), ARYNNVAFDY (infected fish#4), ARYDDNAFDY (infected fish#5 and 7), ARYNTNAFDY (infected fish#6) ARYNGDAFDY (infected fish#4 and 6), ARYSGDAFDY (infected fish#4 and 6), and ARYNGRAFDY (infected fish#4). The three first JST of this list were found in several fish, and differed from the most frequent JST by only one conservative substitution. Such expansion of a number of similar junctions is typical of “public” responses in mammals [33], [34]. To ascertain that this “public” response was not a sampling artifact, we showed that the null hypothesis “H0 = altogether the public JST represent amplified clones” was not rejected in infected fish, while the null hypothesis “H0 = altogether the public JST are expressed by non-amplified clones” was not rejected in control fish (Figure S9). It is important to note that if even only one of the “public” JST would have been found even in only one of the control fish, H0 would have been rejected and the “public” response falsified.

#### IgT

**Figure 4:**
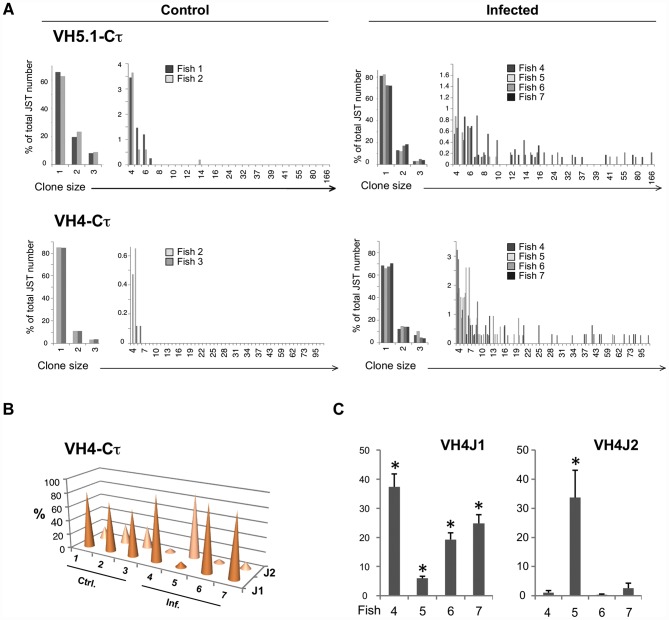
454 pyrosequencing-based analysis of the spleen IgT response to VHSV. A. Normalized distributions of JST observed n times in the sequence datasets from control and virus infected fish are represented for VH5.1 and VH4. Large clonal expansions are indicated by high number of occurrences of expressed junction types in infected animals. (Total number of sequences on which each distribution is based: VH5.1-Cτ control: 2181; VH5.1-Cτ infected: 4444; VH4-Cτ control: 1437; VH4-Cτ infected: 7528). B. J usage among VH4-Cτ transcripts in control and VHSV infected fish. C. Relative mRNA expression of VH4 rearranged to J1 and J2 in the spleen of infected fish compared to control fish. Results were normalized to EF-1α and relative expression was calculated using the Pfaffl method. Each value is the fold increase, expressed as the average of the results of comparison of one infected fish with the results of 3 control fish. Control values are set at 1.* indicates a significant increase with respect to controls (p<0.05).

In healthy fish, 90 to 99% of JST from the observed or unprocessed datasets were found less than 5 times, indicating a high degree of repertoire diversity in naïve IgT^+^ B cells (Figure 4A and Figure S5). Very few JST were found more than 6 times (Figure 4A and Figure S7C). VHSV infection led to the accumulation of large VHCτ clones in the spleen of infected fish, as revealed by the significant shift of JST distributions (Figure 4A and Figure S7C, Figure S5). As for IgM, JST frequency distributions of infected fish significantly differed from those of control fish (Figure S8). When we compared combinations of JST frequency distributions within control fish (control-control) or within infected fish (infected-infected), the Kolmogorov-Smirnov test generally did not reject the null hypothesis (H0 = equal distributions). However, the distribution of VH4Cτ from infected fish#5 was found significantly different from some VH4Cτ distributions from other infected fish, reflecting its unique modification after infection. Interestingly, several VH4Cτ JST with large numbers of reads were encoded by two different nucleotide sequences, supporting the idea they represented the product of antigen-driven clonal expansions (Figure S11). Also, the viral infection affected the representation of the J1 and J2 genes compared to control fish (Figure 4B). The modification of IGHJ usage observed by deep sequencing in VH4Cτ was confirmed by QPCR (Figure 4C).

**Figure 5:**
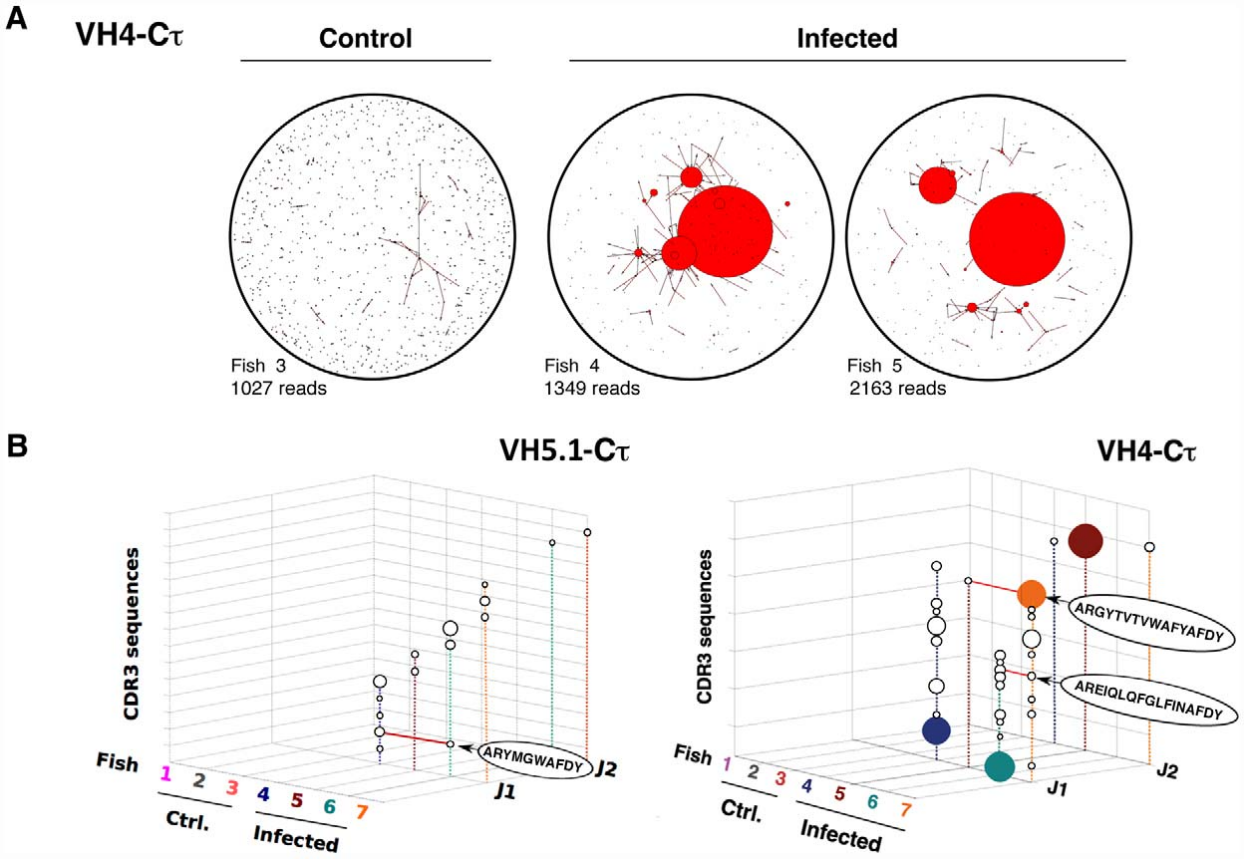
Representation of clonal complexity of spleen VH5.1-Cτ and VH4-Cτ responses. A. Pajek representation of VH4-Cτ JST diversity and distribution in control and infected fish. total number of sequence reads on which each graph is based is indicated. B. 3D-representation of VH5.1-Cτ and VH4-Cτ clonal expansions found in control and infected. Clonal expansions of identical junctions found in different individuals are linked in red and the IMGT-CDR3 AA sequences indicated inside a plain ellipse. JST for which H0 was not rejected are represented in fish–specific colors; other repeated JST are also shown as white spheres to illustrate the structure of the dataset. These graphs represents clonal expansions revealed by sequence types present more than 15 times among datasets of the following sizes (reads with productive junctions: VH4Cτ control = 198, 212, 1027; VH4Cτ infected = 1349, 2163, 1964, 2052; VH5.1Cτ control = 1202, 791, 188; VH5.1Cτ infected = 1344, 983, 1044, 1073). No global re-normalization was performed between datasets.

The structure of the IgT response in each challenged fish was visualized with Pajek, showing that IgT was associated with fewer large vertex than IgM (Figure 5A and Figure S10). No public response was identified among τ JST (Figure 5B), but our sampling model indicated that the largest IgT JST corresponding to private responses were not due to sampling artifacts (Figure 5 and Figure S9).

Taken together, these results confirmed that fish adaptive B cell responses are mediated mainly by IgM as suggested by spectratypes. A public response containing a number of similar CDR3 differing by conservative amino-acid substitutions was characterized, as typically reported in mammalian public responses. Additionally, IgT contributes to the response with large private expansions observed for a number of VH subgroups.

### Increased expression of secreted IgM and IgT in the spleen of virus-infected fish

**Figure 6:**
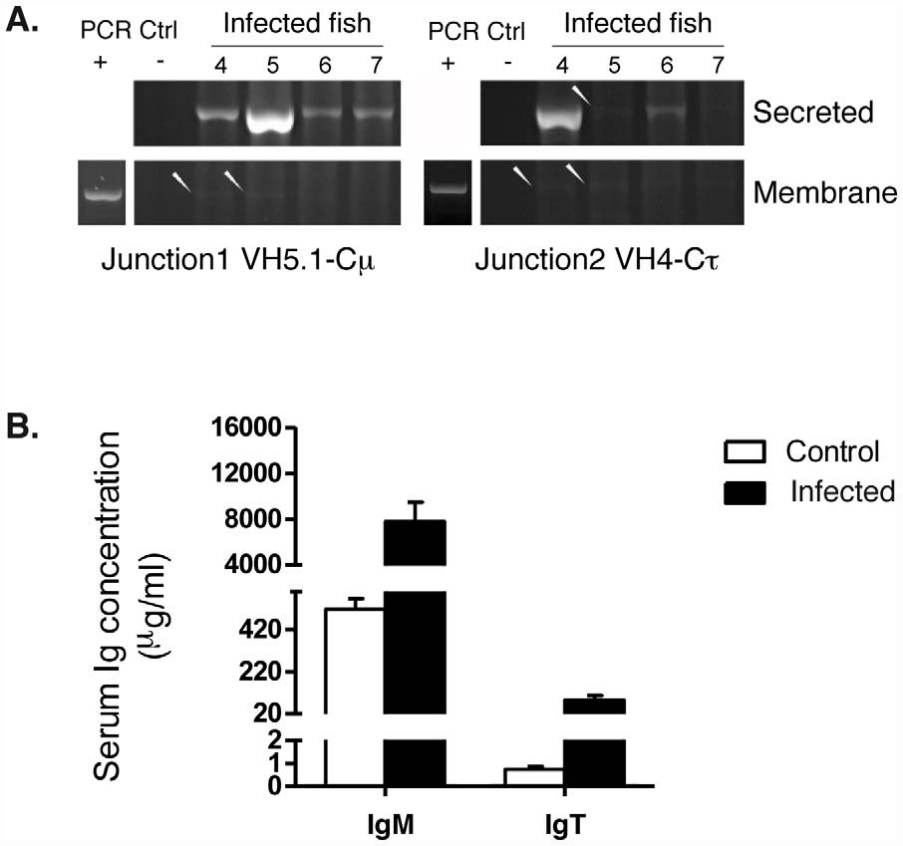
Increased expression of secreted IgM and IgT following virus infection. A. Typical results produced by specific amplification of secreted and membrane-bound transcripts containing selected JST from infected individuals (a–d). JST corresponding to the IgM public response (junction 1, VH5.1Cμ: ARYNNNAFDY) and JST corresponding to a well-expanded IgT V4Cτ response (junction 2, VH4-Cτ: ARDGLYTGRSYAAFDS) are shown. Non-expanded JST could never be specifically amplified. PCR positive controls (PCR Ctrl +) were amplified from the specific products cloned and sequenced. PCR negative controls (Ctrl −) represent reactions without cDNA template. B. Serum IgM and IgT concentration in control and infected fish.

An important question about large clonal IgM and IgT expansions found in the spleen concerned the capacity of the corresponding B cells to produce secreted Abs. To clarify this issue, we selectively amplified either membrane-bound or secreted transcripts using reverse primers located in the transmembrane exon or in the 3′ end sequence specific for the secreted isoform. First, we used primers specific for JST found expanded in our 454 sequencing after infection (Figure 6). The secreted isoform was always expressed at a greater magnitude than the membrane isoform for μ chains of IgM and for τ chains of IgT. Secreted μ transcript for the VH5.1J5 public response was also amplified from all infected fish tested, while the VHx4J1 private response was only detected in some individuals. No amplification was observed with primers targeting JST that were not expanded in the sequence datasets, or from control animals.

We next quantified the whole population of secreted versus membrane IGH μ (or τ transcripts – potentially including all non-responsive clones and sterile transcripts – using forward primers specific of the 3′ exon for each isotype, and the reverse primers described above. Our QPCR results showed that even at the B cell whole-population level, the fold change after VHSV infection is higher for transcripts encoding secreted IGH μ and τ than for those encoding membrane-bound IG (2.0+−0.66, and 3.7+−0.94 times more, respectively) (Figure S12). With regards to the different τ subtypes, τ1 and τ3 were involved in the response while τ2 was not expressed. No induction was seen for IGH δ transcripts (Figure S12). Overall, these results confirm that the expansions of VHCμ or VHCτ observed in the spleen following infection mainly represent transcripts encoding secreted IGH chain. Hence, the spleen contains virus specific Ab producing cells at this stage of the response.

Finally, we estimated the serum concentration of IgM and IgT in control and infected animals (Figure 6B). After infection, fish serum contained in average 7853 µg IgM per ml and 85 µg IgT per ml, representing an increase of 15 and 117 times, respectively, in comparison with healthy fish. These results confirm that both IgM and IgT participate to the Ab response against the virus. However, the serum IgM concentration was ∼100 fold higher than that of IgT, thus, IgM overwhelmingly represents the main responding isotype.

### Neutralising activity of the serum from infected fish is mainly mediated by IgM

Neutralising antibodies are of critical importance for the fish defense against VHSV infection, and their presence is highly correlated to the protection after vaccination. To address the question of the respective importance of serum IgM and IgT in virus neutralization, we performed inhibition assays of VHSV neutralization using either anti-IgM or anti-IgT Abs. For this purpose, we used the anti-IgM monoclonal Ab 1.14 [35], and a polyclonal anti-IgT antiserum [21]. As shown in Table 3, we found that the neutralizing activity of the serum from vaccinated fish was mainly due to IgM, while IgT had only a minor contribution if any. The inhibition of neutralization by the anti-IgM Ab was dose dependent, and reached a maximum at the dilution of the serum in the assay (data not shown).

**Table 3:** Relative contribution of IgM and IgT to the VHSV neutralising response of rainbow trout.

	Fish4	Fish5	Fish6	Fish7
Neutralisation by serum Abs^1^	75	62	71	55
Inhibition by anti IgM (1.14)^2^	77.4	71.1	92.6	100
Inhibition by anti IgT (polyclonal)^2^	0	0	15	3.3

1 Neutralization efficiency measure as the percentage of PFU reduction when serum is added.

2 Relative inhibition of neutralization by anti IgM or anti IgT Abs, normalized to the neutralizing activity of each serum (%).

All tests were performed in duplicates.

## Discussion

In this work, we characterized the clonal structure of the rainbow trout IgM, IgD and IgT response against a systemic viral infection. IgM represented the main response in the spleen, with high clonal complexity and all IGHV subgroups involved. A striking result of our study is the implication of B cells expressing the mucosal isotype IgT in the spleen response to the virus, with distinctive clonal expansions identified by amplified junctions in IGH τ transcripts. In contrast, the IgD expressed repertoire was modified only to a modest extent, suggesting that either activated B cells loose IgD expression as in mammals [36] or that IgD^+^ responding B cells leave the spleen and re-locate in other territories. Based on combined CDR3 length spectratyping and deep sequencing of IGH transcripts at one time point during the response to secondary virus infection, our approach does not provide a kinetic description, nor directly addresses the specificity of the antibodies corresponding to CDR3 expansions identified here. However, the correlation of such expansions with viral infection as well as their frequency and distribution among infected fish provide a first comprehensive view of a spleen antibody response to a virus in fish, and more generally in a vertebrate.

A global understanding of the mechanisms that determine the structure of the B cell expressed available repertoire requires a comprehensive survey of Ab diversity during the maturation of the immune system and along immune responses. A widely used method developed for large-scale IG and TcR V domain repertoire analysis consists in CDR3 length profiling, leading to 6–15 peak spectratypes for each V/C or V/J combination [27]. This approach of individual repertoire variations can be coupled with statistical analysis [37], and has been frequently used to characterize T and B cell responses [33], [37]–[39]. However, it does not provide a full description of the junctions. An important breakthrough towards a complete description of immune repertoires was recently made with the usage of 454 GS FLX high-throughput pyrosequencing for the direct analysis of whole B cell diversity of individual zebrafish [23]. A few studies based on the same approach have been published on human B and T cells [24], [40]–[42]. While it is well-known that pyrosequencing induces artefacts such as mutations in poly C/G stretches, and artificial duplications of sequences [43], we show here that 454 pyrosequencing and CDR3 length spectratyping lead to fully consistent and comparable results from the same samples. These results are in agreement with the observations of Ademokun *et al.* [26], who followed a similar approach to analyze the modifications of the human IgM and IgA expressed repertoires after vaccination against pneumococcal pneumonia in young and elderly. Importantly, our analysis assessing for each JST the number of sequences lost or gained due to technical error confirmed that the structure of our datasets was in the same line as in previous reports. Also, our sequencing error rate allowed the identification of V and J genes by the IMGT/VQUEST annotation pipeline. To our knowledge, this study is the first comprehensive description of a B cell response to a viral pathogen.

Combining CDR3 spectratyping and pyrosequencing, we show here that the fish Ab response primarily involves IgM. A few very large IGHμ clones were preeminent in the response to the virus, likely targeting a small number of dominant epitopes. These large sets of amplified JST are difficult to explain by sampling artifacts of the deep sequencing. Indeed, our model of repertoire description by sequencing indicates that at least the largest JST represent true clonal expansions. These JST expansions were either private – ie found in only one fish- or public – *ie* found in (almost) all individuals. An IgM response was identified, involving VH5.1 rearranged to J5, which represented a typical public response consisting in a set of highly similar junctions present in all fish. The observed public responses were strongly expanded and could be inferred from the spectratyping, corresponding to high peaks found in all infected fish. These results suggest the presence of a common pool of pre-existing spleen B cells among which the public IgM response to VHSV could be recruited, a notion that is supported by findings in other models. Thus, in the large-scale sequencing of the naïve zebrafish IgM VH domain repertoire, convergences have been identified in the normal repertoire, with identical VHC sequences retrieved in different individuals at significant frequencies [23].

Strikingly, all VHCμ profiles were significantly altered after infection, indicating that the IgM response to the virus comprised diverse components likely targeting many epitopes. Since VHSV is a natural pathogen of rainbow trout, a possible explanation of this observation is that natural selection has favored multicomponent Ab responses to this highly variable negative strand RNA virus. Another non-exclusive explanation of this observation would be a non-specific polyclonal B cell stimulation by the viral infection. Such polyclonal stimulation could be mediated by different mechanisms including direct activation by viral components through pathogen recognition receptors, bystander activation by activated T cells, or viral superantigens. In the mouse, a number of mitogens from different origins [44]–[46] are very effective in inducing B cell polyclonal activation [47], [48]. Moreover, it has been known for a long time that salmonids B cells stimulated by mitogens not only proliferate, but also increase their global production of antibodies [49], [50]. Interestingly, the nucleocapsid of the Rabies virus, a rhabdovirus related to VHSV, acts as a B cell superantigen in human and induces polyclonal B cell activation [51]. Whether such polyclonal B cell stimulation has deleterious or beneficial impact on the host is still a matter of debate [47].

For a comparable number of sequences analyzed, no public response was found for IgT, not even for the VH4-Cτ combinations showing highly skewed spectratypes in all fish. In contrast to IgM, IgT CDR3 length spectratypes were found significantly skewed after viral infection for only two VH. These results indicate that the number of pre-existing clones directed against the virus was higher among spleen IgM^+^ B cells compared to IgT^+^ B, which is in accordance with the respective frequency of IgM^+^ and IgT^+^ B cells in the spleen of control fish. Perhaps, if IgT has been evolutionarily selected mainly for mucosal defense, “pre-existing” specificities in the available repertoire may be focused on mucosal pathogens. However, the biochemical properties of the V-D-J junctions analysis *in silico* in the whole set of IgM and IgT sequences from healthy and infected fish identified no clear difference of isoelectric point, charge or gravy between the two isotypes (Figure S13).

Since IgT mainly protect gut mucosa, the question of the role of IgT expansions in the spleen of virus-infected animals is intriguing. The dominant expression of the secreted form of the IgHτ transcripts in infected animals and the increase of serum IgT concentration upon infection indicate that such spleen IgT^+^ cells likely produce Abs. A first possibility would be that Ab secreted into the blood by such spleen IgT^+^ clonal expansions would indeed contribute to the antiviral defense of the whole organisms including mucosa. Alternatively, virus specific IgT^+^ B cells may get activated and proliferate before migrating to the mucosal territories, and may be found in the spleen as transiting cells. These observations are reminiscent of the complex roles of IG classes and subclasses in humans. While IgA is the most abundant Ab class in the mucous and other secretions from epithelia, it is also present – mostly the subclass IgA1- in the serum where it can bind to antigens and mediate Ab-dependent cellular cytotoxicity (ADCC), respiratory burst, degranulation and phagocytosis through binding to the FcαRI receptor [52]. Reciprocally, similar to IgA, human IgM pentamers can be secreted through the gut epithelium into the gut lumen, whereas IgG can be found in colostrum and milk. Also, IgA^+^ B cells recirculate from the gut lymphoid structures, joining the bloodstream and homing back to the gut mucosa [53]. Thus, the classical division of IG classes and subclasses into systemic or mucosal Abs cannot be viewed in absolute terms in mammals, and this apparently applies to fish too.

In healthy fish, we found B cell expansions in the spleen of control trout as reported in zebrafish [23]. While it has been reported that rainbow trout spleen contains mostly resting B cells, and few Ab producing cells [54], our results indicate that spleen comprises indeed a small proportion of such clones, expressing either IgM or IgT. The distributions of amplified JST indicate that clones responding to the virus are generally larger than the plasma cell clones present in the spleen of control fish, which is also in good accordance with the spectratyping profiles.

A tentative estimation of the relative proportion of large responding IgM^+^ and IgT^+^ clones in the B cell population was made from sequence data produced in healthy and infected fish. Considering that an Ab-producing cell produces 100 to 1000 more mRNA than a resting B cell [55], [56], the proportion of expanded cells in healthy fish was estimated from the proportion of sequences present 5 times or more in the 454 datasets, for a given number of available reads (Table 4, Figure S14). In pyrosequencing, the emulsion-based PCR from individual molecules makes possible that a sequence may appear a few times instead of only one, making clonality comparisons comparative rather than absolute [40]. We therefore considered that the observation of 1–5 occurrences – instead of 0–1 – did not reflect the precise frequency of a JST, but could rather represent variable amplification from a unique sequence, due to a side effect of the technique. We therefore considered that this represented resting cells, and that higher numbers of occurrences denoted Ab-producing cells (Figure S14). The estimated frequency of Ab-producing cells in the spleen of control animals varied with the rearrangement considered, but for a given V, was lower for IgT^+^ cells. Thus, our calculation predicted ≈100 times more IgM-producing cells than and IgT- producing cells in control fish for VH4, and around 10 times more for VH5.1 and VH5.4 (Table 3). Calculations from corrected datasets led to similar results (Figure S14). After infection, the predicted frequency of Ab-producing cells increased generally 2 to 9 times, with the exception of VH4Cτ for which the final frequency reached the same order as for the other combinations but from a very low initial value (Table 3). Overall, these estimations suggest that the viral infection significantly increases the total frequency of large B cell clones, and are in accordance with the idea that IgM represents the dominant class produced in the spleen in control as well as in infected animals [27]. Since all expressed VH subgroups were involved in the response to the virus, this represents a considerable shift of the activated B cell subset. It has been reported that in naïve fish IgM^+^ and IgT^+^ B cells represent respectively 75–90 and 10–25% of the whole spleen B cell population, and that IgT concentration in plasma of naïve fish is approximately 1000 fold lower than that of IgM [21]. This would suggest an even greater difference of frequency of IgM^+^ versus IgT^+^ Ab-producing cells at the scale of the whole organism. In the clonal fish used in this study, we found the same proportion of IgM^+^ and IgT^+^ B cells, and it was not significantly modified by the infection. Considering that plasmablasts and plasma cells express membrane Ig at very low level [21], this observation is well consistent with an important contribution of these subsets to the response seen at the transcript and serum Ab levels. At any rate, inhibitions of viral neutralization by fish serum using anti IgM or anti IgT antibodies clearly showed that IgM is of foremost importance for the antibody-mediated protection against the viral infection.

**Table 4:** Estimated frequencies of resting B cell and Ab producing cells from clonal expansions detected in 454 datasets.^1^

		Resting B cells^2^	Ab-producing cells
VH4_Cμ	Control	9.99E−01	7.03E−04
	Infected	9.98E−01	1.72E−03
VH4_Cτ	Control	1.00E+00	4.20E−06
	Infected	9.98E−01	1.95E−03
VH5.1_Cμ	Control	1.00E+00	4.26E−04
	Infected	9.96E−01	4.24E−03
VH5.1_Cτ	Control	1.00E+00	7.79E−05
	Infected	1.00E+00	3.12E−04
VH5.4_Cμ	Control	9.95E−01	5.09E−03
	Infected	9.86E−01	1.42E−02
VH5.4_Cτ	Control	9.99E−01	1.10E−03
	Infected	9.96E−01	4.09E−03

1 The procedure to compute these frequencies is detailed in Suppl. Material #14.

2 Clonal expansions were considered when a sequence type was detected 5 times or more in the datasets. A normalization procedure was followed to allow comparison among datasets for a given VH (see Suppl. Material #14).

Previous studies of trout Ab response to TNP keyhole limpet hemocyanin (TNP-KLH) using affinity-based immune-partitioning assays have shown that a first low affinity followed by an intermediate/high affinity population that persists longer while high affinity Abs appears much later after week 15 post-immunization and are expressed at higher concentrations. These results indicate that an increase of Ab affinity indeed occurs in fish, being slow but very significant [57], [58]. While the existence of somatic hypermutation in fish is now well established [59], its importance for affinity maturation is still not fully appreciated. In fact, it is generally accepted that in the absence of refined modes of selection of late-developing clones, B cells in which somatic mutants lead to a higher Ab affinity are not quickly amplified within the population [4]. Jiang et al. have analyzed the secondary IGH repertoire of the developing zebrafish using deep sequencing and found that average numbers of mutation in highly abundant IGH sequences increases with age [60]. Our results are difficult to compare directly with the simple antigen immunization since we analyzed antibody repertoires during the response to secondary infection. However, the large IGH expansions we observe in the spleen likely represents maturating plasma cells producing intermediate/high affinity antibodies, before migration into the anterior kidney. Somatic hypermutation analysis from our sequence data was hampered by the lack of knowledge about the VH genomic repertoire of the clonal fish we used and by the intermediate sequencing depth of our datasets. With well-annotated *igh* loci in the coming rainbow trout genome, further studies combining affinity assessment and deep sequencing of IGH transcripts will make it possible to correlate affinity maturation and somatic hypermutation.

Regarding the IgD, complete IGHD transcripts were expressed at very low level in the trout spleen, even after virus infection. Importantly, the few perturbations observed in IgD profiles after infection were not statistically significant. Since amplified peaks were absent in VHCδ profiles of infected fish even when they were present in the corresponding VHCμ profiles, we conclude that the great majority of responding clones found in spleen do not express IgD. Hence, either the majority of responsive IgD^+^ cells migrate out of the spleen, or pathogen-specific B cell activated by their specific antigen differentiate into IgD^−^ plasma blasts/cells. While it has been recently reported that B cells expressing IgD are found in significant numbers in pronephros and gills [61], we did not find a higher expression of IgD in gut, gill or pronephros after virus infection and we would therefore favor the second hypothesis. In fact, resting B cells co-express IgM and IgD in human, mouse and catfish, correlating with the conserved respective locations of the IGHM and IGHD genes in the IGH locus. While human IgD^+^ B cells have been described in the respiratory tract of human, and an IgM^−^IgD^+^ B subset is found in channel catfish [20], our work confirms that such cells are apparently not present in the spleen of rainbow trout [21]. In fact, the only hint of an IgD clonal expansion we found was the presence of an extra peak in VH4Cδ profiles in all tested animals, containing the amplified CDR3 ARGTEYYYFDY. This recurrent expansion present even in control animals could be due either to the recognition of an environmental pathogen/antigen, or may constitute an invariant receptor following a non-classical selection pathway. In fact, IgD expression both in control and virus infected fish appears to be almost subsidiary. The discovery of pathogens/antigens specifically eliciting IgD response would greatly improve the knowledge of its biological significance. While the structure of fish IgD is quite divergent from mammalian IgD, the absence of IgD clonal expansion triggered by VHSV infection reminds the down regulation of this isotype on activated B cells in mouse and human.

While differences in microenvironments in which B cell responses develop in fish and mammals apparently do not enforce dramatic changes in the structure of B cell response, our observations are quite parallel to what was observed in human and mice. As a rhabdovirus, the VHSV is a typical highly cytopathic virus with a surface densely packed with multiple copies of a unique glycoprotein (G), as for VSV or rabies virus. Such viruses elicit high affinity, protective Ab responses with V regions frequently encoded in the germline [62]. In human, IgG antibodies produced against rabies virus express diverse VH genes and targets both G and the ribonucleoprotein complex, while the pre-immune IgM binding the virus express mainly VHIIIb [63], [64]. Interestingly, this response not only comprises IgG but also the mucosal isotype IgA [63], [64].

### Conclusion

This work demonstrates the appearance of large clones of IGH junctions after viral infection, that likely represent the generation of typical polyclonal IgM and IgT B cells responses to a virus infection in fish. In the spleen, IGHμ shows a strikingly diversified response involving all VH subgroups with some very large public and private clones. IGHτ encoding mucosa-specialized IgT also show clonal expansions expressing secreted isoform, but at a lower scale. IgD does not seem to be affected by the response. Further characterization of B cell response to simple antigens will help understanding the respective importance of specific clonal expansions and bystander polyclonal activation. Ig transcripts from sorted B cells that bind a fluorescent antigen could be subjected to deep sequencing or single cell (RT)PCR to link directly junctions to the fine specificity of the antibodies.

While new variations of fish IG have been recently described like catfish V-less IgD [20] and IgT/IgM chimeric molecules in cyprinids [65], our results emphasizes the capacity of repertoire studies to uncover functional features of antibodies. Additionally, fast progress in sequencing technology and IG annotation open the way to *individual* and *comprehensive* descriptions of Ab repertoires, and monitoring using high-throughput sequencing will likely become paramount for health management in aquaculture as well as human medicine.

## Materials and Methods

### Viral model: fish, immunization protocols and sampling

Rainbow trout homozygous clone B57 [66] were raised in the fish facilities of Institut National de la Recherche Agronomique (INRA, Jouy en Josas, France). Two years old adult fish were placed in individual aquaria kept at 16°C. Immunization and virus challenge were performed using the attenuated 25–111 variant of strain 07–71 of VHSV [7] through intramuscular injection. A first sub-lethal dose of 10^5^ PFU/fish was applied to each fish. This infection usually leads to a good protection against a subsequent lethal infection. Three weeks later, fish received a second injection of the 25–111 virus (5×10^7^ PFU/fish) and samples were collected 3 weeks later. Control fish were left untreated. Trout were sacrificed by overexposure to 2-phenoxyethanol diluted 1/1000. Blood was extracted and let to clot at 4°C overnight for serum extraction. Tissues were removed, frozen in liquid nitrogen and stored at −80°C to use in RNA preparation. Serum extraction was performed by centrifugation at 200× g for 10 min, supernatants were collected and centrifuged at 1000× g for 20 min. Serum was frozen at −20°C to use in titration assays and Ig concentration measurement. For flow cytometry analysis of spleen leukocytes, fish were sacrificed by overexposure to 2-phenoxyethanol diluted 1∶1000. The spleen was removed aseptically and cells from the spleen of a single fish were deposited on a Ficoll solution (Lymphocyte separation medium [d = 1.077]; Eurobio, Les Ullis, France) and centrifuged 10 min at 900 g. The leukocyte fraction was collected at the Ficoll-medium interface.

### Ethics statement

All animals were handled in strict accordance with good animal practice as defined by the European Union guidelines for the handling of laboratory animals (http://ec.europa.eu/environment/chemicals/lab_animals/home_en.htm) and by the Regional Paris South Ethics committee, and all animal work was approved by the Direction of the Veterinary Services of Versailles (authorization number 78–28).

### RNA preparation and cDNA synthesis

Total RNA was individually prepared from spleen by disruption in TRIzol reagent (Life Technologies, Cergy-Pontoise, France) using 1/1.2 mm ceramic beads (Mineralex, France). Disruption protocol was 2 pulses of 15 sec at 6000 rpm in a Precellys tissue homogenizer (Bertin Technologies, France). The whole spleen was used for mRNA preparation. We thus ensured to analyze all B/plasmablast/plasma cells subsets and to avoid any biases due to regional concentration of responding B cells or to differential density of B cell subsets, which may affect the outcome of density gradient. Total RNA was purified and DNase treated using QIAgen RNA extraction kit. RNA (2 µg) was reverse transcribed into cDNA using Superscript II Reverse Transcriptase (Invitrogen Life Technologies) with 2.5 µM oligodT_25_ primer in a final volume reaction of 20 µl.

### CDR3 length spectratyping analysis

The spectratyping of TcRB CDR3 length (Immunoscope analysis) first developed for mouse or human [27] was previously adapted for rainbow trout [7]. The diversity of IG transcripts was studied following a similar approach. A first amplification (PCR1) using a forward primer specific for a subgroup or a set of IGHV genes in combination with a reverse primer Cμ, Cδ or Cτ specific for IGHM, IGHD or IGHD genes was performed as follows: 1 µl cDNA was used as template for PCR1 using 0.4 mM of each dNTP, 0.4 µM of each primer (forward: VH_family specific_, reverse: C_isotype specific_), and 0.025 u µl^−1^ of GoTaq DNA polymerase (Promega) in 1× reaction buffer with 2 mM of MgCl_2_ (95°C for 5 min; 40 cycles of 95°C 45 s, 60°C 45 s, 70°C 45 s; 70°C 10 min) (see Figure S1 for primer sequences and correspondence with the IMGT nomenclature of gene names based on available sequences, IMGT Repertoire, http://www.imgt.org). Specific IGHV and C primers amplify VHC sequences with a given IGHV but with different IGHJ content and diverse CDR3 lengths. In a second step, VHC PCR products were subjected to run-off reactions (PCR2) using 5′ 6-FAM- fluorescent C internal, isotype specific, reverse primers. Two µl of PCR1 product were used as template using 0.4 mM of each dNTP, 10 pmoles of the fluorescent reverse primer, and 0.025 u µl^−1^ of GoTaq DNA polymerase (Promega) in 1× reaction buffer with 2 mM of MgCl_2_ (95°C for 5 min; 5 cycles of 95°C 1 min, 60°C 1 min, 70°C 2 min; 70°C 10 min). Two µl of run-off product were mixed with 8 µl deionized formamide (Applied Biosystems) and 0.5 µl of the internal standard (GeneScan 500XL ROX, size standard, Applied Biosystems). Mix was denatured at 95°C for 5 min and placed on ice before analysis in an ABI 3730HT sequencer (Applied BioSystems) at GeT-PlaGe core facility, Toulouse, France. CDR3 length distributions were analyzed using GeneMapper (Applied BioSystems) and ISEApeaks software [30], [37] to extract and analyze spectratype data for each VHC combinations. Each spectratype or profile is composed of several peaks (typically 4 to 10 for VHCμ and VHCδ, and 5 to 13 for VHCτ) separated according to their corresponding length of run-off products, spaced by 3 nucleotides as expected for in-frame transcripts. Each peak corresponds to a given CDR3 length. For each profile associated with a VHC combination, the area under each peak was calculated and stored in a peak database. These values were then computed to quantify the differences between spectratypes. As the intensity of CDR3 peaks is not comparable between different profiles/amplifications, we considered the percentage of use of each CDR3 length (i.e. the “relative area”), obtained by dividing the area of CDR3 peaks by the total area of all peaks within the profile. In a given context, the “perturbation” for a given VHC profile was calculated as follows: 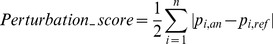
Where: **p_i,an_** and **p_i,ref_** are the relative areas of the peak **#i** from the analyzed and reference profiles respectively; n is the number of peaks detected in the reference profile. The reference profile is the average profile of individuals from a homogeneous group chosen as reference group. Perturbation scores are computed using the formula above for all VHC profiles and across all analyzed samples including the reference group. VHC perturbations range from 0 (identical profiles), to 100 (non-overlap profiles). To assess a significant perturbation between infected and control groups, we performed statistical tests at level α = 0.01 (type I error) on the perturbation index. Statistical significance of the difference in perturbation scores for two different groups was determined using empirical Bayes test from the limma packages (R/Bioconductor). We chose this test because it outperforms the classical Student t-test or Mann-Whitney-Wilcoxon non parametric test for small samples [67]. As recommended in [68] for a multiple testing problem (several tests done simultaneously), we used Benjamini-Hochberg procedure [69] to control the False Discovery Rate (FDR) which is the expected proportion of false positives among positive tests. Agglomerative Hierarchical clustering (Euclidean distance, K = 3) was performed using the software TM4 MultiExperiment Viewer (TMEV) with these data to group the different repertoires into three classes. The complete linkage method was chosen to aggregate similar clusters.

### Cloning of VHC and VHJ PCR products

VHC or VHJ PCR products were cloned into pCR2.1 vector (TOPO TA Cloning Kit, Invitrogen, San Diego, CA). The PCR product was concentrated and purified using the PCR purification and gel extraction kits (QIAgen) and 1.5 µl of the purified product was used in the cloning process, following manufacturer's instructions. Clones were picked at random, plasmidic DNA were purified (plasmid miniprep spin kit, Nucleospin, Macherey-Nagel, Durin, Germany) and sequenced using M13 universal primers.

### 454 GS FLX high-thoughput pyrosequencing technology

454 pyrosequencing applied on selected V_H_C combinations involved in major (VH4 and VH5.1 for IgM; VH4 for IgT), moderate (VH1.1 for IgM; VH5.1 for IgT) or weak (VH5.4 for IgM and IgT). PCR products amplified from the same individuals as for the spectratype analysis were analyzed, providing 1500 to 3000 useful sequences per PCR product. Sequencing libraries were produced from relevant PCR products mixed in equal amounts after picogreen quantification. To identify the origin of sequences, tagged PCR products were created using a nucleotide based barcode system (or “molecular identifier”, MID). IGH V, J or C MID-primers were designed by adding a MID sequence of 10 bp upstream to the primer sequence to analyse selected VHC or VHJ PCR products. The forward and the reverse primers of a given PCR product contained the same MID. Ten different MID sequences were used, allowing the co-processing of the same VHC or VHJ product from different individuals or samples. Primers used were: forward VH1.1, VH4, VH5.1, VH5.4, VH6, and reverse Cμ1, Cτ1, JH6. PCR conditions were as follows: 1 µl cDNA was used as template, using 0.4 mM each dNTP, 0.4 µM each MID-primer (forward: V-specific, reverse: J-specific or C-specific), and 0.08 u µl^−1^ of BIOTAQ DNA polymerase (Bioline) in 1× reaction buffer with 3 mM MgCl_2_ (95°C for 5 min; 40 cycles of 95°C 45 s, 60°C 45 s, 70°C 45 s; 70°C 10 min). MID-tagged PCR products were cleaned by gel extraction (QIAgen gel extraction kit), quantified, and aliquots of 100 ng of each PCR product were pooled for 454 library preparation. Library preparation and 454-pyrosequencing runs were done at GeT-PlaGe core facility, Toulouse Midi-Pyrénées, France, using GS FLX Titanium General DNA Library Preparation kit from Roche. Briefly, double stranded DNA was end-polished and ligated to sequencing adaptors. Library immobilization, fill-in reaction and single-stranded template DNA (sstDNA) library isolation were then performed following manufacturer's instructions. sstDNA library was then amplified and processed for sequencing using the GS FLX Titanium emulsion PCR protocol. Emulsion PCR reactions were prepared with a ratio of 0.12 molecule per DNA capture bead. 454 pyrosequencing libraries were constructed from around 20 pooled MID-labeled PCR products and sequenced using 1/8 of the chip which corresponds to a total of 70000 to 100000 reads.

A high proportion of unproductive, i.e. mainly out-of-frame, junctions (overall 40% in naïve animals) was observed. In fact, a defect in elimination of transcripts with STOP codons is well known in fish and has been repeatedly observed for TcRB transcripts of which more than 30% are unproductive due to out-of-frame V-D-J rearrangements. We verified that this high proportion of out-of-frame junction was observed among IG sequences as well, which were produced by Sanger sequencing of cloned VHC PCR products (unpublished data). Hence, this observation did not indicate a dramatic error rate of the pyrosequencing and we focused our analysis on productive rearrangements.

### Informatics pipeline and IMGT/HighV-QUEST sequence analysis

A script (described in Figure S4) was developed for sequence quality control, re-classification using barcodes, and preparation of sequences for annotation. Sequence annotation was performed by the IMGT/V-QUEST and IMGT/JunctionAnalysis programs of IMGT/HighV-QUEST [46], the IMGT web portal for high-throughput and deep IG and TR sequencing, ensuring a rigorous identification of V, D and J sequences, an exhaustive and detailed analysis of the junctions as well as filtering of unproductive junctions. For the time being, rainbow trout IGH loci have not been fully sequenced and annotated. Hence, our definition of V and J refers to subgroups rather than to defined genes/alleles. VH groups and J sequences mentioned in this study correspond to the current content of IMGT gene tables. CDR3 sequences are identified and extracted following the IMGT numbering. Thus, 1500 to 3000 useful sequences was generally obtained per PCR product. While this order of magnitude was far too small to produce a full (saturated) description of the junction repertoire associated to these VHC (or even VHJ) combinations, it provided a synthetic and cost-effective overview of the most prominent frequency changes induced by acute responses. Depending on sequencing run 45–61% of reads were checked for length, quality, presence of MID and V/C sequences, and sent to IMGT for annotation; 26–42% were finally considered. Based on the whole data set used in the project, on average 33% of reads were used in the final analysis.

Sequences were assigned to each analyzed sample from their primer-MID content. Briefly, primers and MID used in a run were identified in each 454 sequence. Sequences with a structure inconsistent with the primer-MID combinations used in the run were excluded. In the next step, sequences that did not contain complete junction (CDR3) region with flanking sequences long enough to identify the IGHV and IGHJ involved in the rearrangement, were discarded. The remaining sequences were then classified into sets corresponding to a unique combinations of [MID; V; C or J; sequencing direction] where the MID specified the original sample (tissue or individual fish).

To assess the error rate of 454 pyrosequencing, we chose a 140-bp region of the IGHV4 with no variation due to polymorphism among the reads, and we counted the indels and point substitutions in a dataset representing 1797091 nucleotides.

Sequences that passed these quality control criteria were then annotated using IMGT/HighV-QUEST (http://www.imgt.org/HighV-QUEST), which provided a full annotation including identification of the IGHV, IGHJ, IGHC, CDR3 length, location, and protein sequence. This annotation was then used for further specific analysis of the VH domain repertoire amplified in each sample. For each VHC or VHJ combination, the number of sequences having the same CDR3 amino acid sequence (i.e. its “number of occurrence”), and the number of distinct CDR3 found once, twice,… were determined. The physicochemical properties of the peptide encoded by the CDR3 were determined using ProtParam [70].

### Real-time PCR analysis of gene expression

For real time PCR, 3 µl of cDNA (1∶3 diluted) was used as a template for amplification using gene specific primers (Figure S1). PCR amplification was performed in a Mastercycler ep realplex (Eppendorf), using ready prepared 2× master mix (Power SYBR Green PCR master mix, Applied Biosystems) with a final PCR volume of 25 µl, in white 96-well plates (Eppendorf). PCR conditions were 95°C for 10 min followed by 95°C for 30 sec, 60°C for 30 sec and 72°C for 30 sec. The fluorescence signal output was measured and recorded at 80°C during each cycle for all wells for 40 cycles. A negative control (no template) reaction was also performed for each primer pair. A sample from the serial dilution was run on a 2% agarose gel and stained with ethidium bromide and viewed under UV light to confirm a band of the correct size was amplified.

A melting curve for each PCR was determined by reading fluorescence every degree between 60°C and 95°C to ensure only a single product had been amplified. Trout elongation factor-1α (EF-1α) and β-actin were used for normalization of expression. The relative expression level of the genes was determined using the Pfaffl method [71]. Efficiency of the amplification was determined for each primer pair using serial 10 fold dilutions of pooled cDNA, performed in the same plate as the experimental samples. The efficiency was calculated as E = 10 ^(−1/s)^, where s is the slope generated from the serial dilutions, when Log dilution is plotted against ΔCT (threshold cycle number).

### Estimation of seric Ig concentration and virus neutralization assay

IgM and IgT concentration in fish serum was estimated as previously reported in [21]. VHSV neutralization assays were performed as described in [7]. For the inhibition of neutralization, an excess of anti-IgM mab (1.14) or anti-igT polyclonal antibody [21] was pre-incubated with the serum 3 hours at room temperature, and this mix was then used for virus neutralization. While only partial inhibition was observed at low dilutions of serum, inhibition reached a reproducible plateau for higher dilutions. Typically, maximum inhibition was observed when using 10 µl of serum diluted 100 times or more, with 5 µg of anti IgM and 1 µg of anti IgT antibodies. A mouse IgG1 was used as an isotype matched control, and did not inhibit virus neutralization.

## Supporting Information

Figure S1 **Primers and CDR3 length spectratyping.** (A) Primers used in this study for CDR3 length spectratyping, pyrosequencing and QPCR. (B) Expressed VH groups and subgroups in IgM, IgD and IgT rearrangements in the spleen of the naïve fish used to setup the CDR3-length spectratyping system (C) Maximum and minimum length of the run-off products for each spectratyping profile represented in Figure 1B.
(DOC)

Figure S2 **IgM, IgD and IgT repertoires of healthy fish: spectratypes and analyses.** (A) Spectratypes observed in healthy animals for all VH combined with Cμ, Cδ or Cτ. (B) Individual diversity score (adjusted Shannon index) for expressed VH and each isotype, calculated in reference to the average profile of the IgM, D or T distributions in all control animals. (C) Individual perturbation scores for all expressed VH and each isotype, calculated in reference to the average profile of the IgM, or IgD or IgT distributions in all control animals.
(DOC)

Figure S3 **IgM, IgD and IgT repertoires in infected fish.** (A) FACS analysis of IgM^+^ and IgT^+^ B cells. IgM^+^IgT^−^ and IgM^−^IgT^+^ represented 25–40% and 5–15% of rainbow trout spleen leukocytes, respectively. No significant difference was observed in infected fish. (B) Complementary spectratypes observed in infected animals compared to control for different VH combined with Cμ, Cδ or Cτ.
(PDF)

Figure S4 **Detailed description of the method used for 454 data filtering, analysis and annotation.**
(DOCX)

Figure S5 **Error analysis and analyses performed on corrected JST datasets.** (A) Error analysis and production of corrected sequence datasets. (B) Normalized distributions of JST observed k times in the corrected versus unprocessed sequence datasets from control and virus infected fish. Distributions are represented for VH4, VH5.1 and VH5.4. (C) Kolmogorov-Smirnov tests on junction type distributions from corrected datasets of 454 pyrosequencing of spleen VHCμ and VHCτ expressed rearrangements.
(DOCX)

Figure S6 **Comparison of CDR3 length profiles from Immunoscope spectratyping and 454 pyrosequencing.** The CDR3 length profiles were computed from 454 sequence data and compared to the immunoscope spectratypes from the same fish. (A) shows that the distributions were quasi-identical, thus providing an independent validation of the repertoire description by 454 sequencing. Additionally, when a VHJμ PCR product was cloned, and 160 clones sequenced at random, the junction frequencies were consistent with two 454 independent sets of sequences (B). We therefore conclude that 454 pyrosequencing produces a representation of the junction repertoire that is in good accordance with widely used former technologies.
(DOCX)

Figure S7 **Complementary data from 454 pyrosequencing.** (A) JH usage in VH4Cμ expressed rearrangements. (B) Normalized distributions of JST observed n times in the sequence datasets from control and virus infected fish are represented for VH5.4 Cμ (C) Normalized distributions of JST observed n times in the sequence datasets from control and virus infected fish are represented for VH5.4 Cτ.
(PDF)

Figure S8 **Kolmogorov-Smirnov (KS) statistics on junction sequence type (JST) distributions.** (A) Procedure for aggregation of data from different fish to compute KS tests. (B) KS tests on JST distributions from unprocessed 454 pyrosequencing datasets of spleen VHCτ expressed rearrangements.
(DOCX)

Figure S9 **Sampling model of the IgHμ and IgHτ 454 pyrosequencing.** Does a given JST represent an activated, amplified versus a resting, non amplified B cell clone? Do abundant JST reflect non amplified B cell clones? Consequences for the identification of public and private responses.
(PDF)

Figure S10 **Pajek representations of IgM and IgT repertoires.**
(PDF)

Figure S11 **JST encoded by different nucleotide sequences.**
(PDF)

Figure S12 **Impact of virus infection on the expression of τ1, τ2, τ3 subtypes, δ isotype and transcription factors blimp1 and Pax5.** Relative expression of the constant region of the different Ig isotypes in VHSv infected and in control fish. Results were normalized to EF1α and β-actin and relative expression was calculated using the Pfaffl method. Each value is the fold increase as an average of the results of comparison of one infected fish with the results of 3 control fish. No amplification of the τ*2* subtype was found our clonal fish. Control values are set at 1. * indicates significant increase with respect to controls (p<0.05).
(PDF)

Figure S13 **Average sequence composition and biochemical properties of VHJμ and VHJτ JST in control and infected fish.** VHJμ and VHJτ JST composition and biochemical properties are modified by infection but do not show drastic differences between isotypes.
(DOCX)

Figure S14 **Estimation of the Ab producing cell frequency from clonal expansions of junctions detected in corrected and unprocessed datasets.** (A) Estimation of Ab-producing cell (AbPC) frequency. (B) Calculations with unprocessed data. (C) Comparison of frequencies estimated from unprocessed and corrected datasets.
(DOCX)
