# Supplementary material for: Teleost Fish Mount Complex Clonal IgM and IgT Responses in Spleen upon Systemic Viral Infection
Source: PLoS Pathog. 2013 Jan 10;9(1):e1003098. doi: 10.1371/journal.ppat.1003098 (PMC3542120; doi:10.1371/journal.ppat.1003098)
Supplement: Figure S1 — Primers and CDR3 length spectratyping. (A) Primers used in this study for CDR3 length spectratyping, pyrosequencing and QPCR. (B) Expressed VH groups and subgroups in IgM, IgD and IgT rearrangements in the spleen of the naïve fish used to setup the CDR3-length spectratyping system (C) Maximum and minimum length of the run-off products for each spectratyping profile represented in Figure 1B. (DOC) [file ppat.1003098.s001.doc]

**Figure S1**

**A.** Primer sequences used in this study.

| Primer  Name | Primer  Sequence (5’-3’) | IMGT name  of Ig V genes | |  | | |
| --- | --- | --- | --- | --- | --- | --- |
| *Repertoire* |  |  |  | | | |
| VH1.1-F | AGCAGTGATGGTGGCAGCACT | IGHV1S1 | | | | |
| VH1.2-F | CAATTAGTGATTCAAGCAGTTATA | IGHV1S2 |  | | | |
| VH1.3-F | CAGCGCATTATGACATTAGAAATA | IGHV1S3 |  | | | |
| VH1.4-F | TTCTGCACCAAGTGGAGCTGACAA | IGHV1S5 |  | | | |
| VH1.5-F | TTATAAATATGATAGTGCTGAAATC | IGHV1S4; IGHV1S6; IGHV1S7 |  | | | |
| VH2-F | GGTCTGAGAGCAGAGGACTCTGC | IGHV2S1 to IGHV2S3 |  | | | |
| VH3-F | TTGGAAACATGTGTGGCAGT | IGHV3S1 to IGHV3S4 |  | | | |
| VH4-F^a^ | ACTCTGGTTCAACAGATGCTCCAGTC | IGHV4S1 |  | | | |
| VH5.1-F | TACACACTGGTGGATCGAGTC | IGHV5S2; IGHV5S5; IGHV5S6; IGHV5S7; IGHV5S8; IGHV5S9 |  | | | |
| VH5.2-F | CAGCACAGCTAGTACACCCAT | IGHV5S3 |  | | | |
| VH5.3-F | CTATAGTTATAGTACTACTA | IGHV5S4 |  | | | |
| VH5.4-F | ATATTAGCACACAGAGTAATCC | IGHV5S1 |  | | | |
| VH6-F^a^ | CTGAAAAA(C/T)AAGTTCAGCCTC | IGHV6S1 to IGHV6S9 |  | | | |
| VH7-F | GTTCAGCATTTCAACACATGC | IGHV7S1 |  | | | |
| VH8-F | AAGGACAGCA(G/C)(A/T)AATTTCTATCTG | IGHV8S1 to IGHV8S11 |  | | | |
| VH9-F | AAAGACACCTCCAAAAACCAGC | IGHV9S1 to IGHV9S7 |  | | | |
| VH10-F | AGCAAGATCACTTCTAGATATG | IGHV10S1 |  | | | |
| VH11-F | CATCTGGAGTGGTGGATCAATTG | IGHV11S1 |  | | | |
| VH12-F | ggaccatctattatgatggaagc | IGHV12S1 |  | | | |
| VH13-F | gatacctgtgtagtagtagtagc | IGHV13S1 |  | | | |
| JH1-R^a^ | GCTGAGACGGTGACTTGTGT |  |  | | | |
| JH2-R^a^ | GATGATACTGTGATCAATGT |  |  | | | |
| JH3-R^a^ | AGAAGTTATCGTGACTTGGGA |  |  | | | |
| JH6-R^a^ | GATGAAACAGTAACCATTGT |  |  | | | |
| JH7-R^a^ | GAAACGGTTACTATTGTAC |  |  | | | |
| Cμ2-R | AGAGACGGCTGCTGCAGATATTCC |  |  | | | |
| Cμ1-R* (internal) | CACATTGCGCAAGAGGGAACAA |  |  | | | |
| Cδ-R | TGGACTGGAGATGTGGTCAC |  |  | | | |
| Cτ2-R | GATGTCGTTAGAAGGGGTTCCA |  |  | | | |
| Cτ1-R* (internal) | GTTCCACAGTTCATAAGAGTG |  |  | | | |
| *qPCR* |  |  |  | | | |
| EF1-α-qF | CAAGGATATCCGTCGTGGCA |  |  | | | |
| EF1-α-qR | ACAGCGAAACGACCAAGAGG |  |  | | | |
| β-actin-qF | GGGAGAAGATGACCCAGATCATG |  |  | | | |
| β-actin-qR | GGTGGTACGGCCAGAGGC |  |  | | | |
| IgHCT1-qF | ATCATCCTGGAGGTGCGAAGA |  |  | | | |
| IgHCT1-qR | GACTGTCTCGGAGGTGAAATCT |  |  | | | |
| IgHCT2-qF | ACGGAAATAACGAGATGAAAAACA |  |  | | | |
| IgHCT2-qR | GAACGGACACTGAAGGGGCT |  |  | | | |
| IgHCT3-qF | GGCGAACCCACCACCGAGA |  |  | | | |
| IgHCT3-qR | CCATCCACCTCATCCAGTCCA |  |  | | | |
| IgHCM-F | CCTTAACCAGCCGAAAGGG |  |  | | | |
| IgHCMmb-R | CCAACGCCATACAGCAGAG |  |  | | | |
| IgHCMsec-R | TGAGGTTCTATCAATGGTTCTC |  |  | | | |
| OnmyIgTC4-F | GTGACGTCTACATCATGTGGAAGG |  |  | | | |
| OnmyIgTmb-R | AAGCCCGCCTCAGGCTCAGTC |  |  | | | |
| OnmyIgTsec-R | ATCACTTGTCTTCACATGAGTTA |  |  | | | |
| IgHCD-F | CGGGTCATTCCTCCAAATATC |  |  | | | |
| IgHCD-R | GCTTCTGCTGGACTGGAGAT |  |  | | | |
| Blimp-1-F | AGCTGTCCAACCTCAAGGTCC |  |  | | | |
| Blimp-1-R | TTGCGGCACACCTGGGCATTC |  |  | | | |
| Pax5-F | ACGGAGATCGGATGTTCCTCTG |  |  | | | |
| Pax5-R | GATGCCGCGCTGTAGTAGTAC |  |  | | | |
| *Junction-specific amplifications*  VH5.1junc1 gcccggta(t/c)aa(t/c)aa(t/c)aatgct  VH4jun2 cgagaggggggcgccactcccg  *High throughput sequencing* | |  | | |  |  |
| MID-1 | ACGAGTGCGT^b^ |  |  | | | |
| MID-2 | ACGCTCGACA^b^ |  |  | | | |
| MID-3 | AGACGCACTC^b^ |  |  | | | |
| MID-4 | AGCACTGTAG^b^ |  |  | | | |
| MID-5 | ATCAGACACG^b^ |  |  | | | |
| MID-6 | ATATCGCGAG^b^ |  |  | | | |
| MID-7 | CGTGTCTCTA^b^ |  |  | | | |
| MID-8 | CTCGCGTGTC^b^ |  |  | | | |
| MID-10 | TCTCTATGCG^b^ |  |  | | | |
| MID-11 | TGATACGTCT^b^ |  |  | | | |

* The 5’ 6-FAM- fluorescent version of the primer was used for Immunoscope analysis. ^a^ Also used for qPCR analysis. ^b^ Followed by the corresponding repertoire primer sequence.

**B. Expressed VH groups and subgroups in IgM, IgD and IgT rearrangements in the spleen of the naïve fish used to setup the CDR3-length spectratyping system.**

|  | VH1.1 | VH1.2 | VH1.3 | VH1.4 | VH1.5 | VH2 | VH3 | VH4 | VH5.1 | VH5.2 | VH5.3 | VH5.4 | VH6 | VH7 | VH8 | VH9 | VH10 | VH11 |
| --- | --- | --- | --- | --- | --- | --- | --- | --- | --- | --- | --- | --- | --- | --- | --- | --- | --- | --- |
| IgM | + |  |  |  |  |  | +/- | + | + | + |  | + | + |  | + | + |  |  |
| IgD | + |  |  |  |  |  | +/- | +/- | +/- | +/- |  | + | + |  | + | + |  |  |
| IgT | +/- |  |  |  | +/- |  | +/- | + | + | + |  | + | +/- |  | + | + |  |  |

|  | VH12 | VH13 |
| --- | --- | --- |
| IgM | - | - |
| IgD | - | - |
| IgT | - | - |

**C. Max and Min length of the run-off products for each spectratyping profile represented in Figure 1B.**

|  |  |  |  |
| --- | --- | --- | --- |
| **VHCμ/δ** | from | to | theoretical lenght |
| VH1.1 | 233 | 257 | 244 |
| VH3 | 240 | 264 | 252 |
| VH4 | 237 | 261 | 246 |
| VH5.1 | 234 | 258 | 246 |
| VH5.2 | 233 | 257 | 245 |
| VH5.4 | 237 | 261 | 249 |
| VH6 | 258 | 282 | 265 |
| VH8 | 169 | 193 | 178 |
| VH9 | 170 | 194 | 185 |
|  |  |  |  |
|  | 10 peaks |  |  |
|  |  |  |  |
| **VHCτ** | from | to | theoretical lenght |
| VH1.5 | 241 | 274 | 254 |
| VH3 | 246 | 273 | 258 |
| VH4 | 245 | 272 | 252 |
| VH5.1 | 240 | 267 | 252 |
| VH5.4 | 245 | 272 | 255 |
| VH6 | 265 | 286 | 271 |
| VH8 | 172 | 199 | 184 |
| VH9 | 176 | 203 | 191 |
|  |  |  |  |
|  |  |  |  |
