# Supplementary material for: Teleost Fish Mount Complex Clonal IgM and IgT Responses in Spleen upon Systemic Viral Infection
Source: PLoS Pathog. 2013 Jan 10;9(1):e1003098. doi: 10.1371/journal.ppat.1003098 (PMC3542120; doi:10.1371/journal.ppat.1003098)
Supplement: Figure S2 — IgM, IgD and IgT repertoires of healthy fish: spectratypes and analyses. (A) Spectratypes observed in healthy animals for all VH combined with Cμ, Cδ or Cτ. (B) Individual diversity score (adjusted Shannon index) for expressed VH and each isotype, calculated in reference to the average profile of the IgM, D or T distributions in all control animals. (C) Individual perturbation scores for all expressed VH and each isotype, calculated in reference to the average profile of the IgM, or IgD or IgT distributions in all control animals. (DOC) [file ppat.1003098.s002.doc]

**Figure S2. IgM, IgD and IgT repertoires of healthy fish.**

**A. Spectratypes observed in healthy animals for all VH combined with Cμ, Cδ or Cτ**


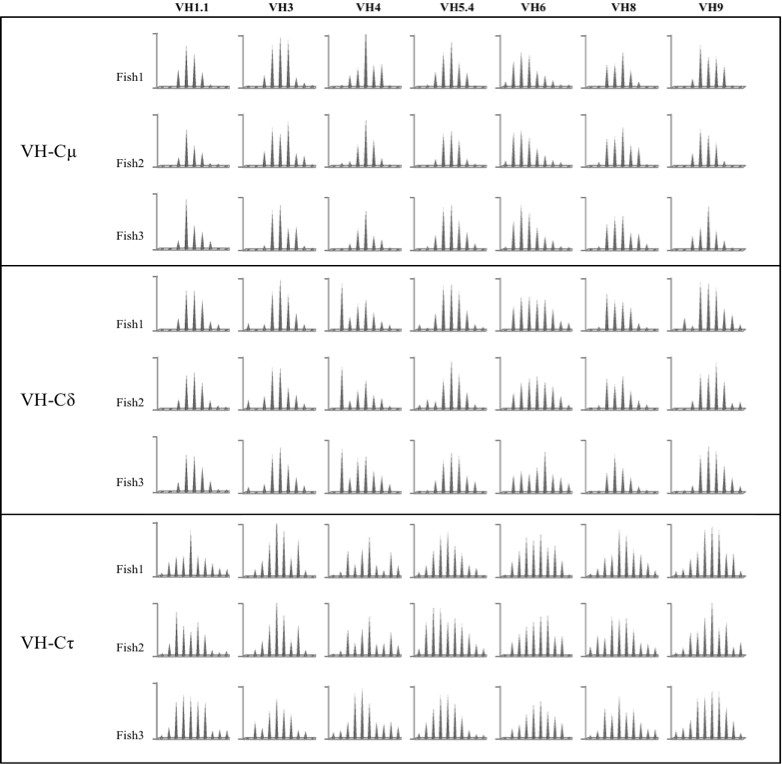


**B. Individual diversity score (adjusted Shannon index) for expressed VH and each isotype, calculated in reference to the average profile of the IgM, D or T distributions in all control animals.**

An intense red colour indicates a high value of the score, i.e. a high diversity of the profile (it is therefore maximal in diverse profiles from control fish). In fact, the Shannon index is increased either by having additional peaks, or by having a greater peak surface evenness. Like standard diversity indices, Shannon entropy therefore takes its maximal value when the distribution of observed species is uniform, whereas real maximal Ig repertoire diversity is expected for a Gaussian-like bell-shaped distribution of CDR3 lengths. We therefore computed a modified version of Shannon index, which reaches its maximum for a Gaussian CDR3 length distribution (see below).

| 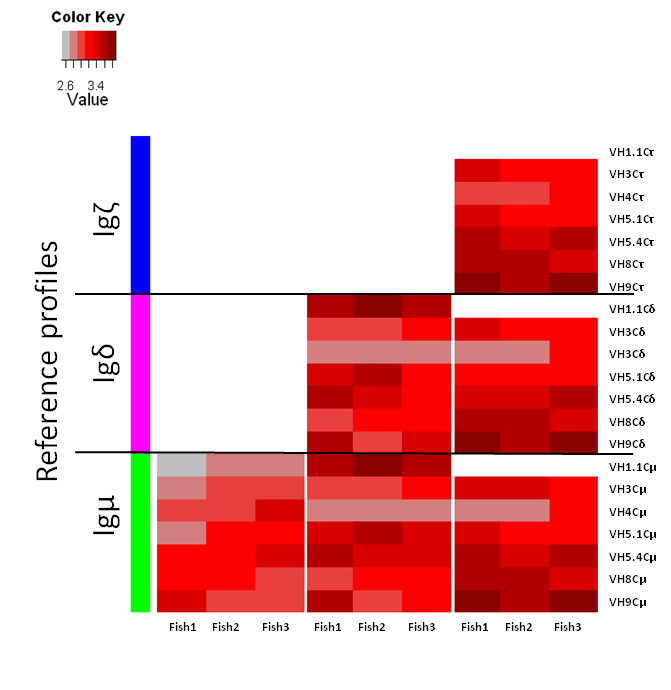  IgM IgD IgT |
| --- |

*Computation of modified shannon index for repertoire diversity:*

The repertoire diversity can be assessed by diversity measures based on the concept of Shannon entropy that provides a measure of the quantity of information encompassed in the repertoire. Given P = (p1,…,ps): a CDR3 profile where pi is the relative proportion of the peak #i within S peaks and $\sum_{i=1}^{S} p_{i}=1$.The Shannon diversity index could be estimated using the following formula:

$$H=-\sum_{i=1}^{S} p_{i}\log_{i}(p_{i})$$

H reaches the maximum when pi (i=1,..,S) follow a uniform distribution, whereas maximal TCR repertoire diversity is expected for a Gaussian-like distribution of CDR3 lengths. We therefore computed a modified version of Shannon entropy which reaches its maximum for a Gaussian-like CDR3 length profiles by transforming profile P into profile Q* according to a reference profile Q = (q1,…,qS) (considered as having the maximal diversity) and applied Shannon entropy to the transformed profile Q*.

First we calculated Profile Q’ (q’1,…,q’S):

$q_{i}^{'}=\frac{q_{i}-p_{i}}{1-p_{i}}$ if $p_{i}\leq q_{i}$

$q_{i}^{'}=\frac{q_{i}}{S\times p_{i}}$ if $0\leq q_{i}\leq p_{i}$

To satisfy the normalization hypothesis of Shannon entropy, we normalized profile Q’ by dividing each relative proportion by its overall sum thus obtaining Q*:

$$q_{i}^{*}=\frac{q_{i}^{'}}{\sum_{i=1}^{S} q_{i}^{'}}$$

Finally, the Shannon entropy for the transformed and normalized profile Q* was computed using:

$$H=-\sum_{i=1}^{S} q_{i}^{*}\log_{2}(q_{i}^{*})$$

**C. Individual perturbation scores for all expressed VH and each isotype, calculated in reference to the average profile of the IgM, or IgD or IgT distributions in all control animals.**

| 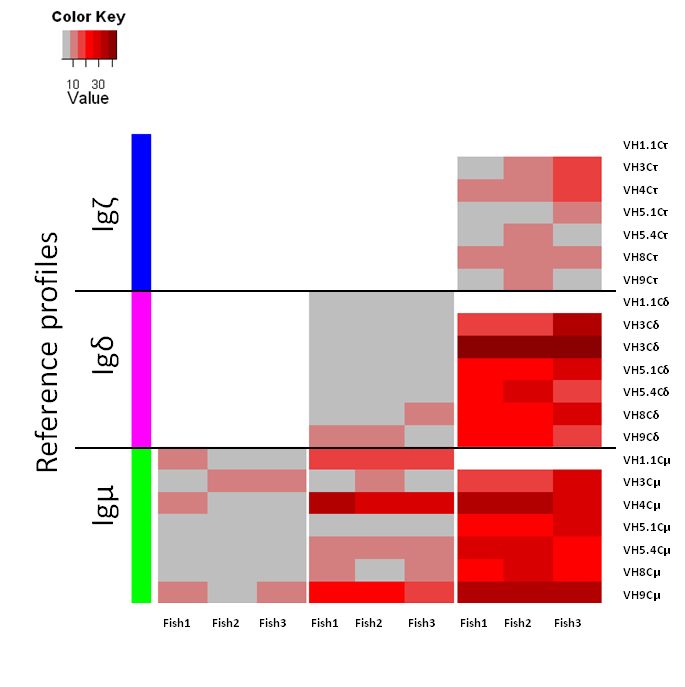 |
| --- |
| IgM IgD IgT |
