# Supplementary material for: Teleost Fish Mount Complex Clonal IgM and IgT Responses in Spleen upon Systemic Viral Infection
Source: PLoS Pathog. 2013 Jan 10;9(1):e1003098. doi: 10.1371/journal.ppat.1003098 (PMC3542120; doi:10.1371/journal.ppat.1003098)
Supplement: Figure S3 — IgM, IgD and IgT repertoires in infected fish. (A) FACS analysis of IgM+ and IgT+ B cells. IgM+IgT− and IgM−IgT+ represented 25–40% and 5–15% of rainbow trout spleen leukocytes, respectively. No significant difference was observed in infected fish. (B) Complementary spectratypes observed in infected animals compared to control for different VH combined with Cμ, Cδ or Cτ. (PDF) [file ppat.1003098.s003.pdf]

Figure S3. Impact of VHSV infection on IgM, IgD and IgT repertoires.

A. FACS analysis of IgM+ and IgT+ B cells. IgM+IgT- and IgM-IgT+ represented 25-40% and 5-15% of rainbow trout spleen leukocytes, respectively. No significant difference was observed in infected fish.

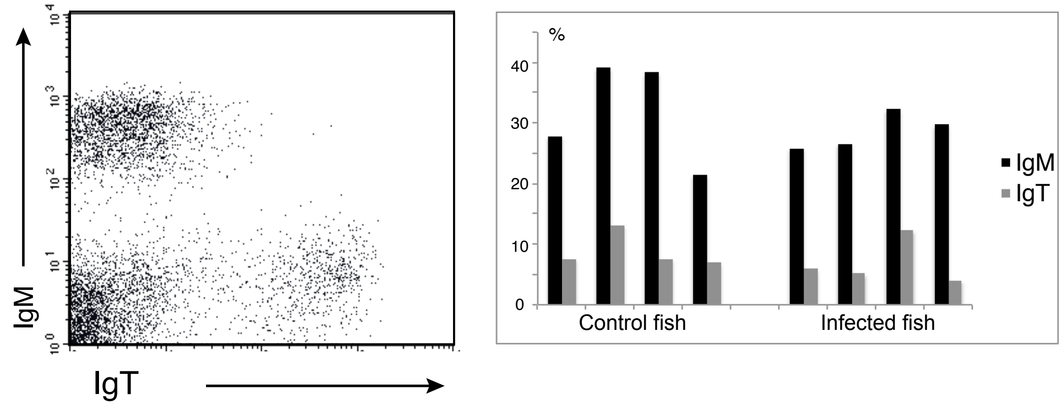

B. Complementary spectratypes observed in infected animals compared to controls for different VH combined with Cμ, Cδ or Cτ.

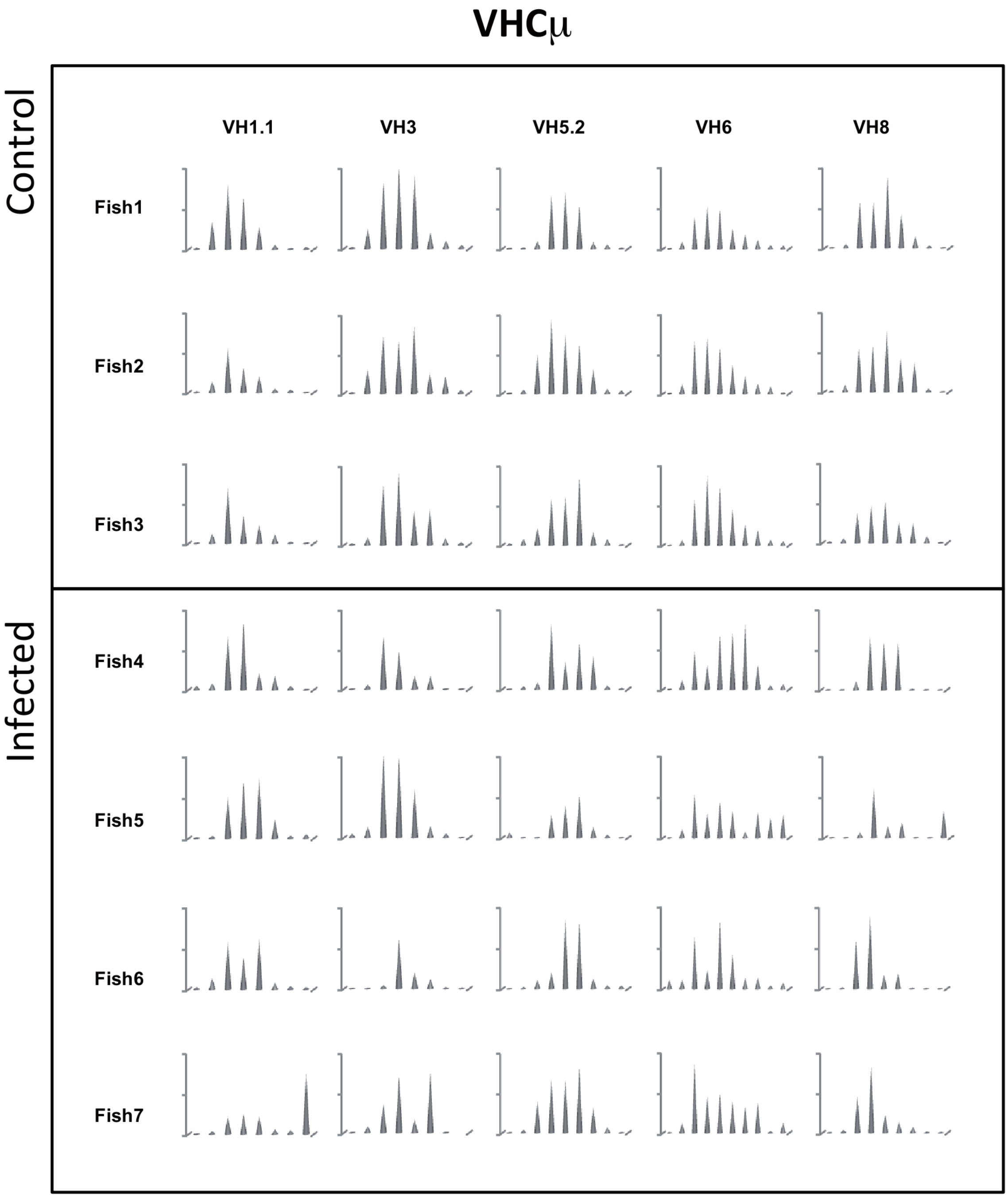

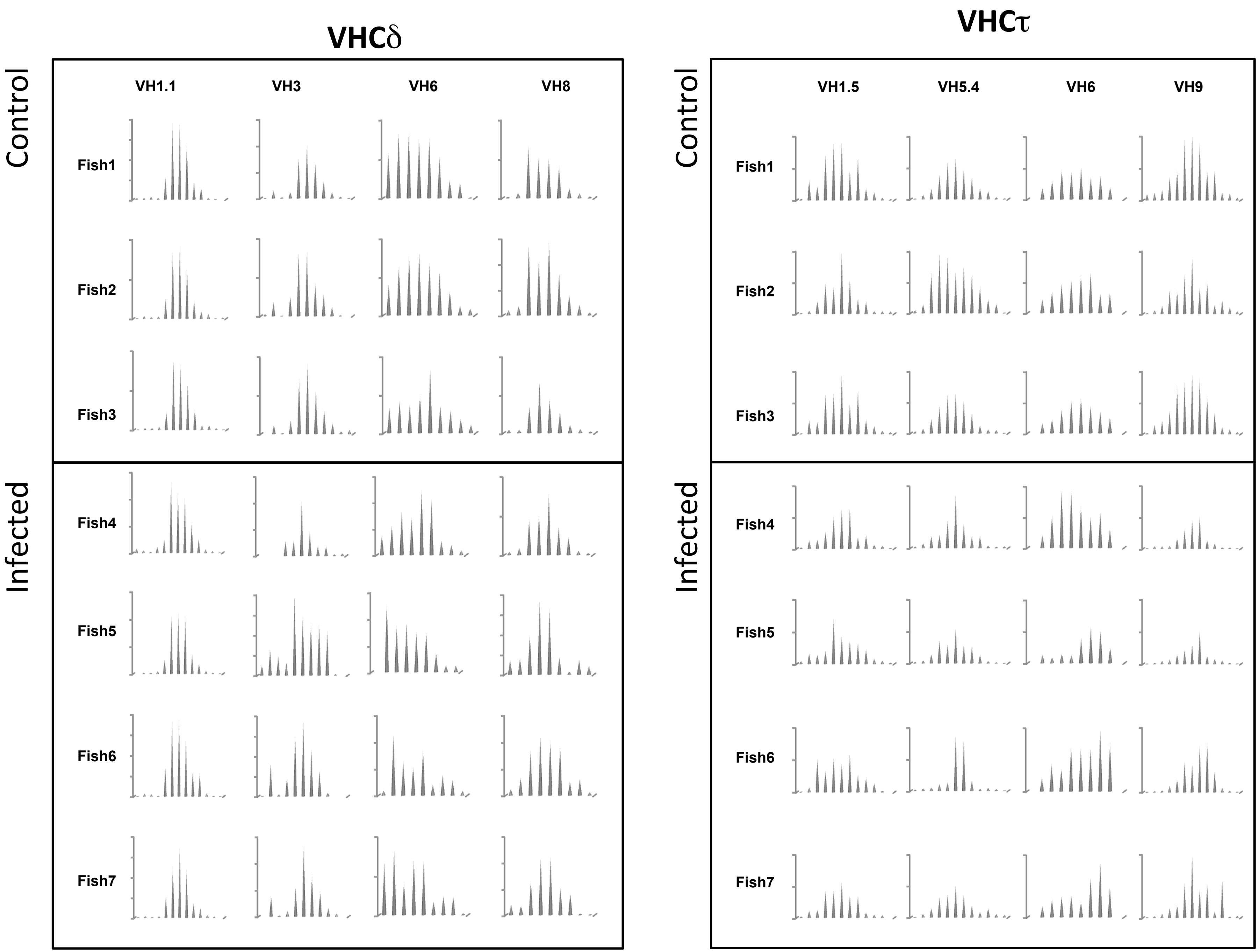
VHCδ
VHCτ
Control
Infected
VH1.1
VH3
VH6
VH8
VH1.5
VH5.4
VH6
VH9
Fish1
Fish2
Fish3
Fish4
Fish5
Fish6
Fish7
