# Supplementary material for: Teleost Fish Mount Complex Clonal IgM and IgT Responses in Spleen upon Systemic Viral Infection
Source: PLoS Pathog. 2013 Jan 10;9(1):e1003098. doi: 10.1371/journal.ppat.1003098 (PMC3542120; doi:10.1371/journal.ppat.1003098)
Supplement: Figure S4 — Detailed description of the method used for 454 data filtering, analysis and annotation. (DOCX) [file ppat.1003098.s004.docx]

**Figure S4.**

**Detailed description of the method used for 454 data filtering, analysis and annotation.**

## Step 0: Quality control – Sequence length distribution

Sequences produced by 454 are parsed and their length is computed and printed in a separated file.

A R^[[1]](#footnote-2)^ script uses these lengths to produce an image showing distribution of length values, which provides a rough idea of the run quality.

## Step 1: Sequence (de)composition

A file containing MID and primer sequences is created. Primers can be specific for V (forward primers) or C or J (reverse primers). The elements of this list are hereafter called “tags”.

(…)

>MID-**x**

ACGAGTGCGT

(…)

>VH4

ACTCTGGTTCAACAGATGCTCCAGTC

(…)

**Important convention: “the sequences of J and C primers are given in this file as the corresponding stretch on the 5’->3’ (coding sequence) of the cDNA, i.e. as the RC of the sequence of the primer itself”**

(**x** takes different values 1,2,3, …)

For each 454 tags, we search, from the 5’ end of the sequence towards 3’, for similarities with each of these sequences, using JAligner java library^[[2]](#footnote-3)^, either in forward or reverse direction. We decide that a similarity is relevant if the score returned by SmithWatermanGotoh.align method, divided by the tag sequence length is greater than 0.75. This threshold has been set up empirically to allow one or two substitutions but no more.

If a similarity is found, we report it in output file, together with the orientation and position of the hit on the sequence:

#Sequence ID #Length #Similarities

WFSY8M05GE54O 87 MID-4 _F;1 VH5.1_F;11

GWFSY8M05GMWW6 171

GWFSY8M05GF4V6 44 MID-4 _F;1 Chtaucom_R;11

GWFSY8M05GFLXJ 133 MID-4_F;1 VH5.1_F;11

GWFSY8M05GG3NT 65 MID-8_F;1 Chm_F;35 MID-8_R;57

## Step 2: Sequence selection

In each sequence composition, we look for the most right (i. e. 3’ side) tag - with V in forward orientation, and C or J in reverse orientation - found in the first 75 nucleotides. This value has been set up empirically to take into account multiple barcode/primer integration in the beginning of the sequence.

If a similar stretch is found, this hit will be hereafter called “anchor similarity”.

* If no anchor similarity is found, sequence is discarded.

* Otherwise,

if this anchor similarity is preceded by a MID, we keep the sequence from the start of the MID similarity, up to the end of the sequence^[[3]](#footnote-4)^.

If no MID similarity can be found, we keep sequence from the start of anchor similarity up to the end of the sequence.

## Step 3: Sequence dispatch

### SubStep 3.1: Re-computation of sequence length and similarity coordinates

As the sequences selected in the previous step have been trimmed, we compute again sequence length and coordinates of the start of each hit identified in the sequence decomposition in step 1.f

### SubStep 3.2: Constitution of set of sequences

Sequences beginning with composition {MID ; V} are put in a file named **set_BC_primerV.txt.^[[4]](#footnote-5)^**

Sequences beginning with composition {MID ; C} are put in a file named **set_BC_primerC.txt.**

Sequences beginning with composition {MID ; J} are put in a file named **set_BC_primerJ.txt.**

Sequences beginning with a V similarity are put in a file named **set_primerV.txt.**

Sequences beginning with a C similarity are put in a file named **set_primerC.txt.**

Sequences beginning with a J similarity are put in a file named **set_primerJ.txt.**

Other sequences are sent to a junk file named **set_others.txt**.

### SubStep 3.2: Constitution of set of sequences

A file containing tag sequences corresponding to start and end of V, C or J parts (both in forward and reverse orientation) is created. For a given V (or C or J), several “variants” of start and end tags of sequences may be specified, to take into account the co-amplification of several members of each V family by a given primer (see below VH6b_end1, VH6b_end2, VH6b_end 3):

#VH6b

>VH6b_start_F

CTGAAAAANAAGTTCAGCCTC

>VH6b_start_R

GAGGCTGAACTTNTTTTTCAG

>VH6b_end1_F

gtgtattattgtgct

>VH6b_end1_R

AGCACAATAATACAC

>VH6b_end2_F

gtgtattattgtgcc

>VH6b_end2_R

GGCACAATAATACAC

>VH6b_end3_F

gtttattattgtgct

>VH6b_end3_R

AGCACAATAATAAAC

In the following steps, we look for similarities of these tags with the sequences to be analyzed using JAligner java library as described in Step1 (see above, same parameters).

*This process aims at selecting the sequences with a complete V(D)J junction, testing for the presence of Vend* ***and*** *J or Cstart tags, as well as for the consistency of the V -respectively C- start and end tags.*

If a sequence is not discarded during this process, it is reported in a file dedicated to the barcode found in its sequence (de)composition.

Each file corresponding to a useful configuration (i.e. **set_BC_primerV.txt**, **set_BC_primerC.txt**, **set_BC_primerJ.txt**, **set_primerV.txt**, **set_primerC.txt**, **set_primerJ.txt**), will be split into different files, one file per MID (=per barcode), kept in folders respectively named set1, set2, … set6.

The file “**set_BC_primerV**“ will be thus split into a folder “**set1**” containing its sequences classified per MID (one file per MID).

We look for a V-part-end similarity (in forward direction) corresponding to the V detected using sequence primer in step1.

- If none is found, sequence is discarded.
- Otherwise, we look in sequence similarity composition if a C (or J) similarity is located just after the V.
  - If it is not the case, sequence is discarded.
  - Otherwise, we look for a C (or J)-part-start similarity corresponding to the same C (or J).
    - If none is found, sequence is discarded.
    - Otherwise, we look further in sequence similarity composition for a MID similarity.
      - If none is found, sequence is annotated with the coordinates of the V-part-end, and C (or J)-part-start and we specify that only one barcode has been found.
      - Otherwise, we check that the barcode at the beginning and the barcode detected after C (or J) part are identical (respectively in forward and reverse direction).
        - If it is not the case, sequence is discarded.
        - Otherwise, sequence is annotated with the coordinates of end of V-part-end, and C (or J)-part-start and we specify that two consistent barcodes have been found.

The file “**set_BC_primerC**“ will be split into a folder “**set2**” containing sequences classified per MID We look for a C start similarity (in reverse direction) corresponding to the same C detected using sequence primer in step1.

*If none is found, sequence is discarded.

*Otherwise, we look in sequence similarity composition if a V similarity is located just after the C.

- If it is not the case, sequence is discarded.
- Otherwise, we look for a V part end similarity corresponding to the same V (in the expected orientation).
  - If none is found, sequence is discarded.
  - Otherwise, we look further in sequence similarity composition for a MID similarity.
    - If none is found, sequence is annotated with the coordinates of V-part-end , and C-part-start and we specify that only one barcode has been found.
    - Otherwise, we check that the barcode at the beginning and the barcode detected after V-part are identical (respectively in forward and reverse direction).
      - If it is not the case, sequence is discarded.
      - Otherwise, sequence is annotated with the coordinates of V-part-end, and C-part- start , and we specify that two consistent barcodes have been found.

The file “**set_primerV**“ will be split into a folder “**set3**” containing sequences classified per MID.

We do the same as for **set_BC_primerV,** except that:

- If no barcode is detected after C (or J) part, sequence is discarded ^[[5]](#footnote-6)^
- If a barcode is detected after C (or J) part but in forward direction, sequence is discarded
- otherwise , sequence is annotated with the coordinates of V-part-end, and C (or J)-part-start and we specify that only one barcode has been found

The file “**set_primerC**“ will be split into a folder “**set4**” containing sequences classified per MID

We do the same as for **set_BC_primerC,** except that:

- If no barcode is detected after V part, sequence is discarded ^[[6]](#footnote-7)^
- If a barcode is detected after V part but in forward direction, sequence is discarded
- otherwise, sequence is annotated with the coordinates of V-part-end, and C-part-end and we specify that only one barcode has been found

The file “**set_BC_primerJ**“ will be split into a folder “**set5**” containing sequences classified per MID

We do the same as for **set_BC_primerC,** except that we look for a J part in place of C part.

The file “**set_primerJ**“ will be split into a folder “**set6**” containing sequences classified per MID We do the same as for **set_primerC,** except that we look for a J part in place of C part.

## Step 4: Classify sequences by unique combinations of {sequencing direction;MID;V;C or J}

For each MID:

We pool sequences collected in **set1** and **set3** (respectively corresponding to **set_BC_primerV** and **set_primerV**).

- Sequences are reported unchanged in a file (specific of a combination of {MID; V; C or J}) located in a directory named **sequence_forward**.

We pool sequences collected in **set2** and **set4** (respectively corresponding to **set_BC_primerC** and **set_primerC**). The sequences are converted into their reverse complementary sequences, and the coordinates of the Tag similarity regions are recomputed (according to the new orientation of the sequence)

- Sequences are reported unchanged^[[7]](#footnote-8)^ in a file (specific of a combination of {MID; V; C}) located in a directory named **sequence_reverse**.

We pool sequences collected in **set5** and **set6** (respectively corresponding to **set_BC_primerJ** and **set_primerJ**). The sequences are transformed into their reverse complementary sequences, and the coordinates of the tag hits are recomputed (according to the new orientation of the sequences)

- Sequences are reported unchanged^[[8]](#footnote-9)^ in a file (specific of a combination of {MID; V; J}) located in a directory named **sequence_reverse**.

## Step 5a: Prepare datasets of trimmed sequences for IMGT analysis

For each file created in the previous step(4), we select the junction with flanking regions long enough to allow IMGT identifying the V, J and C segments.

For forward sequences, we keep the region beginning at the position {V-part-start coordinate + 20 nucleotides}, up to the end of the sequence. (The length of 20 nt corresponds to the V primer region).

For reverse sequences, we keep the region from the beginning of the sequence, up to the 3’ end of the V-part-start tag (found in reverse direction).

Forward and reverse sequences are then collected in a unique file, specific of a unique {MID; V; C or J} combination.

## Step 5b: IMGT / HighV - QUEST analysis

Each file is submitted to the IMGT/ HighV-QUEST analysis webpage:
<http://www.imgt.org/HighV-QUEST>:


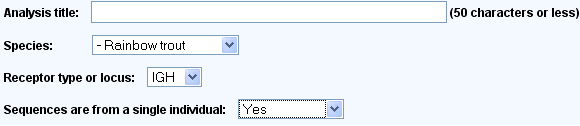


## Step 6: CDR3 and sequence occurrence distribution

Each one of IMGT/HighV-QUEST result file is parsed as follows:

We discard sequences that:

- are annotated “unproductive” or ”unknown” in **Functionality** field.
- have nothing reported in **J-REGION identity %** field and/or **J-GENE and allele** field

Sequences discarded because they do not contain a known J (or known V ) sequence are sent to a specific folder for further characterization of putative new J segments. ^[[9]](#footnote-10)^

For each combination of {V; J}, we count the number of sequences having the same CDR3 length (**CDR3-IMGT length** field). We produce a table with CDR3 length in columns, J segment in lines and specifying the number of corresponding sequences for each combination:

| ==================== | |  |  |  |  |  |  |  |  |  |  |  |  |  |  |
| --- | --- | --- | --- | --- | --- | --- | --- | --- | --- | --- | --- | --- | --- | --- | --- |
| VH4_Chm_MID-2F | |  |  |  |  |  |  |  |  |  |  |  |  |  |  |
| ==================== | |  |  |  |  |  |  |  |  |  |  |  |  |  |  |
|  |  |  |  |  |  |  |  |  |  |  |  |  |  |  |  |
| -------------------- | |  |  |  |  |  |  |  |  |  |  |  |  |  |  |
| IGHV4 |  |  |  |  |  |  |  |  |  |  |  |  |  |  |  |
| -------------------- | |  |  |  |  |  |  |  |  |  |  |  |  |  |  |
| Junction | 1 | 2 | 3 | 4 | 5 | 6 | 7 | 8 | 9 | 10 | 11 | 12 | 13 | 14 | 15 |
| IGHJ3*01 | 0 | 0 | 0 | 0 | 0 | 0 | 2 | 0 | 1 | 5 | 0 | 0 | 5 | 2 | 0 |
| IGHJ4*01 | 0 | 0 | 0 | 0 | 0 | 1 | 0 | 0 | 2 | 7 | 8 | 12 | 3 | 22 | 1 |
| IGHJ5*01 | 0 | 0 | 0 | 0 | 0 | 0 | 0 | 0 | 0 | 5 | 10 | 190 | 4 | 7 | 0 |
| IGHJ5*01, or IGHJ6*01 | 0 | 0 | 0 | 0 | 0 | 0 | 0 | 0 | 0 | 0 | 0 | 0 | 1 | 0 | 0 |
| IGHJ6*01 | 0 | 0 | 0 | 0 | 0 | 0 | 0 | 0 | 5 | 1 | 1 | 13 | 4 | 0 | 0 |
| IGHJ7*01 | 0 | 0 | 0 | 0 | 0 | 0 | 0 | 0 | 0 | 0 | 0 | 0 | 3 | 0 | 0 |
| all | 0 | 0 | 0 | 0 | 0 | 1 | 2 | 0 | 8 | 18 | 19 | 215 | 20 | 31 | 1 |

For each combination of {V ; J}, we count the number of sequences having the same amino acid sequence (**AA JUNCTION** field). We count how many times an amino acid sequence has been counted once, twice … (hereafter called count of occurrences).

We report a table with count of occurrences in column, J segment in lines and specifying the number of distinct CDR3 sequences found a given number of times (i.e. at a given count of occurrence):

| -------------------- |  |  |  |  |  |  |  |  |  |  |  |  |  |  |  |  |
| --- | --- | --- | --- | --- | --- | --- | --- | --- | --- | --- | --- | --- | --- | --- | --- | --- |
| IGHV4 |  |  |  |  |  |  |  |  |  |  |  |  |  |  |  |  |
| -------------------- |  |  |  |  |  |  |  |  |  |  |  |  |  |  |  |  |
| Junction | 1 | 2 | 3 | 4 | 5 | 6 | 7 | 8 | 9 | 10 | 11 | 12 | … | 200 | 201 | 202 |
| IGHJ1*01 | 136 | 23 | 13 | 14 | 11 | 5 | 10 | 3 | 4 | 3 | 3 | 2 | … | 0 | 0 | 0 |
| IGHJ2*01 | 66 | 11 | 6 | 7 | 2 | 1 | 3 | 0 | 1 | 1 | 2 | 1 | … | 0 | 0 | 1 |
| IGHJ4*01 | 4 | 0 | 0 | 0 | 0 | 0 | 0 | 0 | 0 | 0 | 0 | 0 | … | 0 | 0 | 0 |
| IGHJ4*01, or IGHJ5*01 | 1 | 0 | 0 | 0 | 0 | 0 | 0 | 0 | 0 | 0 | 0 | 0 | … | 0 | 0 | 0 |

1. <http://www.r-project.org> [↑](#footnote-ref-2)
2. JAligner (<http://jaligner.sourceforge.net/>) is a Java language implementation of Smith-Waterman algorithm with Gotoh's improvement for biological local pairwise sequence alignment using the affine gap penalty model.

   References :

   Smith TF, Waterman MS. [Identification of common molecular subsequences](http://www.ncbi.nlm.nih.gov/pubmed/7265238). J Mol Biol. 1981 Mar 25;147(1):195-7.

   Gotoh O. [An improved algorithm for matching biological sequences](http://www.ncbi.nlm.nih.gov/pubmed/7166760). J Mol Biol. 1982 Dec 15;162(3):705-8. [↑](#footnote-ref-3)
3. If anchor similarity concerns a J primer and previous to this anchor, there is a C similarity, we look if a MID similarity can be found before the C (composition of the form {MID; C; J}. This is relevant if VJ and VC PCR products have been sequenced in the same run. [↑](#footnote-ref-4)
4. **BC** stands for ‘Barcode’ i.e. MID ! [↑](#footnote-ref-5)
5. Different folders are created for discarded sequences: NoBC_TCR_StartV, NoBC_TCR_NoStartV, NoBC_NoTCR [↑](#footnote-ref-6)
6. Different folders are created for discarded sequences: NoBC_TCR, NoBC_NoTCR [↑](#footnote-ref-7)
7. Save conversion to reverse complementary sequences [↑](#footnote-ref-8)
8. Save conversion to reverse complementary sequences [↑](#footnote-ref-9)
9. If several possible V are indicated in the **V-GENE and allele** field, a warning is printed and the first one is taken into account by default. [↑](#footnote-ref-10)
