# Supplementary material for: Teleost Fish Mount Complex Clonal IgM and IgT Responses in Spleen upon Systemic Viral Infection
Source: PLoS Pathog. 2013 Jan 10;9(1):e1003098. doi: 10.1371/journal.ppat.1003098 (PMC3542120; doi:10.1371/journal.ppat.1003098)
Supplement: Figure S5 — Error analysis and analyses performed on corrected JST datasets. (A) Error analysis and production of corrected sequence datasets. (B) Normalized distributions of JST observed k times in the corrected versus unprocessed sequence datasets from control and virus infected fish. Distributions are represented for VH4, VH5.1 and VH5.4. (C) Kolmogorov-Smirnov tests on junction type distributions from corrected datasets of 454 pyrosequencing of spleen VHCμ and VHCτ expressed rearrangements. (DOCX) [file ppat.1003098.s005.docx]

**Figure S5. Error analysis and analyses performed on corrected JST datasets.**

1. **Error analysis and production of corrected sequence datasets**

We defined a JNS (for “junction nucleotide sequence”) as an *in-frame or out-of-frame CDR3 nucleotide sequence associated with a (V,J) pair*. Hence, a JNS comprises a number of reads with identical CDR3 sequence, a given V and a given J.

To assess the impact of technical errors due to PCR and 454 sequencing, we estimated for each JNS the respective contributions of sequences produced without error (“proper”), and of sequences coming from similar sequences through technical error (“exogenous “).

All JNS were put in a graph, where two JNS were linked if they differed at one and only one position. We neglected the events corresponding to double mutations. We also neglected the errors removing or producing in frame stop codons in JNSs.

*The following notations were defined:*

We took into consideration productive sequences annotated by IMGT (frame 0), as well as non productive sequences annotated by IMGT encoding junctions in frame +1 or -1, because such sequences can produce in frame JSN after 1 nucleotide insertion or deletion.

Each JNS “a” was noted JNS_a._

The number of reads of JNS_a_ was noted N_a_, and the length of the corresponding CDR3 was noted L_a._

In the graph JNS_a_ was connected to k JNS - noted JNS_b1 … bk_ in the graph – differing at one and only one position.

The exogenous part of reads of JNS_a_, noted Ex_a_ , was estimated by the contribution of the neighbors present in the graph. We are aware that this approximation is acceptable only because the error rate (order of magnitude 0.01) and length of JSN are low. Hence, only highly frequent JNS, which must be represented in the dataset, can have a substantial influence of their neighbors’ numbers of reads.

The average error rate per position is noted Er; it represents indels as well as substitutions.

Our estimation was a global rate of mutation per site (Er=3.10^3^). In the calculations hereafter, we considered that Er= 0.01, which was conservative and took into account the values previously reported for PCR and 454 sequencing procedure.

Each type of mutation was considered equally probable (3 possible substitutions, deletion, or insertion). The rate of one of these events was therefore estimated to be Er/5.

(1) Does a given JNS represent a real sequence, or just the result of errors from neighbor JNS?

As a first step, we assessed if a given JNS represented an actual sequence, or purely an artifact produced by errors from neighbor abundant JNS.

To this purpose, we estimated the exogenous part Ex_a_ of each JSN_a_ observed in the dataset.

$\left( 1 \right)$

where : JSNa is connected to k JNS (k=1….b) - noted JNS_b1 …..bk_ in the graph – that differ at one and only one

position, the number of reads of each JSN_b_ neighbor is noted N_b_, and Er is the rate of technical error per site.

* If $Ex\text{a}$ > N_a_observed_, we considered that JNS_a_ was entirely produced by error from neighbor JNS. Such JNS were eliminated.

* If not, we considered that JNS_a_ represented a real sequence.

1. How to assess the number of reads in each JNS, taking into account the error rate and the structure of the JNSs graph built from observed sequences?

As a second step, we tried to assess the real number of reads for each JNS; for this, we assessed the proper and the exogenous parts as well as the number of reads “lost” due to technical errors.

We estimated a corrected number of reads for a given JSN_a_, taking into account (1) reads “lost” by introduction of technical errors during the PCR plus sequencing process – such reads differs from JSN_a_ by one mutation, substitution or indel - and (2) reads “gained” mistakenly , i.e. the exogenous part Ex_a_ of JSN_a_ produced by technical error from neighbor sequences.

$N_{a}^{c}=N_{a\_observed}+N_{lost}-N_{gained}$ (2a)

Where : $N_{a}^{c}$ = corrected number of sequences in JNS_a_

N_a observed_= number of reads observed in JNS_a_

N lost= number of sequences lost from JNS_a_ by error

N gain= number of sequences gained by JNS_a_ by error from its neighbors b_1 …k_

Hence,

 (2b)

Where : $N_{a}^{c}$ = corrected number of sequences in JNS_a_

N_a_observed_= number of reads observed in JNS_a_

L_a_= length of JNS_a_

Er= rate of technical error per site

The number of reads of each JSN_b_ neighbor is noted N_b_

The corrected counts for JSN constituted corrected datasets, which were translated and aggregated into corrected JST datasets. The distribution of % of JST occurrence and KS tests on distribution similarity were then determined from these corrected datasets, as well as the estimation of Ab-producing and resting B-cell numbers (Figure S14C in the discussion).

**B. Normalized distributions of JST observed n times in the corrected versus unprocessed sequence datasets from control and virus infected fish.** Distributions are represented for VH4, VH5.1 and VH5.4. As in figures 2 and 4, the % of total JST number observed 1, 2 or 3 times is represented separately on the left of each histogram with a relevant scale.

| **VH4Cμ Control fish corrected data**  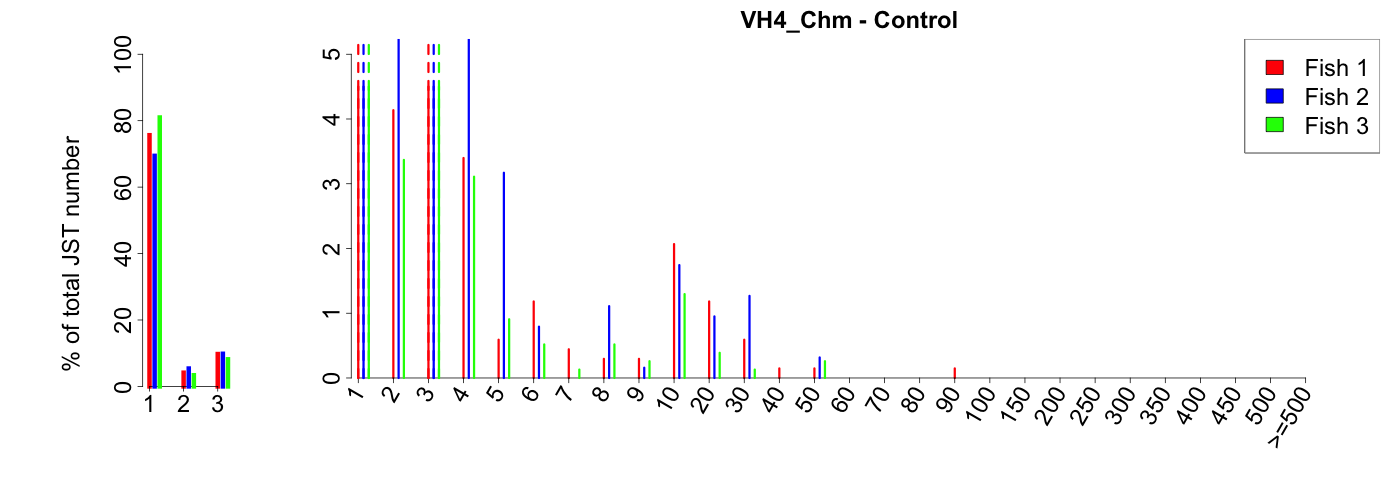 | **VH4Cμ Control fish unprocessed data**  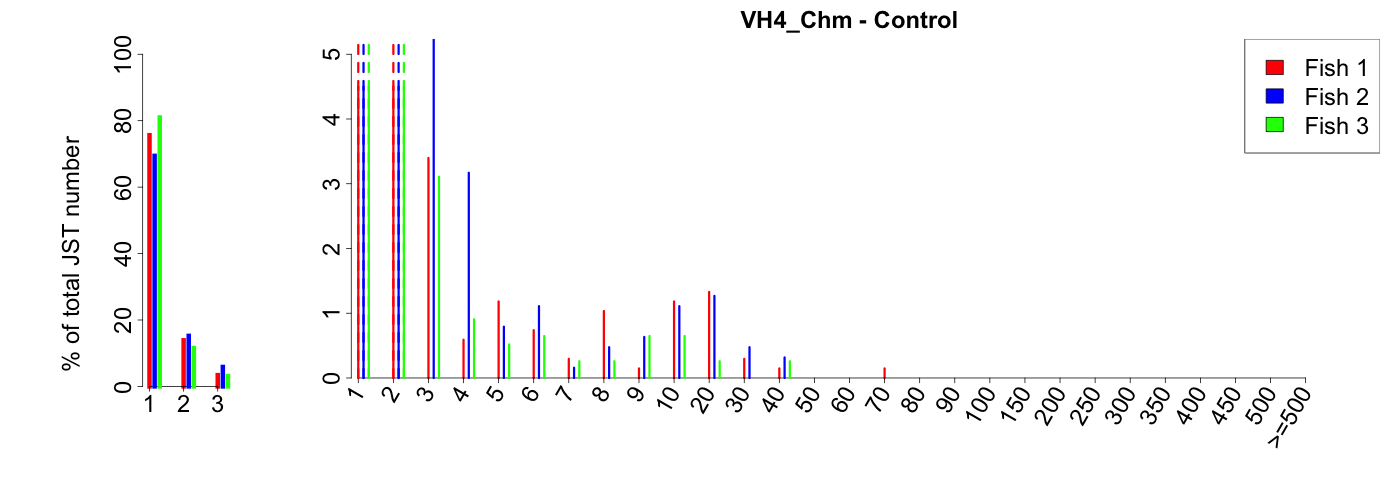 |
| --- | --- |
| **VH4Cμ Infected fish corrected data**  **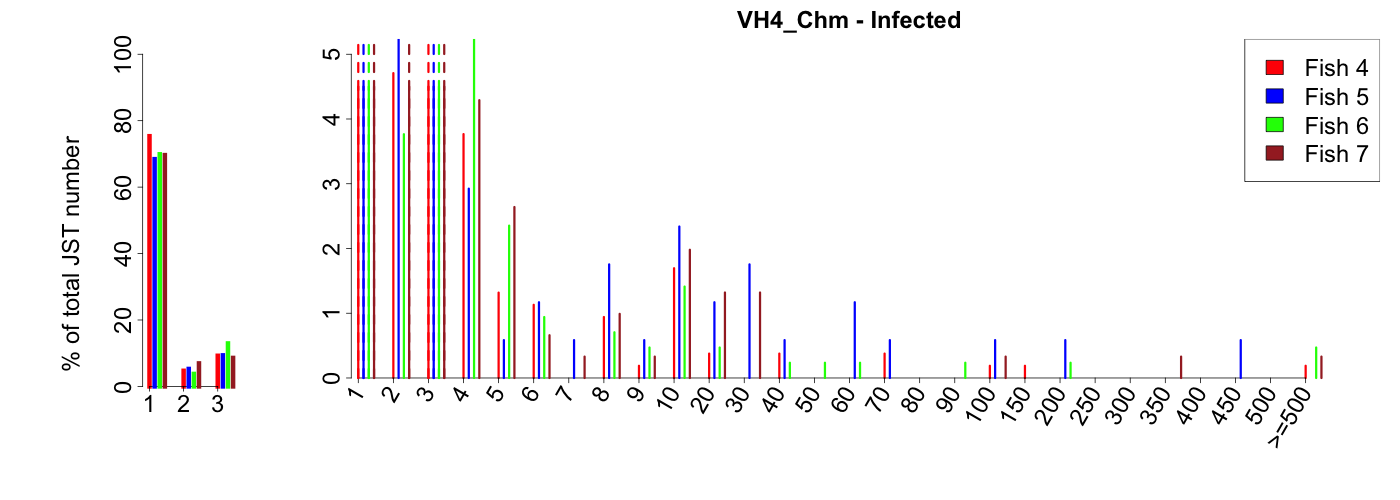** | **VH4Cμ Infected fish unprocessed data**  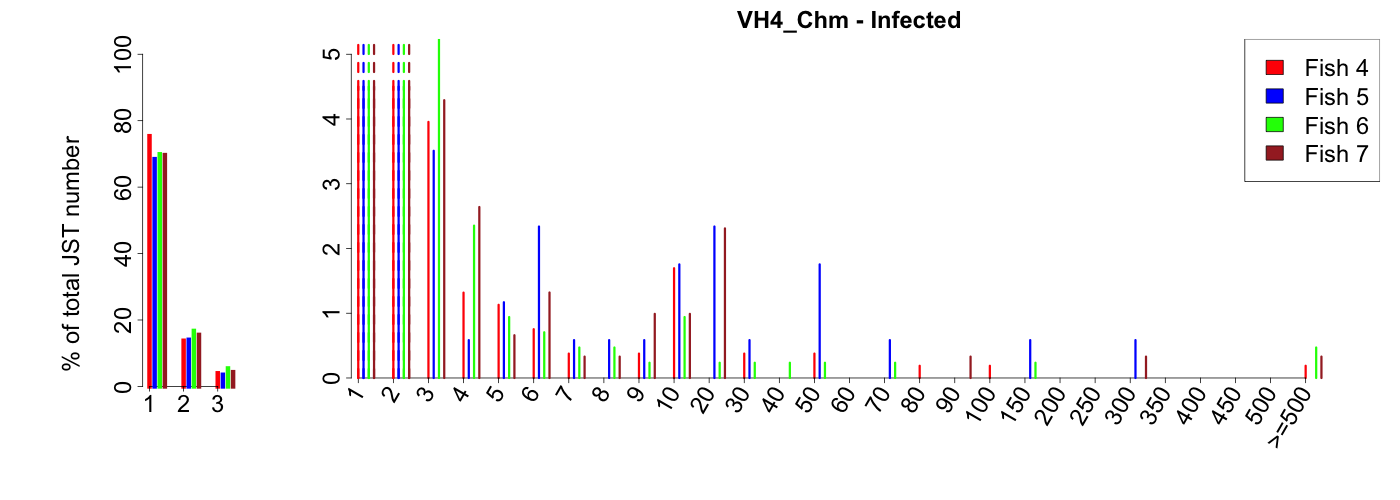 |
| **VH5.1Cμ Control fish corrected data**  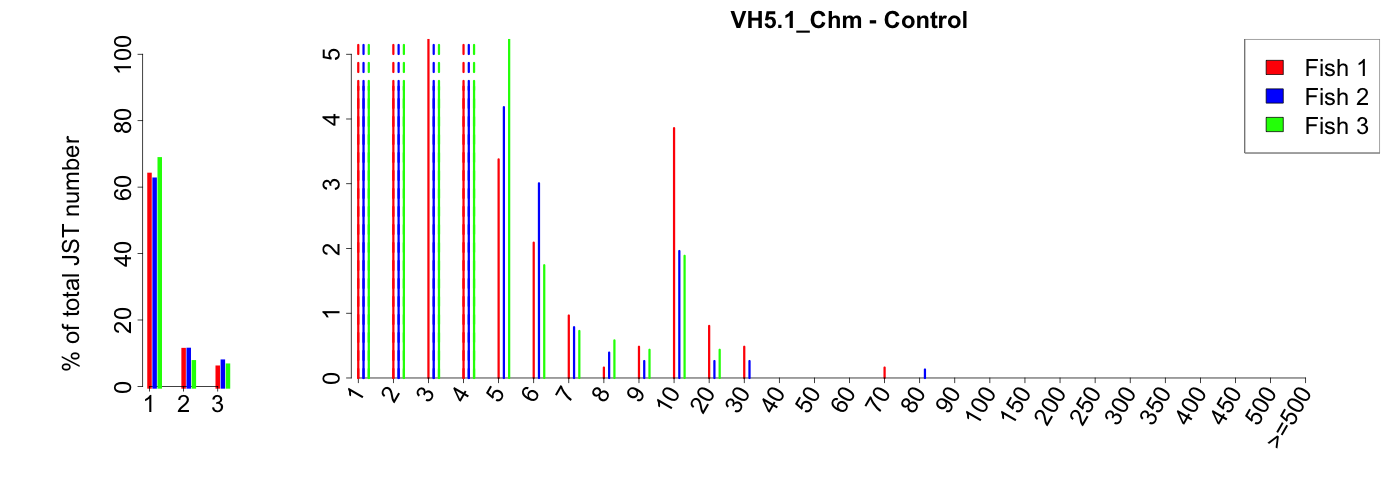 | **VH5.1Cμ Control fish unprocessed data**  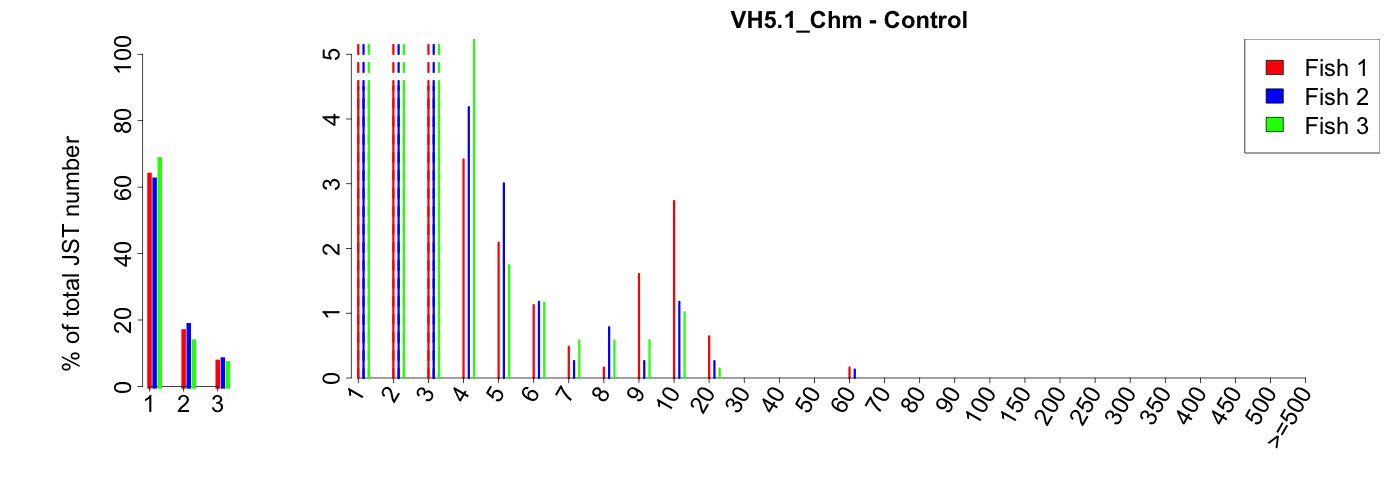 |
| **VH5.1Cμ Infected fish corrected data**  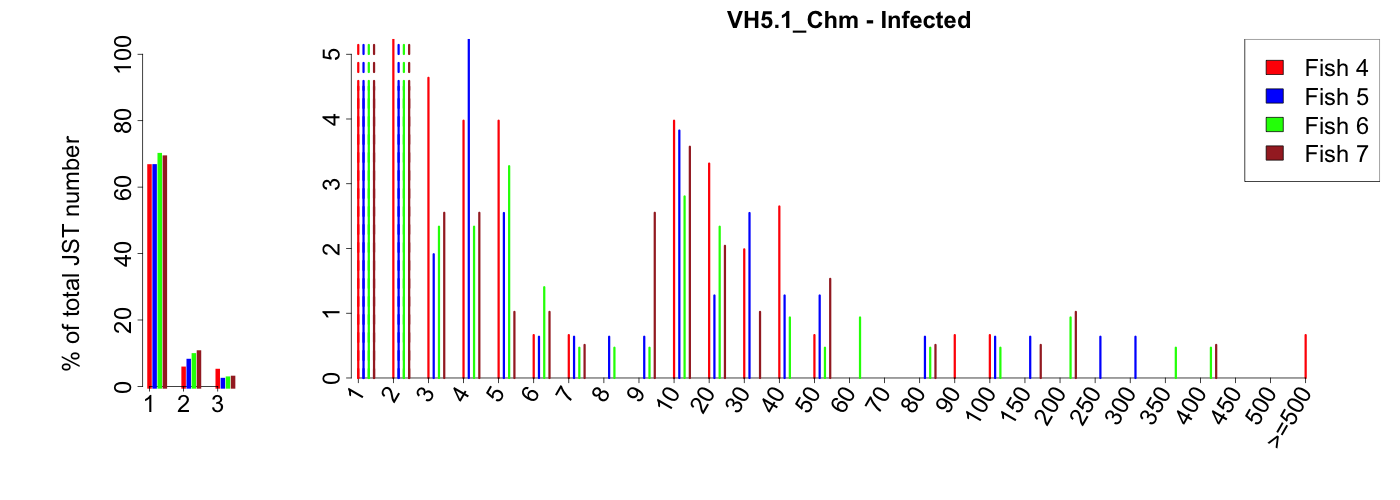 | **VH5.1Cμ Control fish unprocessed data**  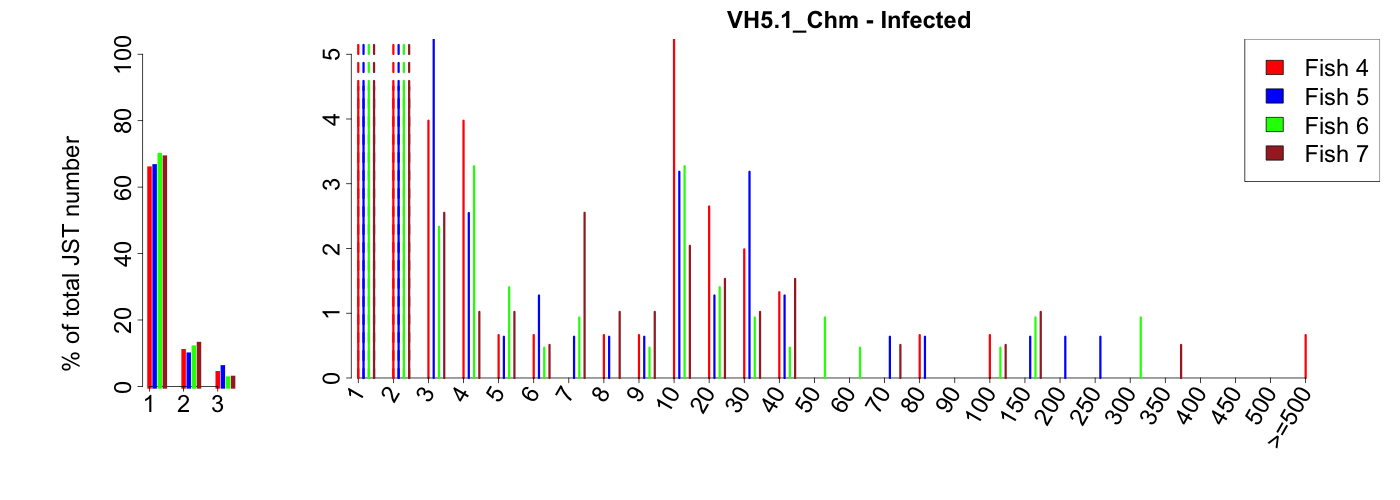 |
| **VH5.4Cμ Control fish corrected data**  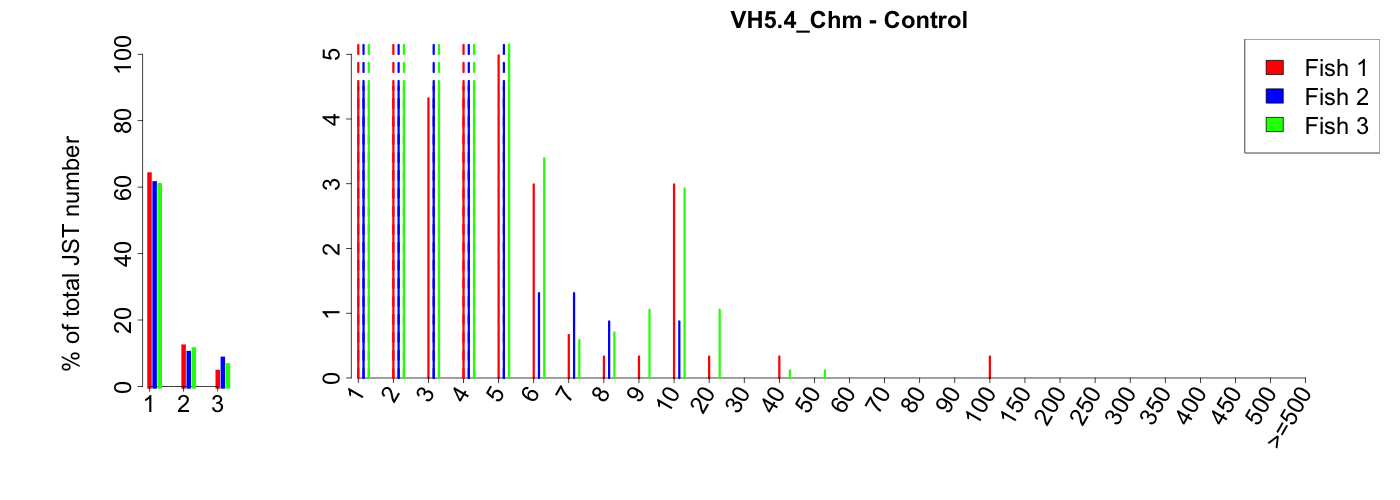 | **VH5.4Cμ Control fish unprocessed data**  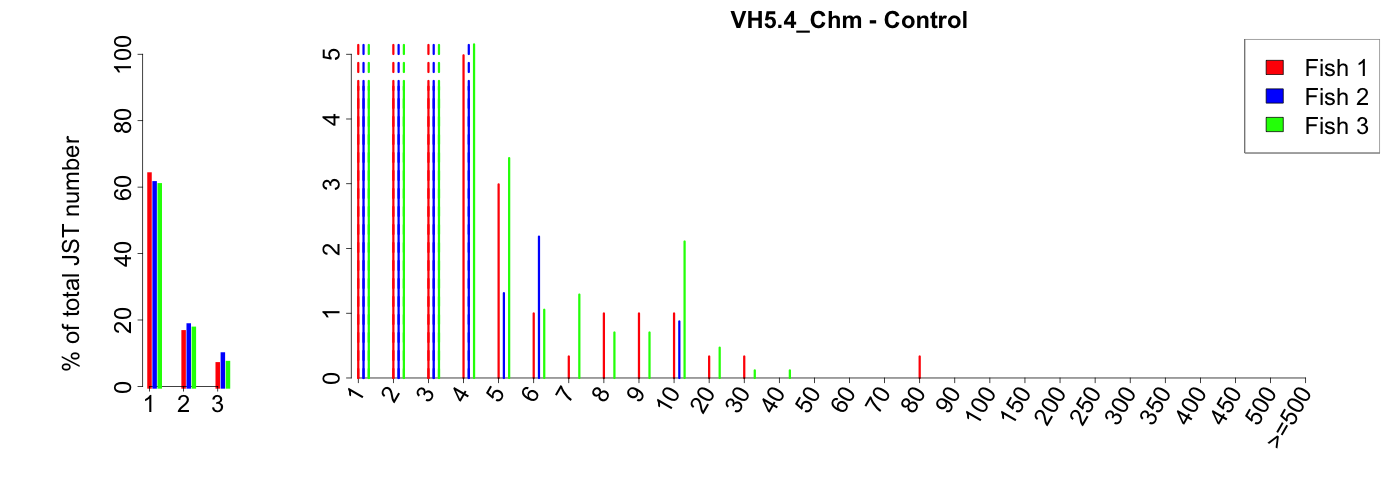 |
| **VH5.4Cμ Infected fish corrected data**  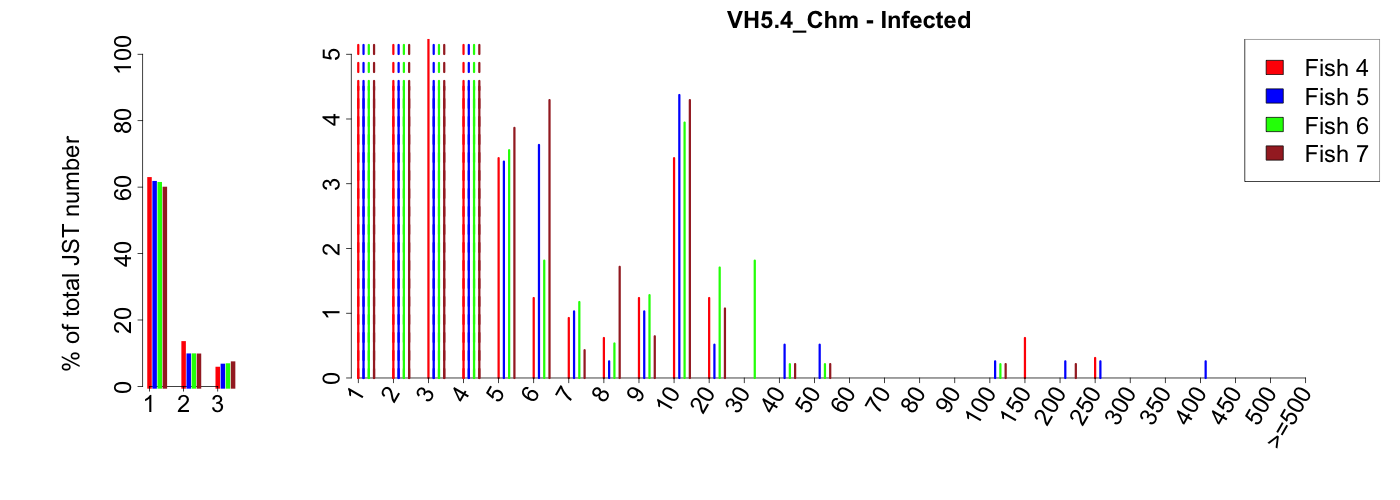 | **VH5.4Cμ infected fish unprocessed data**  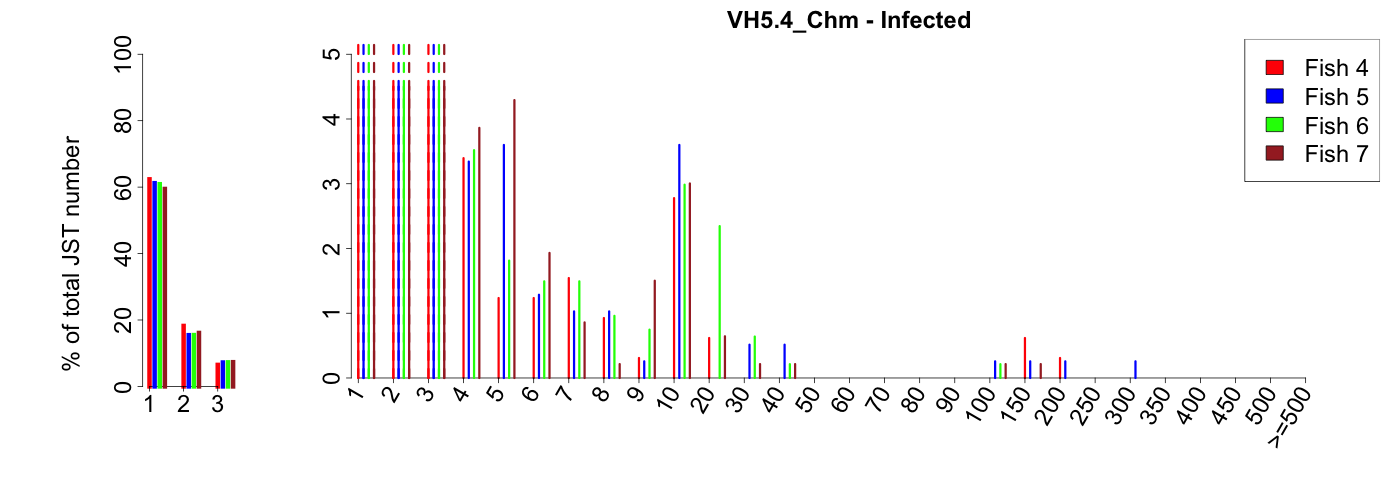 |
|  |  |
|  |  |
|  |  |
|  |  |
|  |  |
| **VH4Cτ control fish corrected data**  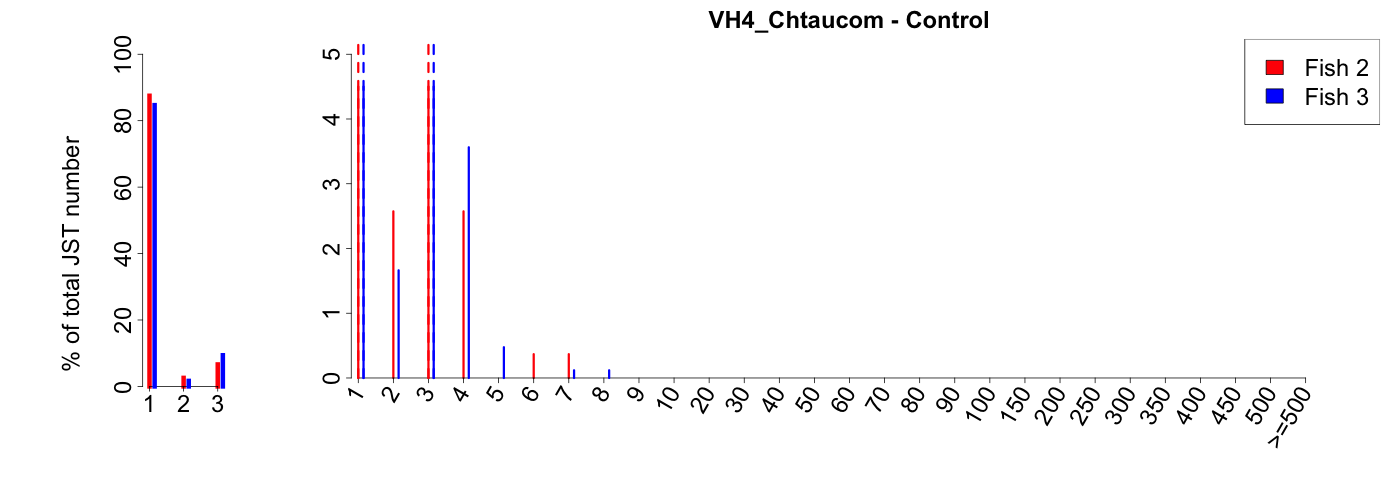 | **VH4Cτ control fish unprocessed data**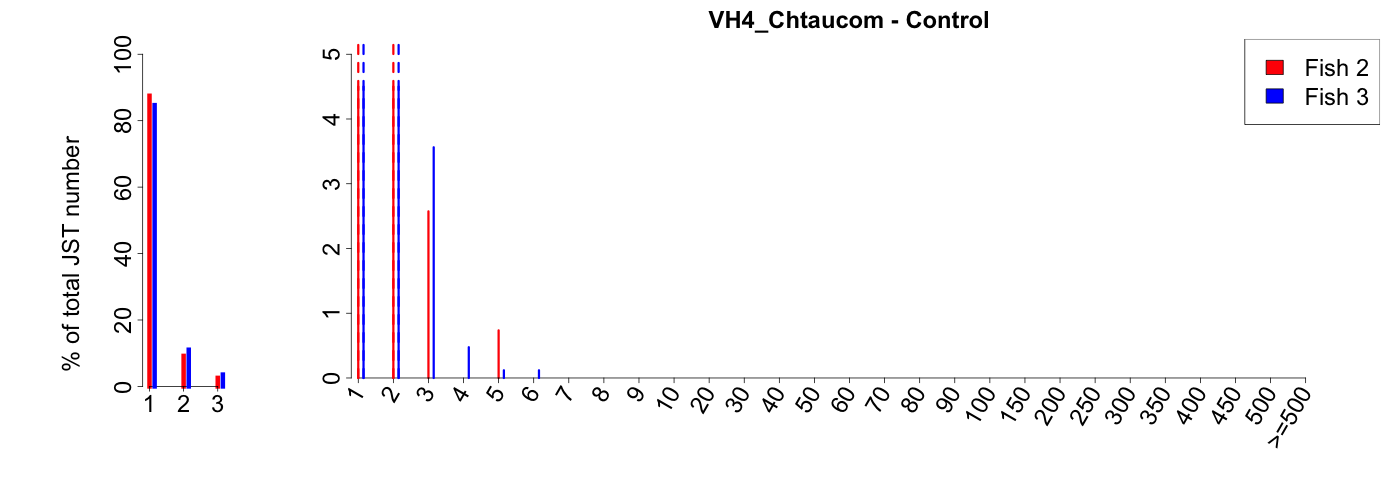 |
| **VH4Cτ infected fish corrected data**  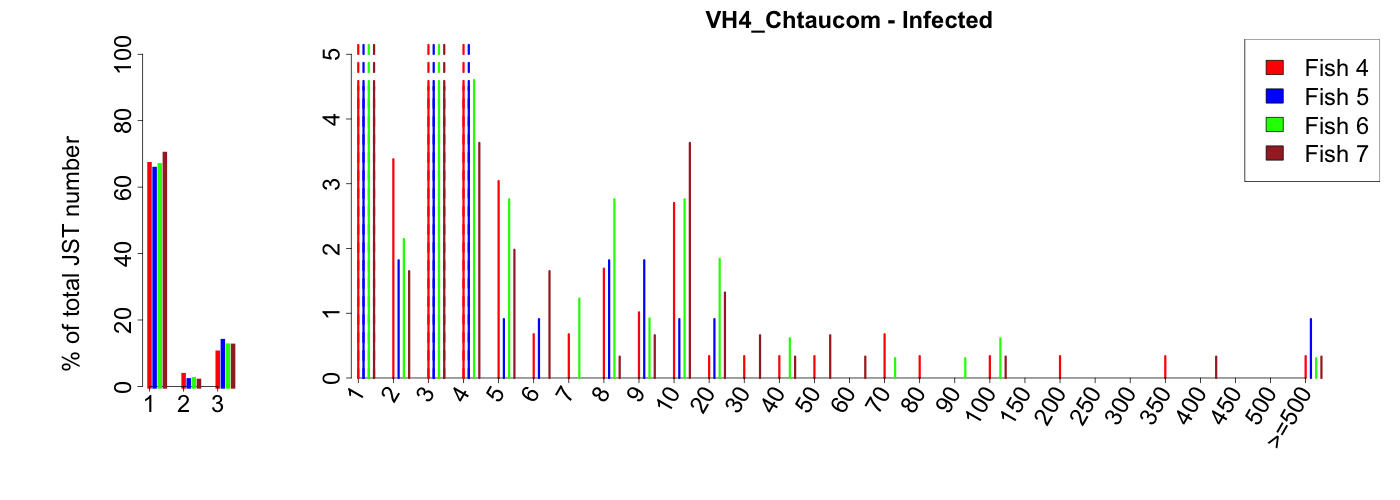 | **VH4Cτ infected fish unprocessed data**  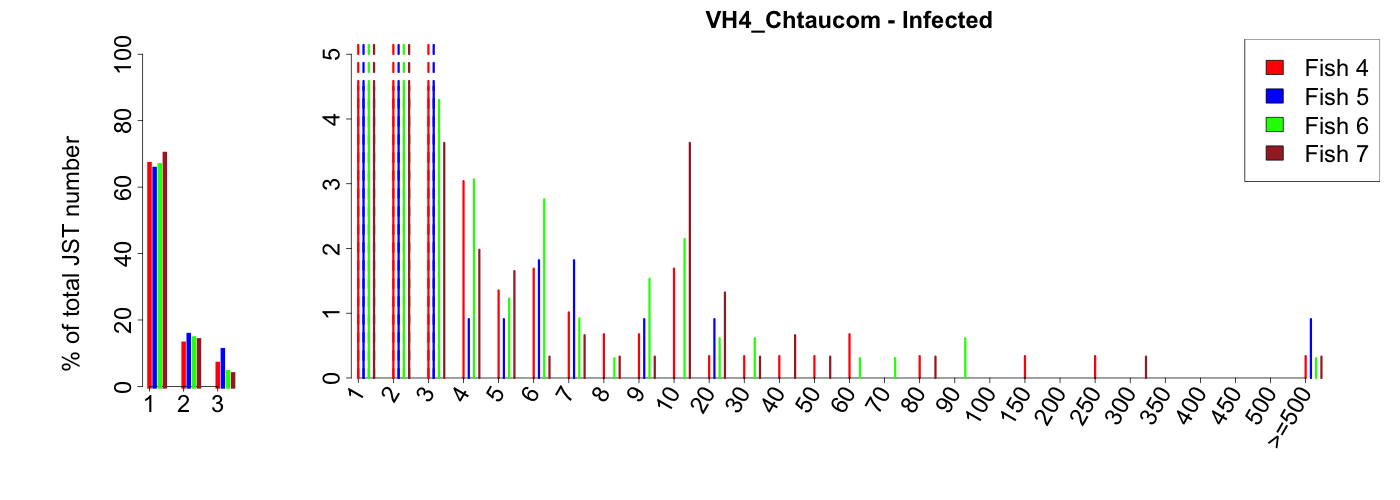 |
| **VH5.1Cτ control fish corrected data**  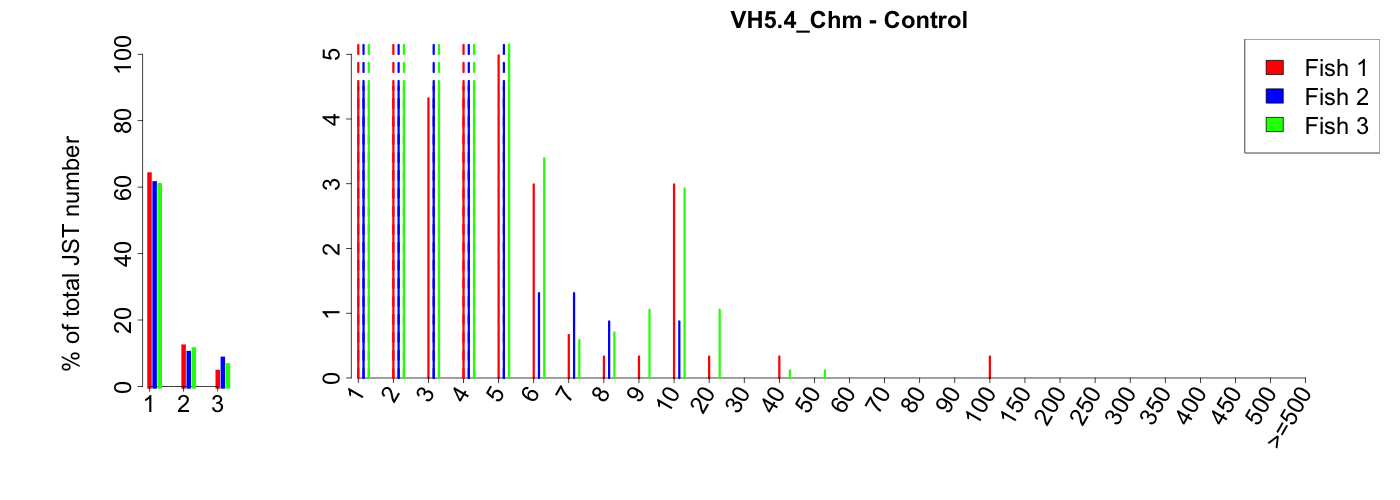 | **VH5.1Cτ control fish unprocessed data**  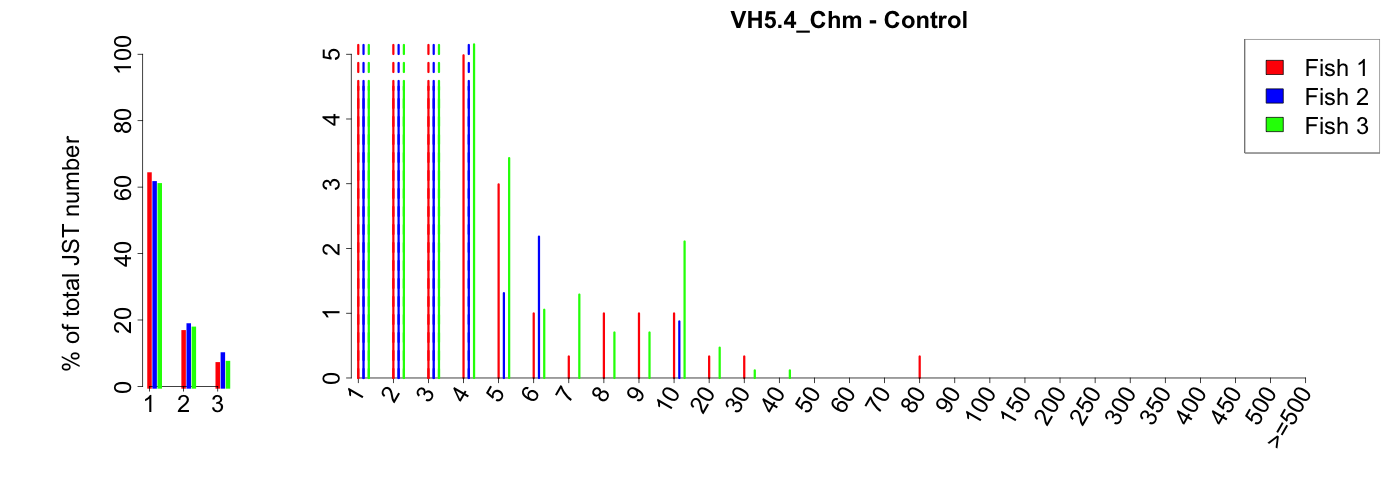 |
| **VH5.1Cτ infected fish corrected data**  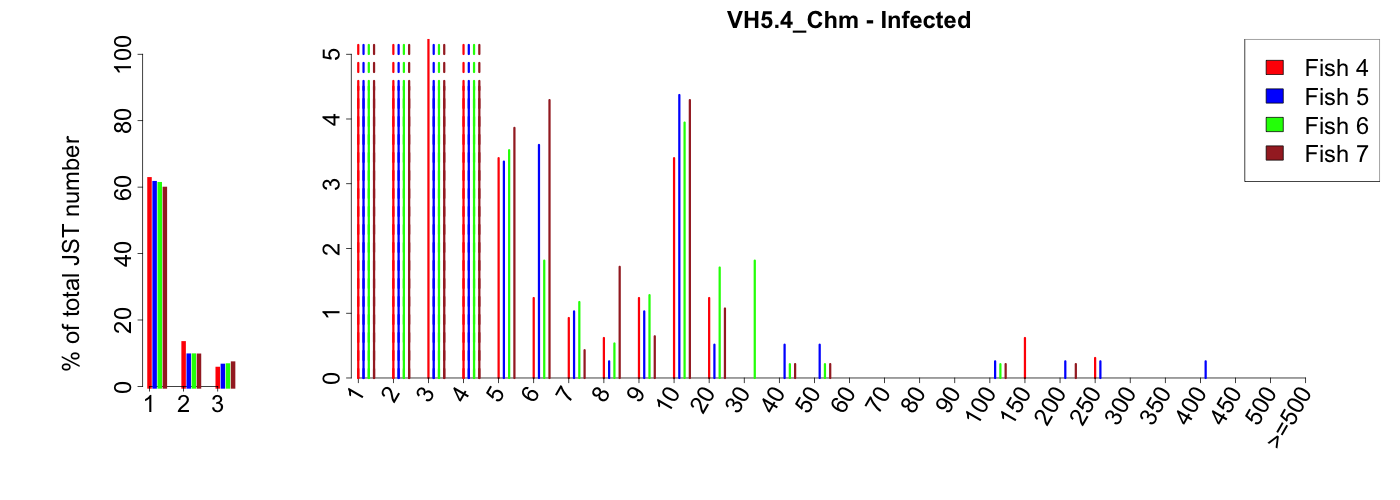 | **VH5.1Cτ infected fish unprocessed data**  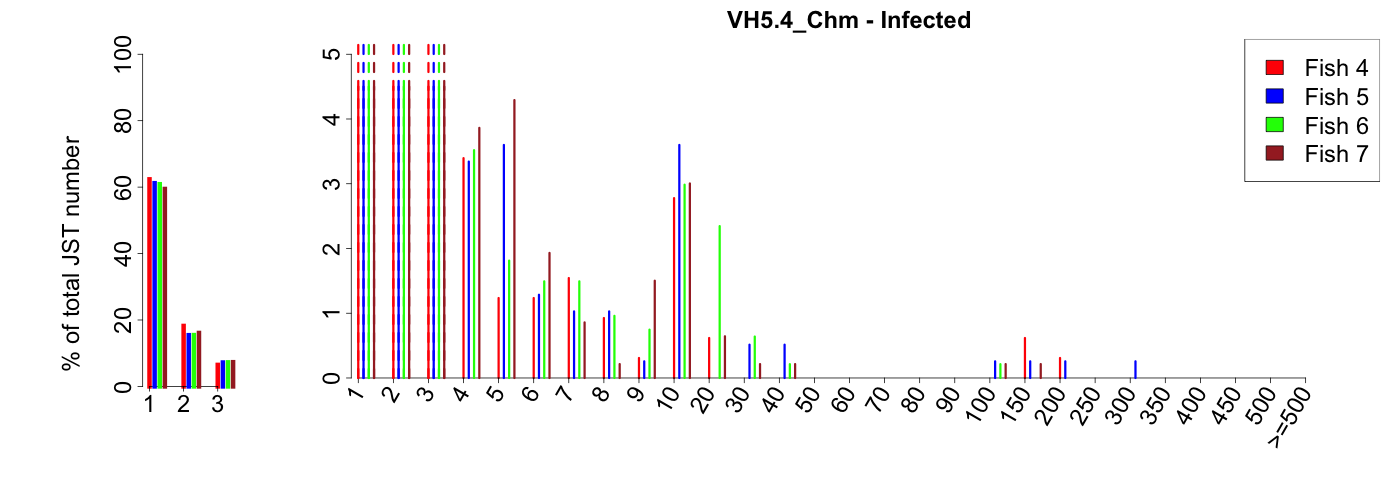 |
| **VH5.4Cτ control fish corrected data**  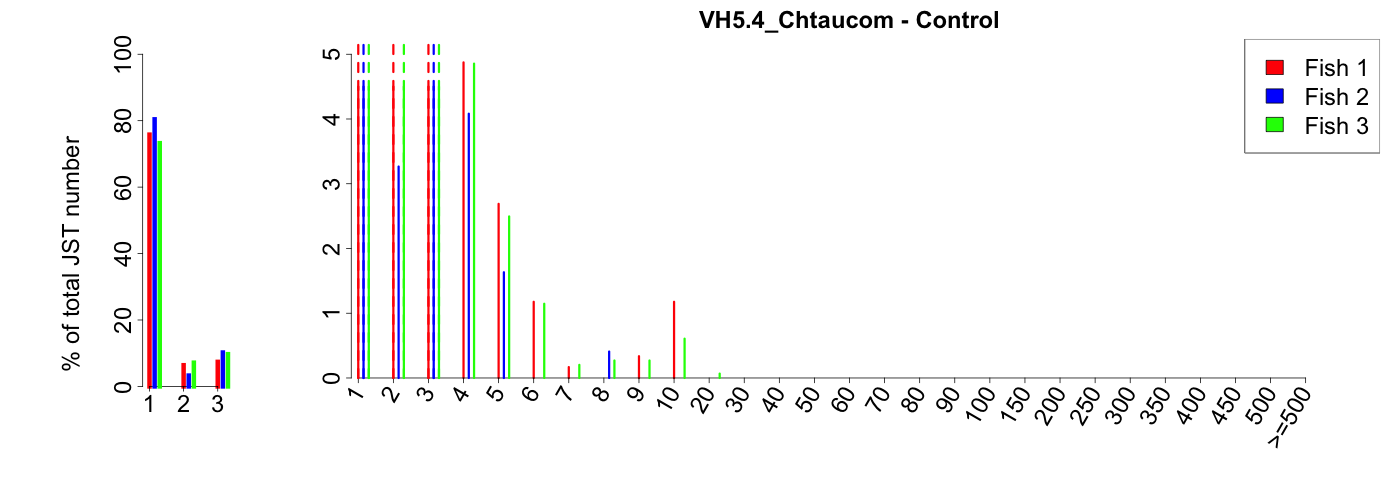 | **VH5.4Cτ control fish unprocessed data**  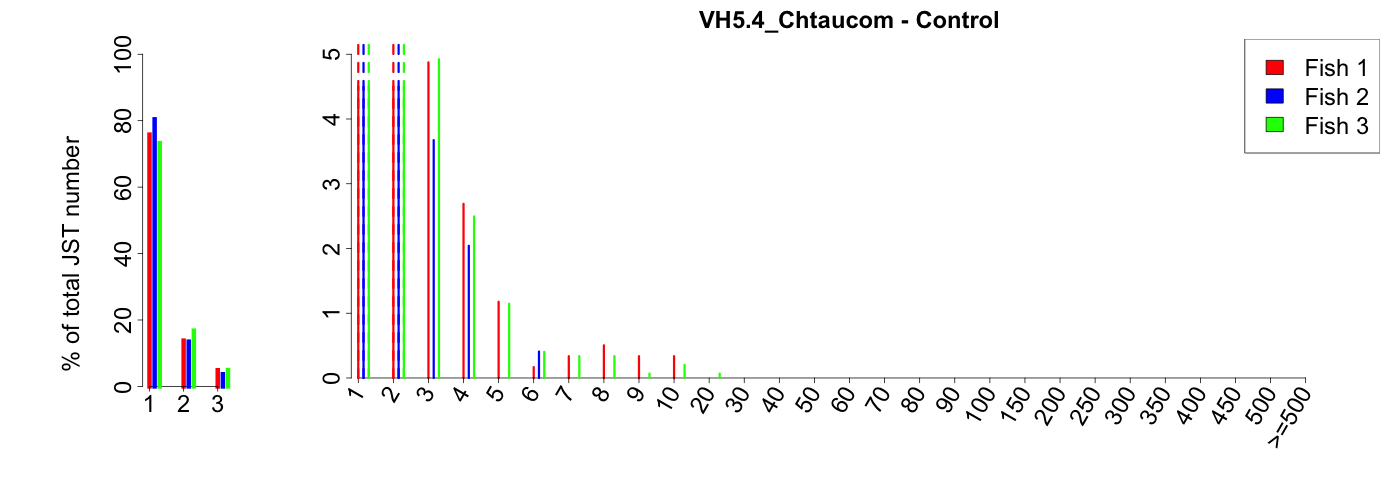 |
| **VH5.4Cτ infected fish corrected data**  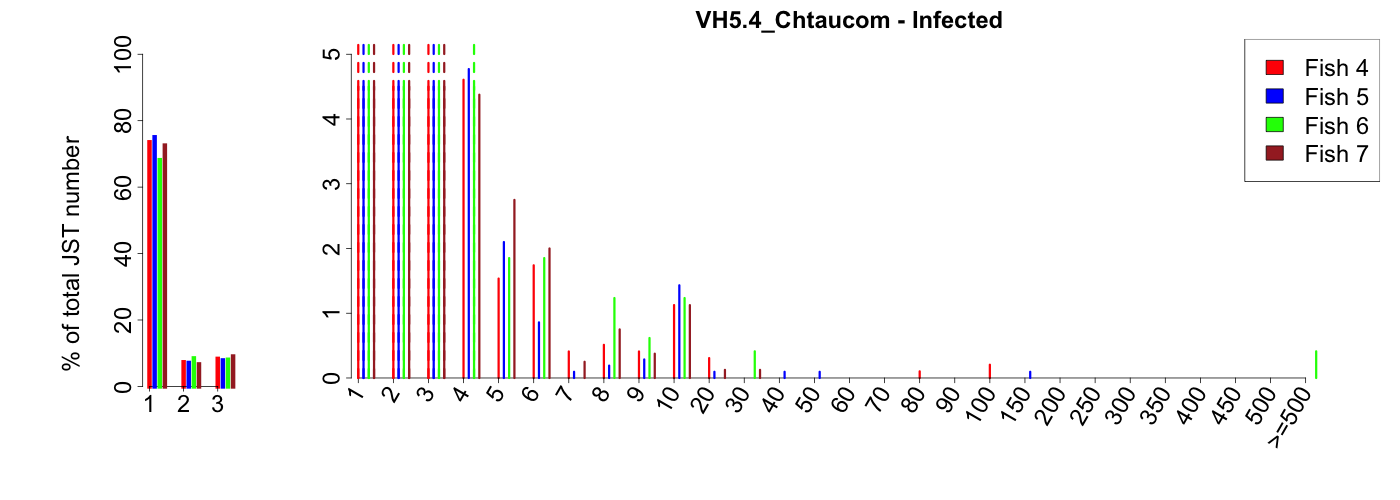 | **VH5.4Cτ infected fish unprocessed data**  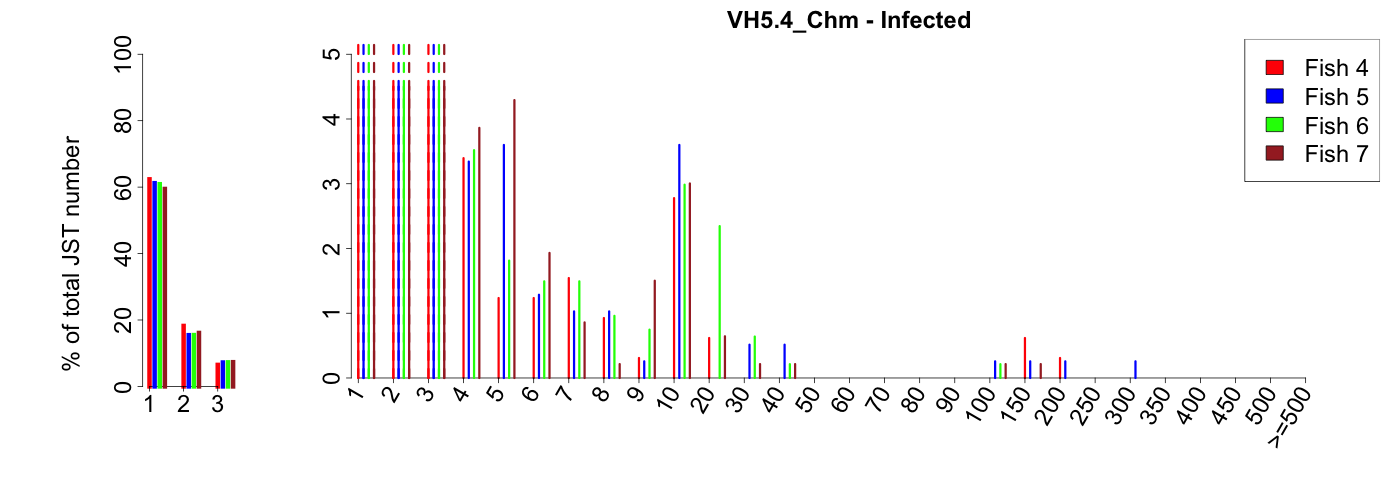 |

**C. Kolmogorov-Smirnov tests on junction type distributions from corrected datasets of 454 pyrosequencing of spleen VHCμ and VHCτ expressed rearrangements^1^**

|  | **Combination of fish subjected ^2^**  **to the KS test** | **Two-sided**  **p-values ^3^** |
| --- | --- | --- |
| IgM |  |  |
|  | **Infected vs controls** |  |
| VH4_C**μ** | All Ctrl vs All infected | 1.40E-03 ** |
| VH5.1_C**μ** | All Ctrl vs All infected | 4.80E-04 ** |
| VH5.4_C**μ** | All Ctrl vs All infected | **ns^3^** |
| VH4_C**μ** | One Ctrl vs One Inf (all combinations**^4^**) | All ** |
| VH5.1_C**μ** | One Ctrl vs One Inf (all combinations) | All ** but {1 vs 5} * |
| VH5.4_C**μ** | One Ctrl vs One Inf (all combinations) | All * or ** but {1 vs 4; 1 vs 6; 1 vs 7; and 3 vs 6}=**ns** |
|  | **Within controls:** |  |
| VH4_C**μ** | all combinations | **ns^4^** |
| VH5.1_C**μ** | all combinations | **ns** |
| VH5.4_C**μ** | all combinations | All **ns** but {1 vs 2}* and {2 vs 1_3} * |
|  | **Within infected:** |  |
| VH4_C**μ** | all combinations | All **ns** but {4 vs 5} * |
| VH5.1_C**μ** | all combinations | **ns** |
| VH5.4_C**μ** | all combinations | All **ns** but {5 vs 6} * |

| IgT |  |  |
| --- | --- | --- |
|  | **Infected vs controls** |  |
| VH4_C**τ** | All Ctrl vs All infected | 3.28E-08 *** |
| VH5.1_C**τ** | All Ctrl vs All infected | 2.87E-03 ** |
| VH5.4_C**τ** | All Ctrl vs All infected | 4.49E-5 *** |
| VH4_C**τ** | One Ctrl vs One Inf (all combinations) | All *** |
| VH5.1_C**τ** | One Ctrl vs One Inf (all combinations) | All ** but {1 vs 5=ns} and {2 vs 5=ns} |
| VH5.4_C**τ** | One Ctrl vs One Inf (all combinations) | All * or ** but  {1 vs 7; 2 vs 7 and 3 vs 7} ns |
|  | **Within controls:** |  |
| VH4_C**τ** | all combinations | ns |
| VH5.1_C**τ** | all combinations | ns |
| VH5.4_C**τ** | all combinations | ns |
|  | **Within infected:** |  |
| VH4_C**τ** | all combinations | All **ns** but {4 vs 5=*} |
| VH5.1_C**τ** | all combinations | ns |
| VH5.4_C**τ** | all combinations | All ns but {4 vs 6; 5 vs 6 ; 6 vs 7 ; 5 vs 4_6; 6 vs 4_5; 6 vs 4_7; 7 vs 4_6; 4_5 vs 6_7 ; 4_6 vs 5_7 ; 4_7 vs 5_6 ; 6 vs 4_5_7 ; 7 vs 4_5_6}* or ** |

^1^ The procedure to compute KS tests and to aggregate JST from different fish is detailed in Figure S8. Aggregated distributions are denoted by « _ » linking the corresponding fish number. Control fish are 1-3 and infected fish 4-7.

^2^ KS test was applied to different combinations of distributions: (1) All control (aggregated) versus all infected (aggregated) (2) One Ctrl vs One Inf (all combinations): each control versus each infected individual distribution; (3) All combinations: each individual or aggregated distribution versus all other individual or aggregated distributions, within control or infected respectively. For example {1 vs 2 ; 1 vs 3 ; 2 vs 3 ; 1 vs 2_3 ; 2 vs 1_3 ; 3 vs 1_2 } within control fish.

^3^ 'Two-sided' analysis tests if the distribution in Infected samples is equal to the distribution of Control samples (i.e.H0, null hypothesis of the KS test). P-values < 5% *; <1% **; <0.1% *** indicate that the distributions are significantly different.

^4^ 'One-sided' analysis tests if the distribution in Infected samples is shorter than in Control samples (H0).

P-values < 5% *; <1% **; <0.1% *** indicate that the distributions in control fish are significantly shorter than in infected fish.

^5^ NS: non significant, i.e. H0= “equal distribution” is not rejected.
