# Supplementary material for: Teleost Fish Mount Complex Clonal IgM and IgT Responses in Spleen upon Systemic Viral Infection
Source: PLoS Pathog. 2013 Jan 10;9(1):e1003098. doi: 10.1371/journal.ppat.1003098 (PMC3542120; doi:10.1371/journal.ppat.1003098)
Supplement: Figure S6 — Comparison of CDR3 length profiles from Immunoscope spectratyping and 454 pyrosequencing. The CDR3 length profiles were computed from 454 sequence data and compared to the immunoscope spectratypes from the same fish. (A) shows that the distributions were quasi-identical, thus providing an independent validation of the repertoire description by 454 sequencing. Additionally, when a VHJμ PCR product was cloned, and 160 clones sequenced at random, the junction frequencies were consistent with two 454 independent sets of sequences (B). We therefore conclude that 454 pyrosequencing produces a representation of the junction repertoire that is in good accordance with widely used former technologies. (DOCX) [file ppat.1003098.s006.docx]

**Figure S6.**

**Comparison of CDR3 length profiles from Immunoscope spectratyping and 454 pyrosequencing.** The CDR3 length profiles were computed from 454 sequence data and compared to the immunoscope spectratypes from the same fish. (A) shows that the distributions were quasi-identical, thus providing an independent validation of the repertoire description by 454 sequencing. Additionally, when a VHJμ PCR product was cloned, and 160 clones sequenced at random, the junction frequencies were consistent with two 454 independent sets of sequences (B). We therefore conclude that 454 pyrosequencing produces a representation of the junction repertoire that is in good accordance with widely used former technologies.

| A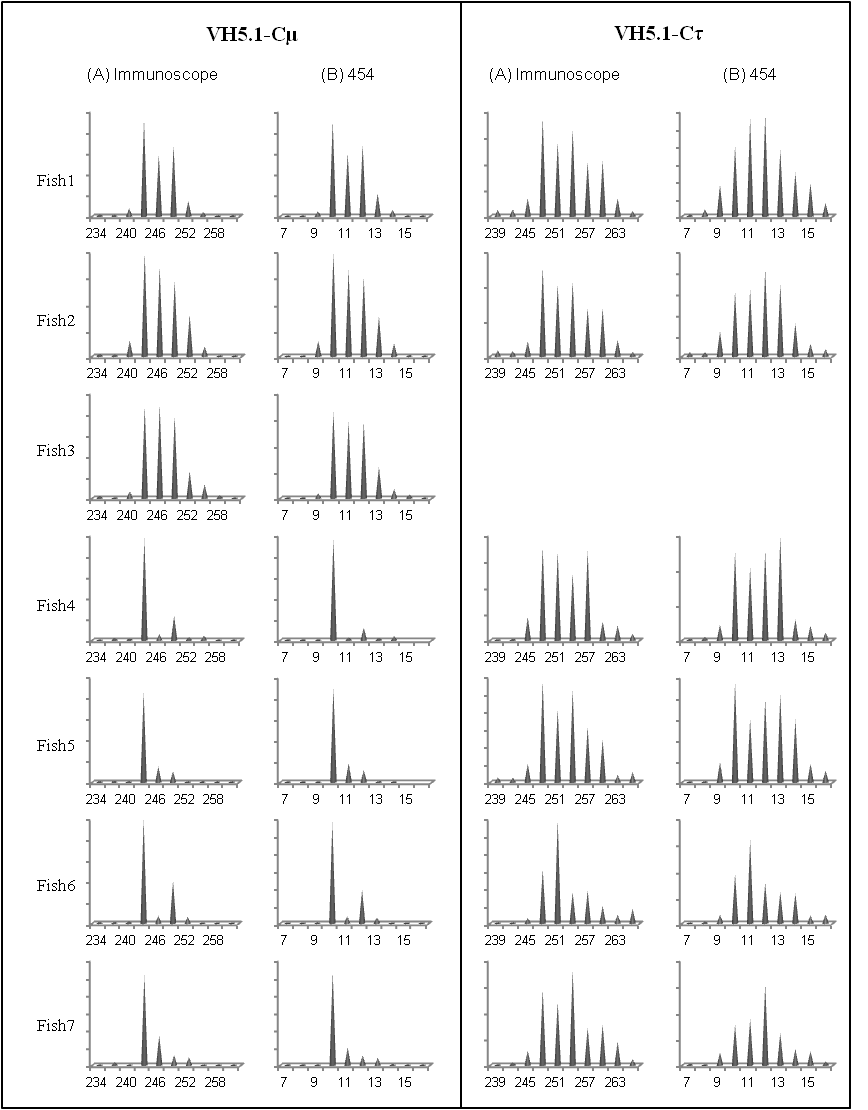 | B   \|  \| 454 \| \| TOPO TA \| \| --- \| --- \| --- \| --- \| \| IGHV6 IGHJ6*01 \| run4 \| run 2 \| cloning \| \| % of sequences \|  \|  \|  \| \| CAQITDYRFDYW \| 25.45 \| 16.24 \| 16.18 \| \| CAQKTTDSFDYW \| 1.87 \| 5.98 \|  \| \| CARGTTGVNRFDYW \| 0.02 \|  \| 7.35 \| \| CARGVTLGSTPVFDYW \| 0.91 \| 0.85 \|  \| \| CARHNYGTYRFDYW \| 0.86 \| 3.42 \|  \| \| CARITDYRFDTW \| 0.02 \|  \| 1.47 \| \| CARITTARFDYW \| 27.71 \| 35.04 \| 30.88 \| \| CARITTARSDYW \| 0.15 \|  \| 1.47 \| \| CARLYDRFDYW \| 1.28 \| 1.71 \|  \| \| CARNNDRFDYW \| 0.22 \|  \| 1.47 \| \| CARNYDRFDYW \| 9.21 \| 5.13 \| 4.41 \| \| CARQGFDYW \| 1.06 \|  \| 1.47 \| \| CARRIDYRFDYW \| 5.77 \| 2.56 \| 4.41 \| \| CARVTTARFDYW \| 0.07 \| 1.71 \| 1.47 \| |
| --- | --- | --- | --- | --- | --- | --- | --- | --- | --- | --- | --- | --- | --- | --- | --- | --- | --- | --- | --- | --- | --- | --- | --- | --- | --- | --- | --- | --- | --- | --- | --- | --- | --- | --- | --- | --- | --- | --- | --- | --- | --- | --- | --- | --- | --- | --- | --- | --- | --- | --- | --- | --- | --- | --- | --- | --- | --- | --- | --- | --- | --- | --- | --- | --- | --- | --- | --- | --- | --- |
