# Supplementary material for: Teleost Fish Mount Complex Clonal IgM and IgT Responses in Spleen upon Systemic Viral Infection
Source: PLoS Pathog. 2013 Jan 10;9(1):e1003098. doi: 10.1371/journal.ppat.1003098 (PMC3542120; doi:10.1371/journal.ppat.1003098)
Supplement: Figure S7 — Complementary data from 454 pyrosequencing. (A) JH usage in VH4Cμ expressed rearrangements. (B) Normalized distributions of JST observed n times in the sequence datasets from control and virus infected fish are represented for VH5.4 Cμ (C) Normalized distributions of JST observed n times in the sequence datasets from control and virus infected fish are represented for VH5.4 Cτ. (PDF) [file ppat.1003098.s007.pdf]

Figure S7. Complementary data from 454 pyrosequencing.

A. JH usage in VH4Cμ expressed rearrangements.

V4-Cμ

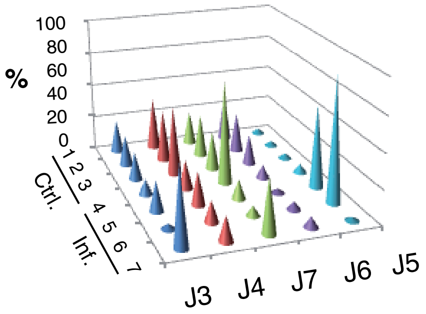

B. Normalized distributions of JST observed n times in the sequence datasets from control and virus infected fish are represented for VH5.4 Cμ

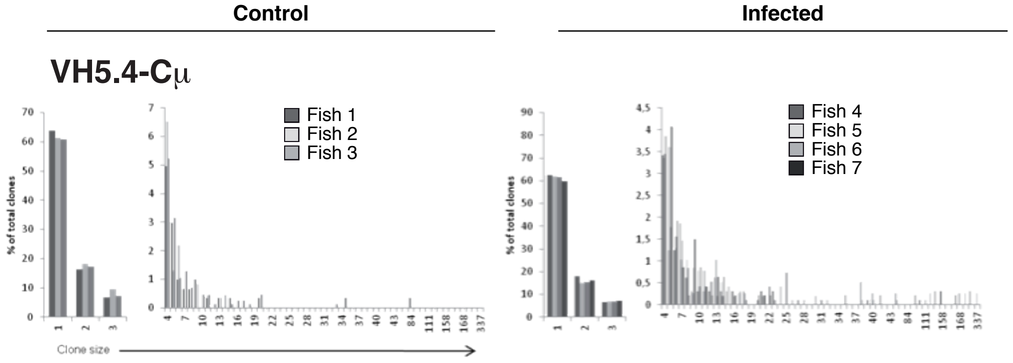

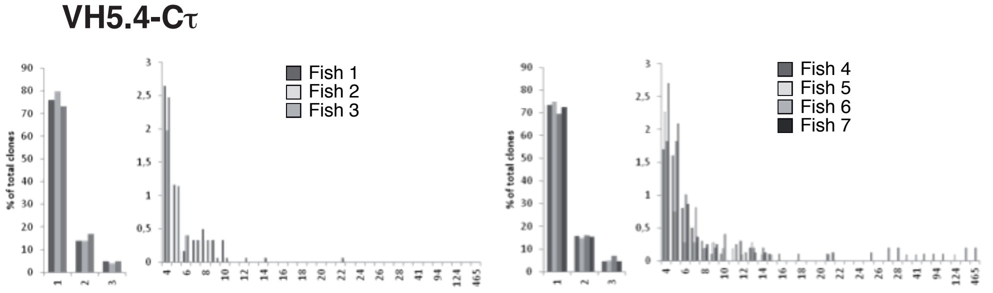

C. Normalized distributions of JST observed n times in the sequence datasets from control and virus infected fish are represented for VH5.4 Cτ.
