# Supplementary material for: Teleost Fish Mount Complex Clonal IgM and IgT Responses in Spleen upon Systemic Viral Infection
Source: PLoS Pathog. 2013 Jan 10;9(1):e1003098. doi: 10.1371/journal.ppat.1003098 (PMC3542120; doi:10.1371/journal.ppat.1003098)
Supplement: Figure S8 — Kolmogorov-Smirnov (KS) statistics on junction sequence type (JST) distributions. (A) Procedure for aggregation of data from different fish to compute KS tests. (B) KS tests on JST distributions from unprocessed 454 pyrosequencing datasets of spleen VHCτ expressed rearrangements. (DOCX) [file ppat.1003098.s008.docx]

**Figure S8. Kolmogorov-Smirnov statistics on junction sequence type (JST) distributions**

**A. Procedure for aggregation of data from different fish to compute KS tests**

To compare control and infected sequence-type occurrence distributions (SOD) for a given V/C combination, distribution values belonging to the same condition (control or infected) were summed. One summed SOD is therefore obtained for each of the two conditions, hereafter called sequence occurrence distribution per conditions (SODPC).

The total number of sequences was count per condition and each value of the SODPC divided by this total, producing two normalized SODPC (NSODPC).

We considered the following categories of number of occurrences for sequence types:

Categories 1 to 9, corresponds to 1 to 9

occurrences

Category 10 corresponds to the sum of occurrences from 10 to 19

Category 11 corresponds to the sum of occurrences from 20 to 29

...

Category 18 corresponds to the sum of occurrences from 90 to 99

Category 19 corresponds to the sum of occurrences from 100 to 149

Category 20 corresponds to the sum of occurrences from 150 to 199

...

Category 27 corresponds to the sum of occurrences from 450 to 499

Category 28 corresponds to the sum of occurrences greater or equal to 500

The values of NSODPC belonging to the same category were summed, producing two “category occurrences distributions” (COD).

We finally summed categories:

Cumul 1 = Category 1

Cumul 2 = Cumul 1 + Category 2

...

Cumul i = Cumul (i-1) + Category 2

and got two “cumulated categories occurrences distributions” (CGOD).

We apply Kolmogorv-Smirnov tests on these CGOD with the hypothesis

  (H0) “the distribution in samples A is equal to distribution of samples B”

('Two-sided' p value)

**B. Kolmogorov-Smirnov tests on JST distributions from unprocessed 454 pyrosequencing datasets of spleen VHCτ expressed rearrangements^1^**

*The table computed from the dataset after error correction is shown Figure S5C.*

|  | **Combination of fish subjected** ^2^  **to the KS test** | Two-sided^3^  p-values | One-sided^4^  p-values |
| --- | --- | --- | --- |
|  | **Infected vs controls** |  |  |
| VH4_Cτ | All Ctrl vs All infected | 6.19E-09 *** | 3.09E -09*** |
| VH5.1_Cτ | All Ctrl vs All infected | 6.39E-04 * | 3.19E -04*** |
| VH5.4_Cτ | All Ctrl vs All infected | 3.48E-04 *** | 1.74E -04*** |
| VH4_Cτ | One Ctrl vs One Inf (all combinations) | All *** | All *** |
| VH5.1_Cτ | One Ctrl vs One Inf (all combinations) | All * but {2 vs 7 } NS^5^ | All * but {2 vs 7 } NS |
| VH5.4_Cτ | One Ctrl vs One Inf (all combinations) | All* but {1 vs 7 ; 2 vs 7 ; 3 vs 7} NS | All but {1 vs 7 ; 3 vs 7} NS |
|  | **Within controls:** |  |  |
| VH4_Cτ | all combinations | NS^5^ | NS |
| VH5.1_Cτ | all combinations | NS | NS |
| VH5.4_Cτ | all combinations | NS | NS |
|  | **Within infected:** |  |  |
| VH4_Cτ | all combinations | All NS but {5 vs 7 } * | NS |
| VH5.1_Cτ | all combinations | NS | NS |
| VH5.4_Cτ | all combinations | All NS but {4 vs 6; 5 vs 6 ; 6 vs 7 ; 5 vs 4_6; 6 vs 4_5; 6 vs 4_7; 7 vs 4_6; 4_5 vs 6_7 ; 4_6 vs 5_7 ; 4_7 vs 5_6 ; 6 vs 4_5_7 ; 7 vs 4_5_6}* or ** | All NS but {4 vs 6; 5 vs 6 ; 5 vs 4_6; 7 vs 4_5; 7 vs 4_6 ; 4_5 vs 6_7 ; 5_7 vs 4_6 ; 7 vs 4_5_6}* or ** |

^1^ The procedure to compute KS tests and to aggregate JST from different fish is detailed in Figure S8. Aggregated distributions are denoted by « _ » linking the corresponding fish number. Control fish are 1-3 and infected fish 4-7.

^2^ KS test was applied to different combinations of distributions: (1) All control (aggregated) versus all infected (aggregated) (2) One Ctrl vs One Inf (all combinations): each control versus each infected individual distribution; (3) All combinations: each individual or aggregated distribution versus all other individual or aggregated distributions, within control or infected respectively. For example {1 vs 2 ; 1 vs 3 ; 2 vs 3 ; 1 vs 2_3 ; 2 vs 1_3 ; 3 vs 1_2 } within control fish.

^3^ 'Two-sided' analysis tests if the distribution in Infected samples is equal to the distribution of Control samples (i.e.H0, null hypothesis of the KS test). P-values < 5% *; <1% **; <0.1% *** indicate that the distributions are significantly different.

^4^ 'One-sided' analysis tests if the distribution in Infected samples is shorter than in Control samples (H0).

P-values < 5% *; <1% **; <0.1% *** indicate that the distributions in control fish are significantly shorter than in infected fish.

^5^ NS: non significant, i.e. H0= “equal distribution” is not rejected.
