# Supplementary material for: Teleost Fish Mount Complex Clonal IgM and IgT Responses in Spleen upon Systemic Viral Infection
Source: PLoS Pathog. 2013 Jan 10;9(1):e1003098. doi: 10.1371/journal.ppat.1003098 (PMC3542120; doi:10.1371/journal.ppat.1003098)
Supplement: Figure S9 — Sampling model of the IgHμ and IgHτ 454 pyrosequencing. Does a given JST represent an activated, amplified versus a resting, non amplified B cell clone? Do abundant JST reflect non amplified B cell clones? Consequences for the identification of public and private responses. (PDF) [file ppat.1003098.s009.pdf]

Figure S9. Sampling model of the IgHμ and IgHτ 454 pyrosequencing.

Does a given JST represent an activated, amplified versus a resting B cell clone? Do abundant JST reflect activated B cell clones? Consequences for the identification of public and private responses

Contents

1 Introduction 2

2 The Model 2

3 Connection to Hyper-geometric Distributions 4

4 Statistical Tests at the JST Level for a Given Individual i 6

4.1 Outline . . . 6

4.2 Testing H_0^- . . . 7

4.3 Testing H_0^+ . . . 7

5 Implementation Note 7

6 Assessing Public and Private Responses from Observed JSTs 8

6.1 Method . . . 8

6.2 Filling the urns . . . 8

6.3 One-sided p-values curves for increasing values of the number of sequences $s_{i;j}$ observed for the jth clone . . . 9

6.4 Results . . . 10

6.4.1 Does a large JST found in one clone support H_0^+? . . . 10

6.4.2 The case of private responses . . . 10

6.4.3 The case of public responses . . . 10

1 Introduction

Infection and clones. We wish to count and compare the IGH transcripts across control versus infected fish. A given B cell expresses a particular IGH junctional sequence (i.e. a particular JST; the junction sequence type (JST) being defined as a CDR3 protein sequence associated with a (V,J) pair). When B a cell gets activated for example after binding the antigen, it is activated and proliferates to a larger clone expressing the same JST. Consequently, the corresponding mRNA gets more abundant for two reasons: (1) more mRNA are produced by activated cells than by resting cells and (2) cells expressing this JST are more numerous. Deep sequencing produces datasets that reflect the structure of the IGH mRNAs expressed by B cells, whence the structure of the B cell population itself; the aim of this modeling is to test whether large JSTs identify responding B cell clones, or may be explained by sampling artifacts from the mRNA population.

For a set of (control or infected) fish, a repeated JST may typically fall into one of the following three categories:

- The repeated JST occurs in all infected fish, not in control ones. Such a sequence is expected to be involved in a so-called *public response*.
- The repeated JST occurs in a (few) specific infected individual only. Such a sequence is expected to be involved in a so-called *private response*.
- The repeated sequence is found in a (set of) control and/or infected organisms, generally at relatively low level. Such a sequence, which corresponds to a clone of cells within that individual, likely corresponds to the track of a previous exposure to another pathogen(/antigen). The presence of such clones reflects that fish are not fully "naive".

It should also be noted that in-between two individuals, the number of common JSTs is *low*, since the number of cells (whence sequences) in the spleen of an individual (available repertoire) is small compared to the total number of possible JSTs (potential repertoire). JSTs involved in public responses represent exceptions to this rule; in fact, they may been selected during the evolution of the species to cope with frequent pathogens.

2 The Model

In the following, we present the model summarized on Fig. 1, describing how reads of a given JST are sampled from the B cell population of an individual O_i.

Populations of IGH mRNAs expressed by spleen B cells. The initial effective population of mRNA (p_i) corresponds to a given isotype and a given VH family, i.e. to the population in which the PCR amplification performs the sampling of templates for sequencing.

This initial pool of mRNA is denoted:

$$\mathbf{P}(O_i) = \{p_{i;j}\}_{j=1,\ldots,d_i}, \quad \text{with } \sum_j p_{i;j} = p_i. \tag{1}$$

where i stands for the ith individual, and j in $\text{JST}_j j$ is the index of the relevant clone.

Sampling and sequencing. During the first steps of the analysis, spleen cells are prepared, RNA is extracted and part of it used for cDNA synthesis. Although the whole spleen was always used for mRNA extraction, these steps represent a first round of sampling, since all the mRNA molecules from all B cells present in the organ are not recovered and cannot be used for the cDNA synthesis.

Sampling the previous pool by picking for instance 1/1000 molecule yields a random sample of size $r_i = p_i/1000$, that is:

$$\mathbf{R}(O_i) = \{r_{i;j}\}_{j=1,\ldots,d_i}, \quad \text{with } \sum_j r_{i;j} = r_i. \tag{2}$$

These cDNAs are then analyzed by to PCR amplification with VH/C primer pairs and PCR products sent to deep sequencing.

Completing this process yields a total of s_i sequences per PCR product (i.e. per VH/C combination) and per individual, this number being called the *sequencing effort* of the individual. The sequences generated by the process can be unique or repeated, whence the following vector which counts the number of copies of all possible sequences:

$$\mathbf{S}(O_i) = \{s_{i;j}\}_{j=1,\dots,d_i}, \text{ with } \sum_j s_{i;j} = s_i. \tag{3}$$

Unique and repeated sequences. JSTs comprising high number of sequence reads are more abundant and have a larger size in VHSV-infected compared to control individuals, suggesting that such JSTs indeed represent expanded clones specific for viral epitopes. The present model allows testing this hypothesis.

We assume the existence of a threshold s_{max} such that a *unique* sequence satisfies

$$s_{i;j} \leq s_{max}. \tag{4}$$

We also assume the existence of a threshold s_{min}, such that a *repeated sequence* satisfies

$$s_{i;j} \geq s_{min}. \tag{5}$$

Practically, we shall consider that $s_{max} = s_{min} = 10$.

Figure 1 Model overview at the individual level. The top row features all the cells of an individual expressing a given VH/C. These cells are partitioned into activated and resting clones. The **second** and **intermediate** layers correspond to pools of mRNA, while the **bottom** layer corresponds to JST, which are tagged as unique or repeated depending on their counts. These three layers are modeled by urns, from which hyper-geometric distributions are defined, and p-values computed. Boxes at the bottom indicate that one can estimate whether a given JST is produced by a resting or an activated clone, depending on the sequence count and the thresholds of Eq. (4, 5). Red boxes indicate wrong qualifications, while blue boxes indicate correct qualifications.

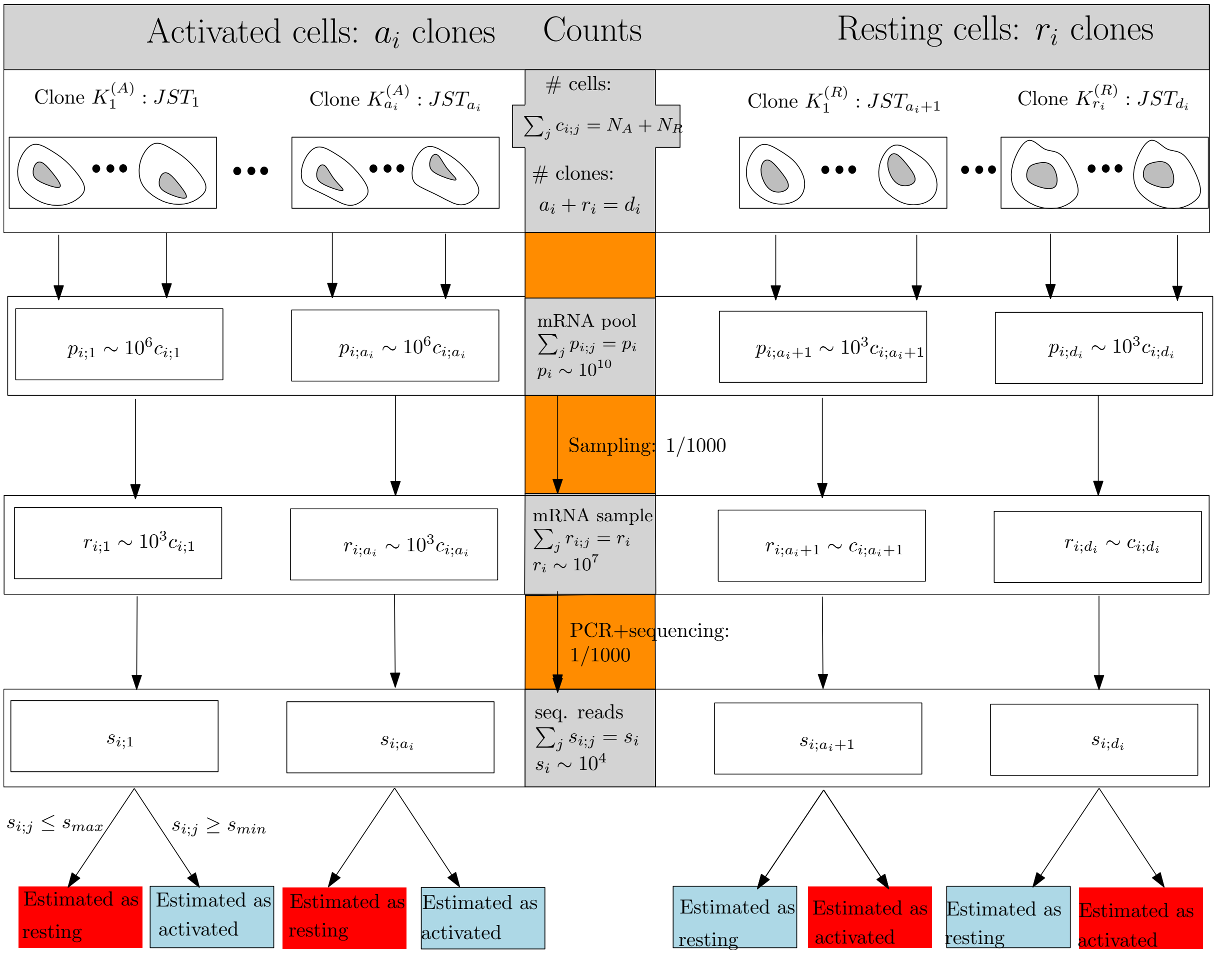

3 Connection to Hyper-geometric Distributions

Hyper-geometric distribution. Consider an urn containing N balls, M of which *count or matter*, and assume that n balls are drawn at random without replacement. The probability to obtain m balls which matter is given by the hyper-geometric distribution

$$P_H(N, M, n, m) = \frac{\binom{M}{m}\binom{N-M}{n-m}}{\binom{N}{n}}. \tag{6}$$

Urn models. In the sequel, we aim at modeling the process depicted on Fig. 1. To this end, we consider an urn containing n_A balls which matter, and n_B balls which do not matter, for a total of $n_A + n_B = p_i$ balls. The n_A balls correspond to the sequences of a specific clone, say the jth one, while the n_B balls correspond to the sequences of all the remaining clones. Numerical values shall be presented in section 6.2 for three types of urns.

Using this partition of all sequences, the first sampling step (which fills the urn of the intermediate layer from that of the second layer - i.e. from the p_i) is modeled by a hyper-geometric distribution whose outcome is denoted $R = (R_A, R_B)$. In particular, the probability to obtain at random $m = r_{i;j}$ mRNA molecules of type j is given by

$$P_H(n_A + n_B, n_A, r_i, m). \tag{7}$$

Similarly, the second sampling process (which fills the urn of the bottom layer from that of the intermediate layer) is modeled by a hyper-geometric distribution whose outcome is denoted $S = (S_A, S_B)$.

Consider the two random vectors R and S. Abusing notations, we shall write $P(R)$ for $P(R_A = r_A, R_B = r_B)$, and $P(S)$ for $P(S_A = s_A, S_B = s_B)$. By the law of total probability, the vectors R and S are coupled as follows:

$$P(S) = \binom{n_A}{s_A}\binom{n_B}{s_B}/\binom{n}{s} \tag{8}$$

$$\tag{9}$$

$$= \sum_R P(S \mid R)P(R) \tag{10}$$

$$\tag{11}$$

$$= \sum_R \frac{\binom{r_A}{s_A}\binom{r_B}{s_B}}{\binom{r}{s}} \frac{\binom{n_A}{r_A}\binom{n_B}{s_B}}{\binom{n}{r}} \tag{12}$$

These equations show that information on the bottom layer (the statistic S of Eq. (8)) can be fully inferred if all outcomes for the intermediate layers are known, which is important since one does not directly count sequences from the initial pool, as mentioned in section 2. Practically and due to the lack of direct information on these intermediate steps, we shall base our calculations only the top/second and bottom layers of Fig. 1, ignoring the potential bias related to all intermediate steps. As in almost all repertoire studies, this is based on the hypothesis that the first sampling steps - i.e. the cell/tissue preparation, RNA extraction and cDNA synthesis - are indeed perfumed at random and yield enough material to be robust. We are aware that this question should be taken into account for a full validation of a complete model, which is is beyond the scope of this article.

Tail inequalities for hyper-geometric distributions. An important quantity to be used to compute p-values is the weight of the tail of a hyper-geometric distribution, namely

$$\Sigma_H(N, M, n, k) = \sum_{m \geq k} P_H(N, M, n, m). \tag{13}$$

The lower bound $m \geq k$ together with the constraints imposed on the two binomial coefficients of Eq. (6) yields

$$\Sigma_H(N, M, n, k) = \sum_{m=max(k,n-N+M)}^{min(n,M)} P_H(N, M, n, m). \tag{14}$$

4 Statistical Tests at the JST Level for a Given Individual i

In this section, we instantiate the urn model just discussed and develop statistical tests. We use the term *"mRNA molecule* to refer to the content of the urn, which is sampled through the whole process, leading to sets of sequence reads that are aggregated into JSTs.

4.1 Outline

Consider a particular JST, whose type is j, so that its corresponding number of read counts is $s_{i;j}$ in the fish i. Our statistical test design follows the usual pattern, namely:

- A null hypothesis (H_0^- or H_0^+, see below) is formulated based on sequence read counts for the JST considered (i.e. $s_{i;j}$) and on the threshold $s_t = s_{min} = s_{max}$, using a hyper-geometric distribution associated with an urn model.
- The hyper-geometric distribution is used to compute a one-sided p-value, from which the null hypothesis assessed.

Urn model. We consider an urn containing a total of $N = n_A + n_B$ mRNA molecules, out of which $M = n_A$ matter. The fraction of mRNA molecules which matter is denoted $f_j^{(0)} = n_A/(n_A + n_B)$. The values of n_A and n_B shall depend on the values of $s_{i;j}$ and s_t, as detailed in the next two subsections.

Null hypothesis. We define *composite* null and alternative hypothesis associated with an hyper-geometric distribution defined by the (unknown) fraction of mRNA molecules which matter f_j. The hypothesis read as:

Definition. 1 (Null hypothesis H_0^-). *Consider a JST such that $s_{i;j} \leq s_t$. This JST presumably corresponds to a resting B cell clone, whence the null and alternative hypothesis:*

$$H_0^- : \ f_j \leq f_j^{(0)} \tag{15}$$

$$H_1^- : \ f_j > f_j^{(0)} \tag{16}$$

Definition. 2 (Null hypothesis H_0^+). *Consider a JST such that $s_{i;j} \geq s_t$. This JST presumably corresponds to an activated B cell clone, whence the null and alternative hypothesis:*

$$H_0^+ : \ f_j \geq f_j^{(0)} \tag{17}$$

$$H_1^+ : \ f_j < f_j^{(0)} \tag{18}$$

The mapping of JSTs to either activated and expanded B cell clones, or to resting B cells, which is based on counts related to the sampling process, faces two risks :

- Underestimation: in case of under-sampling, a JST produced by an activated amplified clone gets an erroneous *unique* label (see Eq. (4)), thus yielding an erroneous null hypothesis.
- Overestimation: in case of over-sampling, a JST produced by a resting clone gets an erroneous *repeated* label (see Eq. (5)), thus yielding an erroneous null hypothesis.

The case study is summarized on Fig. 2. In the following subsections, null hypotheses H_0^- and H_0^+ will be tested, based on the sampling process which consists of generating the vector $\mathbf{S}(O_i)$ from the vector $\mathbf{P}(O_i)$, using a hyper-geometric distribution.

Figure 2 Sequence status, sequence labels, and statistical null hypothesis. The sequence counts are used to assign labels to the sequences, which determine null hypothesis (H0).

Seq. status (unknown truth) / Seq. label (estimation)	From activated clone	From resting clone
Repeated ↳ H_0^+: the JST corresponds to an actived clone	H0 true	H0 false
Unique ↳ H_0^-: the JST corresponds to a resting clone	H0 false	H0 true

4.2 Testing $\mathbf{H_0^-}$

For H_0^-, we fill the urn of $n_A + n_B$ mRNA molecules as follows:

$$\begin{cases} n_A & = p_{i;j} \text{ mRNA molecules of type } j \\ n_B & = p_i - n_A \text{ mRNA molecules whose type is different from } j \end{cases} \tag{19}$$

The probability to draw exactly m mRNA molecules of type j is $P_H(n_A + n_B, n_A; s_i, m)$, and the corresponding one sided p-value associated with Eq. (15) is given by

$$\text{p-val}^-_{i;j} = \sum_{m \geq s_{i;j}} P_H(n_A + n_B, n_A, s_i, m) = \Sigma_H(p_i, p_{i;j}, s_i, s_{i;j}), \tag{20}$$

where s_i is the number of reads for the fish i, and $s_{i;j}$ is the number of reads of the tested JST_j.

Note that the one sided p-value decreases when $s_{i;j}$ increases.

4.3 Testing $\mathbf{H_0^+}$

For H_0^+, we fill the urn of $n_A + n_B$ mRNA molecules as follows:

$$\begin{cases} n_A & = p_{i;j} \text{ mRNA molecules of type } j \\ n_B & = p_i - n_A \text{ mRNA molecules whose type is different from } j \end{cases} \tag{21}$$

The probability to draw exactly m mRNA of type j is also given by Eq. (6), and the corresponding one sided p-value associated with Eq. (17) is given by

$$\text{p-val}^+_{i;j} = \sum_{m \leq s_{i;j}} P_H(n_A + n_B, n_A, s_i, m) = 1 - \Sigma_H(p_i, p_{i;j}, s_i, s_{i;j} + 1), \tag{22}$$

where s_i is the number of reads for the fish i, and $s_{i;j}$ is the number of reads of the tested JST_j.

Note that the one sided p-value increases when $s_{i;j}$ increases.

5 Implementation Note

The computation of the one sided p-values require the evaluation of large binomial coefficients. The implementation was carried out in python, using the `gmpy` library, which exposes to Python three number types implemented in the multi-precision arithmetic packages GMP and MPIR, namely mpz (unlimited-precision integers), mpq (unlimited-precision rationals), and mpf (extended-precision floats). See `http://code.google.com/p/gmpy/` for more details. One thousand digits were used as precision for the floating point numbers mpf.

The python script can be obtained from F. Cazals (Frederic.Cazals@inria.fr).

6 Assessing Public and Private Responses from Observed JSTs

6.1 Method

Consider two sets of infected and control fish, respectively.

A public response corresponds to a clonal expansion (or a set of clonal expansions expressing highly similar junctions with presumably the same epitope specificity), which is present in all infected individuals sharing the same genetic background; hence, these junctions are present but expressed at low frequency by resting clones in all naive individuals. The probability to find them in the sequence datasets of control fish is therefore very close to 0. The corresponding JST (respectively JST set) is repeated in all the infected fish of the study, and absent in the datasets produced from the control ones. Similarly, a private response corresponds to a clonal expansion which is present in a given (or *only a few*) infected individuals sharing the same genetic background; the corresponding JST is therefore repeated in this infected fish, and absent in the datasets produced from the other infected fish or from the control ones.

6.2 Filling the urns

To compute the p-values of Eq. (20) and (22), the compositions of the urns must be defined. We actually define common parameters, together with three different urn models.

Common parameters. Consider the case of a control fish.

- We assume that its spleen contains $3-4\times 10^8$ leucocytes , 30-50% of which - i.e. $1.5\ 10^8$ - are B cells. Taking into account the relative frequency of IgM^+ and IgT^+ B cells in the spleen, there are:

$$112\times 10^6\ \mathrm{IgM}^+\mathrm{B\ cells\ and}\ 38\times 10^6\ \mathrm{IgT}^+\mathrm{B\ cells},$$

- Considering arbitrarily that 10 different VH families are equally represented in the spleen B cell population, we assume arbitrarily that 10% of B cells express a particular VH_k family.

  The numbers of B cells relevant for an urn - i.e. for a given VH/C combination - are therefore:

$$112\times 10^5\ \mathrm{IgM}^+\mathrm{B\ cells\ and}\ 38\times 10^5\ \mathrm{IgT}^+\mathrm{B\ cells}$$

- Estimated Number of cells of the jth clone:
  - If the clone is resting, $c_{i;j}=10$
  - If the clone is activated, we assume $c_{i;j}=1800$. In fact, assuming that a B cell undergoes 5-10 successive divisions [CH07, THH08] after activation - which are not necessarily synchronous- the size of such a clone can be estimated to $2^{7.5}\times 10\sim 1800$ cells.
- $s_{min}=s_{max}=10$ is used to define unique and repeated sequences.

The default urn of a control fish. We consider that the typical size of a resting clone is of $c_{i;j}=10$ cells, and that a resting cell from such a clone contains circa 10^3 mRNA [MG84]. One considers that B cell from an activated clone produces 1000 more mRNA than a resting B cells [KP86, MKZ94]. One also considers that the frequency of activated B cells is around .001 among B cells of control individuals.

Overall, we therefore assume that the whole population of B cells corresponding to a given VH/C combination contains

$$p_{\mathrm{ctrl}}=\ \text{Nb of resting B cells }\times 10^3+\text{Nb of activated B cells }\times 10^6\ \text{IgH mRNA}\tag{23}$$

Hence,

- for IgM^+ B cells, considering that the frequency of activated B cells is around 0.001,

$$p_{\mathrm{ctrl}}=112\ 10^5\times 10^3+112\ 10^2\times 10^6,\tag{24}$$

- for IgT$^+$ B cells, considering that the frequency of activated B cells is around 0.001,

$$p_{\text{ctrl}} = 38\ 10^5 \times 10^3 + 38\ 10^2 \times 10^6, \tag{25}$$

Hence, we shall consider that $p_{\text{ctrl}} = 2.25\ 10^{10}$ for IgM and $p_{\text{ctrl}} = 0.75\ 10^{10}$ for IgT.

Urn in which one JST is tested in an infected fish (Null hypothesis= $\mathbf{H}_0^+$) . Such an urn is defined by adding the number of mRNA produced by the clone(s) encoding the tested JST(s) to the number predicted for a control fish i.e. to p_{ctrl}.

The size of an activated clone is set t $c_{i;j} = 1800$ cells, whence the following urn composition:

- for IgM$^+$ B cells: $p_{i;j} = 1800\ 10^6,\ p_i = p_{\text{ctrl}} + p_{i;j} = 2.25\ 10^{10} + 1800\ 10^6 = 2.42\ 10^{10}$.
- for IgT$^+$ B cells: $p_{i;j} = 1800\ 10^6,\ p_i = p_{\text{ctrl}} + p_{i;j} = 0.75\ 10^{10} + 1800\ 10^6 = 0.92\ 10^{10}$.

Urn in which a set of k JST are tested.

- For H_0^+ tested in an infected fish, the size of a clone is taken as $c_{i;j} = k \times 1800$, whence the following urn composition:
  - for IgM$^+$ B cells: $p_{i;j} = k \times 1800 \times 10^6,\ p_i = p_{\text{ctrl}} + p_{i;j} = 2.25\ 10^{10} + k \times 1800 \times 10^6$
  - for IgT$^+$ B cells: $p_{i;j} = k \times 1800 \times 10^6,\ p_i = p_{\text{ctrl}} + p_{i;j} = 0.75\ 10^{10} + k \times 1800 \times 10^6$.

  Since k clones are involved, the total number of reads corresponding to the k JSTs tested is $\sum_{t=1,\ldots,k} s_{i;j_t}$

- For H_0^- tested in a control fish, the size of a clone is taken an $c_{i;j} = k \times 10$, whence the following urn composition:
  - for IgM$^+$ B cells: $p_{i;j} = k \times 10 \times 10^3,\ p_i = p_{\text{ctrl}} = 2.25\ 10^{10} + p_{i;j}$
  - for IgT$^+$ B cells: $p_{i;j} = k \times 10 \times 10^3,\ p_i = p_{\text{ctrl}} = 0.75\ 10^{10} + p_{i;j}$

  As above, the total number of reads corresponding to the k JSTs tested is $\sum_{t=1,\ldots,k} s_{i;j_t}$.

6.3 One-sided p-values curves for increasing values of the number of sequences $s_{i;j}$ observed for the jth clone

Figure 3 Testing the null hypothesis for an activated clone in an infected fish. The values computed use $p_i = 10^{10}$ and a number of cells per activated clone $c_{i;j} = 1800$.

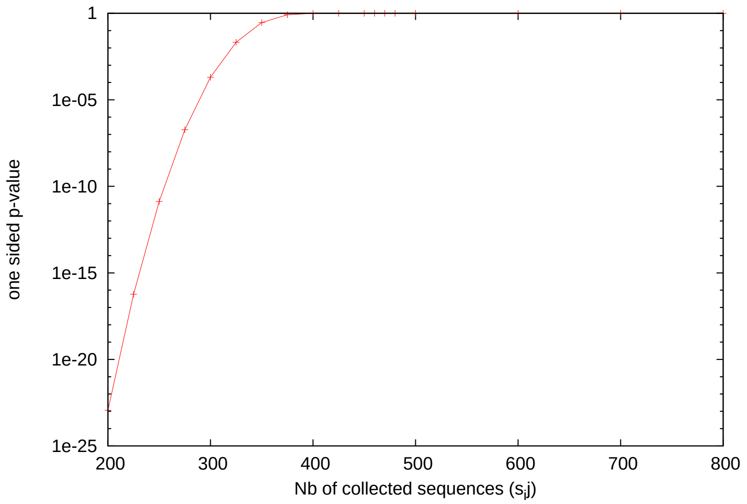

Figure 4 Testing the null hypothesis for a resting clone. The values computed use $p_i = 10^{10}$ and a number of cells per activated clone $c_{i;j} = 10$. Note that the null hypothesis (the clone is resting) is rejected if $s_{i;j} > 0$.

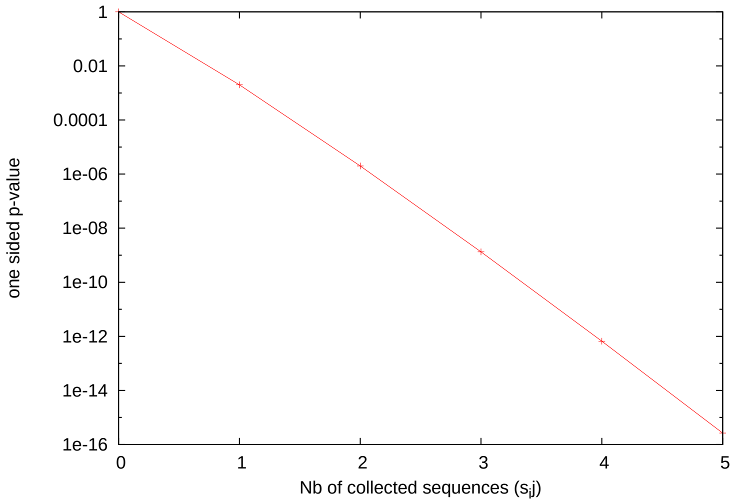

6.4 Results

In the following, the null hypothesis have been assessed at the significance level 0.05.

6.4.1 Does a large JST found in one clone support H_0^+?

Under our model, the null hypothesis H_0^+ was not rejected for one or two repeated JSTs per fish for VH4Cmu, VH4Ctau or VH5.1Cmu, i.e. for those with most reads (see Table 1 below). This observation is consistent with the trend shown on figure 3A.

6.4.2 The case of private responses

A private response is assumed for a JST significantly amplified in one infected fish A, and not present in other infected fish, or in control fish. Several such private responses were validated in our model, and are listed in the first section of the Table 1 (see below). For each junction, the table reads as follows:

A In the fish A, H_0^+ is not rejected, and we conclude that the B cell clone expressing the JST_j is indeed activated in this fish.

B In other infected fish, H_0^+ is rejected.

C The JST_j being ABSENT in the other infected animals ($s_{i;j} = 0$), H_0^- is not rejected in these individuals.

6.4.3 The case of public responses

We consider here a public response as a unique JST_j (or a set of closely related JSTs differing by conservative substitutions and presumably targeting the same epitope as described in public responses in mammals) : at least one of these $JST_{j_1 \ldots j_k}$ is represented by repeated sequences in all infected individuals, and NONE of these $JST_{j_1 \ldots j_k}$ is found in any control fish. The corresponding tests are presented in the last two sections of the Table 2 (see below). First, it is shown that several VH51/J5 JSTs are found in several infected fish in which H_0^+ is not rejected. Additionally, taking the related VH51/J5 JSTs repeated in several fish as a whole (in the section "Public response of the Table 1), it appears that H_0^+ cannot be rejected in infected fish, and that H_0^- is not rejected in control fish.

We conclude that our data support the idea that B cell clones expressing the $\mathrm{JST}_{j_1 \ldots j_k}$ are indeed activated in these fish and are present in each infected fish, while $\mathrm{JST}_{j_1 \ldots j_k}$ are expressed by resting clones in control fish.

It is important to note that if even only one of the $\mathrm{JST}_{j_1 \ldots j_k}$ would have been detected even in only one of the control fish, H0 would be rejected (Fig. 4), and the set of JST could not be considered as expressed by a public response any more.

	H0	s_i	$s_{i;j}$	p-val
Private responses				
JST VH4J3Cmu ARANNVIFSFDY				
Fish4(inf)	H_0^+	3424	0	3.61e-115 Rejected
Fish5(inf)	H_0^+	1822	0	1.26e-61 Rejected
Fish6(inf)	H_0^+	3290	0	1.09e-110 Rejected
Fish7(inf)	H_0^+	2465	640	1.00e+00 Not rejected
Fish1(Ctrl)	H_0^+	2188	0	7.39e-74 Rejected
Fish2(Ctrl)	H_0^+	2171	0	2.74e-73 Rejected
Fish3(Ctrl)	H_0^+	1965	0	2.10e-66 Rejected
JST VH4J5Cmu ARGRYTLGASAFDY				
Fish4(inf)	H_0^+	3424	0	3.61e-115 Rejected
Fish5(inf)	H_0^+	1822	0	1.26e-61 Rejected
Fish6(inf)	H_0^+	3290	0	1.09e-110 Rejected
Fish7(inf)	H_0^+	2465	302	1.00e+00 Not rejected
Fish1(Ctrl)	H_0^+	2188	0	7.39e-74 Rejected
Fish2(Ctrl)	H_0^+	2171	0	2.74e-73 Rejected
Fish3(Ctrl)	H_0^+	1965	0	2.10e-66 Rejected
JST VH4J5Cmu ARERRSGGAFDY				
Fish4(inf)	H_0^+	3424	0	3.61e-115 Rejected
Fish5(inf)	H_0^+	1822	0	1.26e-61 Rejected
Fish6(inf)	H_0^+	3290	947	1.00e+00 Not rejected
Fish7(inf)	H_0^+	2465	0	4.08e-83 Rejected
Fish1(Ctrl)	H_0^+	2188	0	7.39e-74 Rejected
Fish2(Ctrl)	H_0^+	2171	0	2.74e-73 Rejected
Fish3(Ctrl)	H_0^+	1965	0	2.10e-66 Rejected
JST VH4J7Cmu AREGGATPDRSYAAFDS				
Fish4(inf)	H_0^+	3424	0	3.61e-115 Rejected
Fish5(inf)	H_0^+	1822	348	1.00e+00 Not rejected
Fish6(inf)	H_0^+	3290	0	1.09e-110 Rejected
Fish7(inf)	H_0^+	2465	0	4.08e-83 Rejected
Fish1(Ctrl)	H_0^+	2188	0	7.39e-74 Rejected
Fish2(Ctrl)	H_0^+	2171	0	2.74e-73 Rejected
Fish3(Ctrl)	H_0^+	1965	0	2.10e-66 Rejected
JST VH4J7Cmu AREASLRGSYAAFDS				
Fish4(inf)	H_0^+	3424	680	1.00e+00 Not rejectedd
Fish5(inf)	H_0^+	1822	0	1.26e-61 Rejected
Fish6(inf)	H_0^+	3290	0	1.09e-110 Rejected
Fish7(inf)	H_0^+	2465	0	4.08e-83 Rejected
Fish1(Ctrl)	H_0^+	2188	0	7.39e-74 Rejected
Fish2(Ctrl)	H_0^+	2171	0	2.74e-73Rejected
Fish3(Ctrl)	H_0^+	1965	0	2.10e-66 Rejected
JST VH4J7Cmu ARDGLYTGRSYAAFDS				
Fish4(inf)	H_0^+	3424	0	3.61e-115 Rejected
Fish5(inf)	H_0^+	1822	0	1.26e-61 Rejected
Fish6(inf)	H_0^+	3290	818	1.00e+00 Not rejected
Fish7(inf)	H_0^+	2465	0	4.08e-83 Rejected
Fish1(Ctrl)	H_0^+	2188	0	7.39e-74 Rejected
Fish2(Ctrl)	H_0^+	2171	0	2.74e-73 Rejected
Fish3(Ctrl)	H_0^+	1965	0	2.10e-66 Rejected
JST VH4J1Ctau ARERGRSTVTVWAFYSRGAFDY				
Fish4(inf)	H_0^+	3207	761	1.00e+00 Not rejected
Fish5(inf)	H_0^+	2070	0	4.14e-194 Rejected
Fish6(inf)	H_0^+	2823	0	1.86e-264 Rejected
Fish7(inf)	H_0^+	2936	0	5.17e-275 Rejected
Fish3(Ctrl)	H_0^+	1590	0	2.88e-149 Rejected
JST VH4J2Ctau AREGVYSYSLGYFDY				
Fish4(inf)	H_0^+	3207	0	3.22e-62 Rejected

Fish5(inf)	H_0^+	2070	1153	1.00e+00 Not rejected
Fish6(inf)	H_0^+	2823	0	1.86e-264 Rejected
Fish7(inf)	H_0^+	2936	0	5.17e-275 Rejected
Fish3Ctrl)	H_0^+	1590	0	2.88e-149 Rejected
JST VH4J1Ctau ARDIQLQFGLFIHAFDY				
Fish4(inf)	H_0^+	3207	0	3.22e-62 Rejected
Fish5(inf)	H_0^+	2070	0	4.14e-194 Rejected
Fish6(inf)	H_0^+	2823	864	1.00e+00 Not rejected
Fish7(inf)	H_0^+	2936	0	5.17e-275 Rejected
Fish3(Ctrl)	H_0^+	1590	0	2.88e-149 Rejected
JST VH4J1Ctau ARGYTVTVWAFYAFDY				
Fish4(inf)	H_0^+	3207	0	3.22e-62 Rejected
Fish5(inf)	H_0^+	2070	0	4.14e-194 Rejected
Fish6(inf)	H_0^+	2823	0	1.86e-264 Rejected
Fish7(inf)	H_0^+	2936	808	1.00e+00 Not rejected
Fish3(Ctrl)	H_0^+	1590	0	2.88e-149 Rejected
JSTs VH51Cmu found in several fish				
(tested one by one)				
JST VH51J5Cmu ARYNGDAFDY (found in several fish)add				
Fish4(inf)	H_0^+	2153	626	1.00e+00 Not rejected
Fish5(inf)	H_0^+	2067	0	8.19e-70 Rejected
Fish6(inf)	H_0^+	2748	166	2.80e-03 Rejected
Fish7(inf)	H_0^+	2291	0	2.67e-77 Rejected
Fish1(Ctrl)	H_0^+	2039	0	7.06e-69 Rejected
Fish2(Ctrl)	H_0^+	2324	0	2.10e-78 Rejected
Fish3(Ctrl)	H_0^+	2003	0	1.13e-67 Rejected
JST VH51J5Cmu ARYDNNAFDY (found in several fish)add				
Fish4(inf)	H_0^+	2153	0	1.09e-72 Rejected
Fish5(inf)	H_0^+	2067	209	1.00e+00 Not rejected
Fish6(inf)	H_0^+	2748	0	1.42e-92 rejected
Fish7(inf)	H_0^+	2291	169	4.98e-01 Not rejected
Fish1(Ctrl)	H_0^+	2039	0	7.06e-69 Rejected
Fish2(Ctrl)	H_0^+	2324	0	2.10e-78 Rejected
Fish3(Ctrl)	H_0^+	2003	0	1.13e-67 Rejected
JST VH51J5Cmu ARYNNNAFDY (found in several fish)				
Fish4(inf)	H_0^+	2153	0	1.09e-72 Rejected
Fish5(inf)	H_0^+	2067	264	1.00e+00 Not rejected
Fish6(inf)	H_0^+	2748	336	1.00e+00 Not rejected
Fish7(inf)	H_0^+	2291	347	1.00e+00 Not rejected
Fish1(Ctrl)	H_0^+	2039	0	7.06e-69 Rejected
Fish2(Ctrl)	H_0^+	2324	0	2.10e-78 Rejected
Fish3(Ctrl)	H_0^+	2003	0	1.13e-67 Rejected
JST VH51J5Cmu ARYSGDAFDY (found in several fish)add				
Fish4(inf)	H_0^+	2153	34	1.09e-72 Rejected
Fish5(inf)	H_0^+	2067	0	8.19e-70 Rejected
Fish6(inf)	H_0^+	2748	322	1.00e+00 Not rejected
Fish7(inf)	H_0^+	2291	0	2.67e-77 Rejected
Fish1(Ctrl)	H_0^+	2039	0	7.06e-69 Rejected
Fish2(Ctrl)	H_0^+	2324	0	2.10e-78 Rejected
Fish3(Ctrl)	H_0^+	2003	0	1.13e-67 Rejected
Public response VH51J5Cmu (related JST found in several fish)				
JSTs: ARYNNNAFDY;ARYDNNAFDY; ARYNSNAFDY;				
ARYNNDAFDY;ARYSGDAFDY; ARYNGDAFDY, taken as a whole				
Fish4(inf)	H_0^+	2153	660	1.00e+00 Not rejected
Fish5(inf)	H_0^+	2067	499	1.00e+00 Not rejected
Fish6(inf)	H_0^+	2748	969	9.99e-01 Not rejected
Fish7(inf)	H_0^+	2291	799	1.00e+00 Not rejected

Fish1(Ctrl)	H_0^+	2039	0	1.46e-74	Rejected
Fish2(Ctrl)	H_0^+	2324	0	6.96e-85	Rejected
Fish3(Ctrl)	H_0^+	2003	0	2.93e-73	Rejected
Fish4(inf)	H_0^-	2153	660	0.00e+00	Rejected
Fish5(inf)	H_0^-	2067	499	0.00e+00	Rejected
Fish6(inf)	H_0^-	2748	969	0.00e+00	Rejected
Fish7(inf)	H_0^-	2291	799	0.00e+00	Rejected
Fish1(Ctrl)	H_0^-	2039	0	1.00e+00	Not rejected
Fish2(Ctrl)	H_0^-	2324	0	1.00e+00	Not rejected
Fish3(Ctrl)	H_0^-	2003	0	1.00e+00	Not rejected

Table 1: **Results : testing $\mathbf{H_0^+}$ and $\mathbf{H_0^-}$.** Note that datasets for which the total number of read was less than 1500 were not used.

References

[CH07] R. Callard and P. Hodgkin. Modeling T-and B-cell growth and differentiation. *Immunological reviews*, 216(1):119–129, 2007.

[KP86] D.E. Kelley and R.P. Perry. Transcriptional and posttranscriptional control of immunoglobulin mRNA production during B lymphocyte development. *Nucleic acids research*, 14(13):5431–5447, 1986.

[MG84] T. Manser and M.L. Gefter. Isolation of hybridomas expressing a specific heavy chain variable region gene segment by using a screening technique that detects mrna sequences in whole cell lysates. *Proceedings of the National Academy of Sciences*, 81(8):2470, 1984.

[MKZ94] T. Matthes, V. Kindler, and R.H. Zuber. Semiquantitative, nonradioactive RT-PCR detection of immunoglobulin mRNA in human b cells and plasma cells. *DNA and cell biology*, 13(4):429–436, 1994.

[THH08] M.L. Turner, E.D. Hawkins, and P.D. Hodgkin. Quantitative regulation of B cell division destiny by signal strength. *The Journal of Immunology*, 181(1):374–382, 2008.
