# Supplementary material for: Teleost Fish Mount Complex Clonal IgM and IgT Responses in Spleen upon Systemic Viral Infection
Source: PLoS Pathog. 2013 Jan 10;9(1):e1003098. doi: 10.1371/journal.ppat.1003098 (PMC3542120; doi:10.1371/journal.ppat.1003098)

Figure S10. Pajek representations of IgM and IgT repertoires.
Number reads with productive junctions: VH4Cμ control = 1428, 1441, 1238; VH4Cμ infected = 2213, 1212, 2603, 1727; VH4Cτ control =1027; VH4Cτ infected = 1349, 2163, 1964, 2052; VH5.1Cμ control = 1403, 1288, 1572 ; VH5.1Cμ infected = 1446, 1403, 1863, 1537; VH5.1Cτ control = 1202, 791; VH5.1Cτ infected = 1344, 983, 1044, 1073; VH5.4Cμ control = 711, 422, 1961; VH5.4Cμ infected = 1262, 1839, 3023, 1439; VH5.4Cτ control = 899, 334, 2240; VH5.4Cτ infected = 1867, 1827, 1776, 1337; VH1.1Cμ control = 1663, 535, 1901; VH1.1Cμ infected = 2872, 2018, 804). Pajek representations are given only for datasets comprising more than 300 reads.

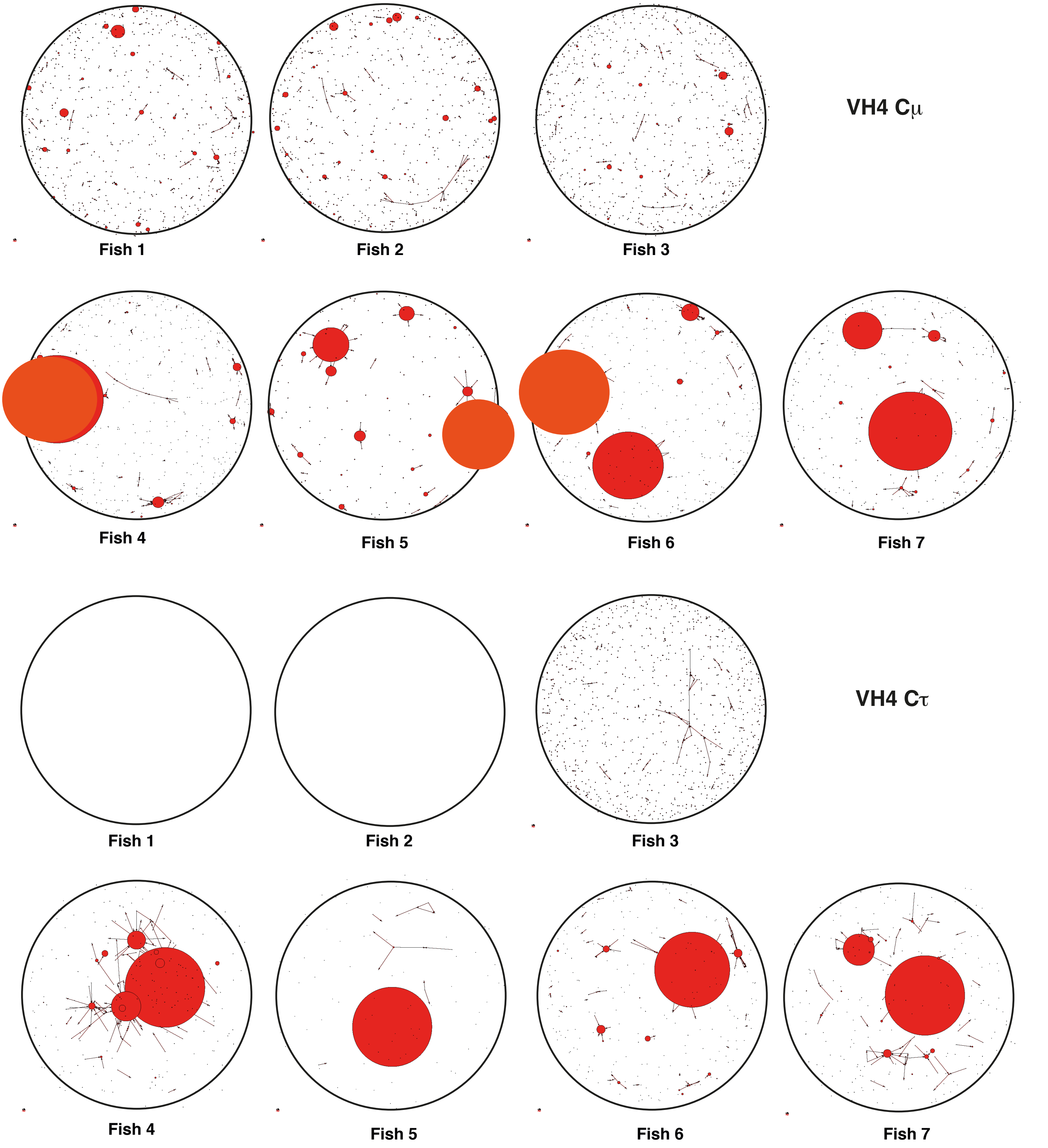

Figure S10. Pajek representations of IgM and IgT repertoires.

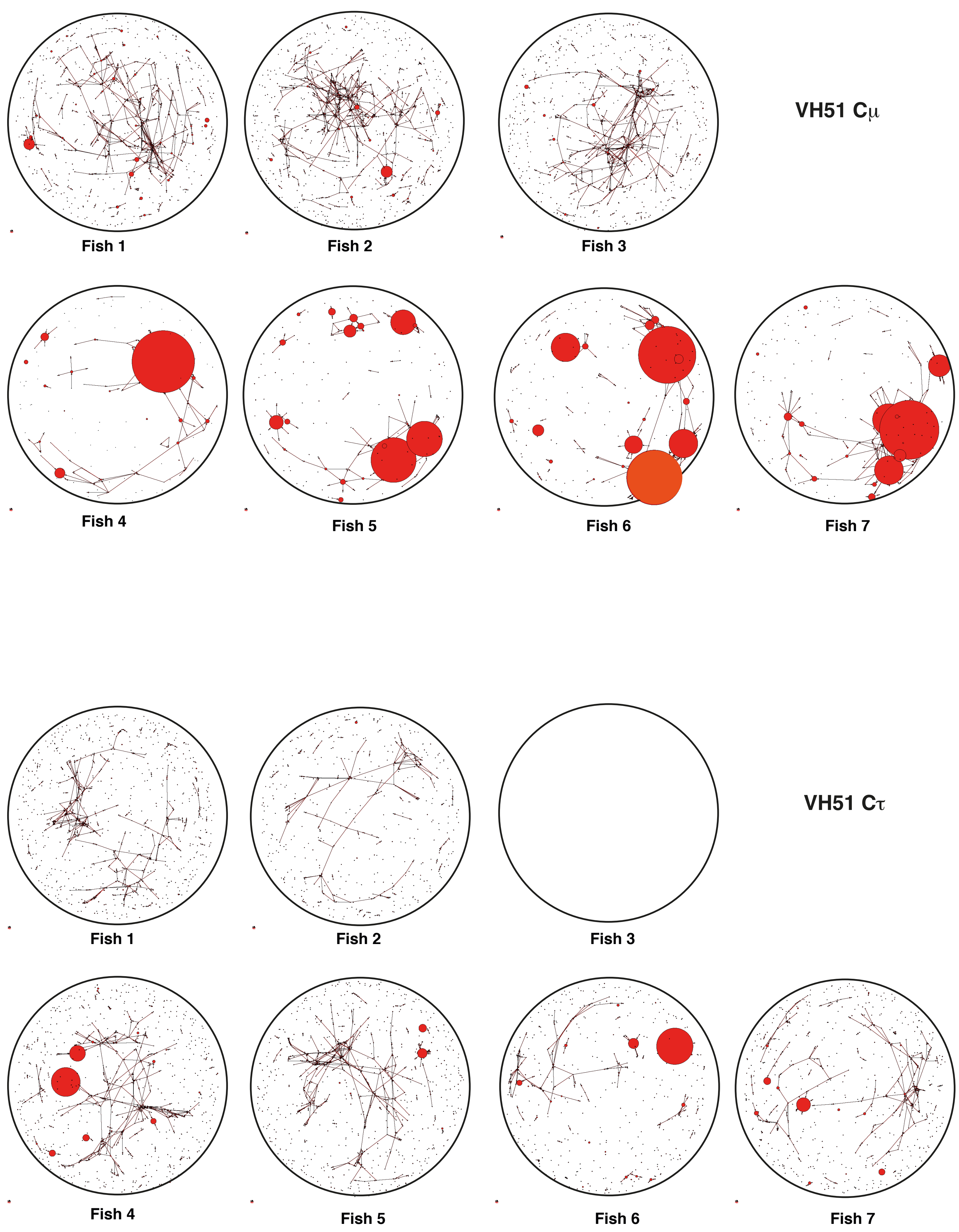

Figure S10. Pajek representations of IgM and IgT repertoires.

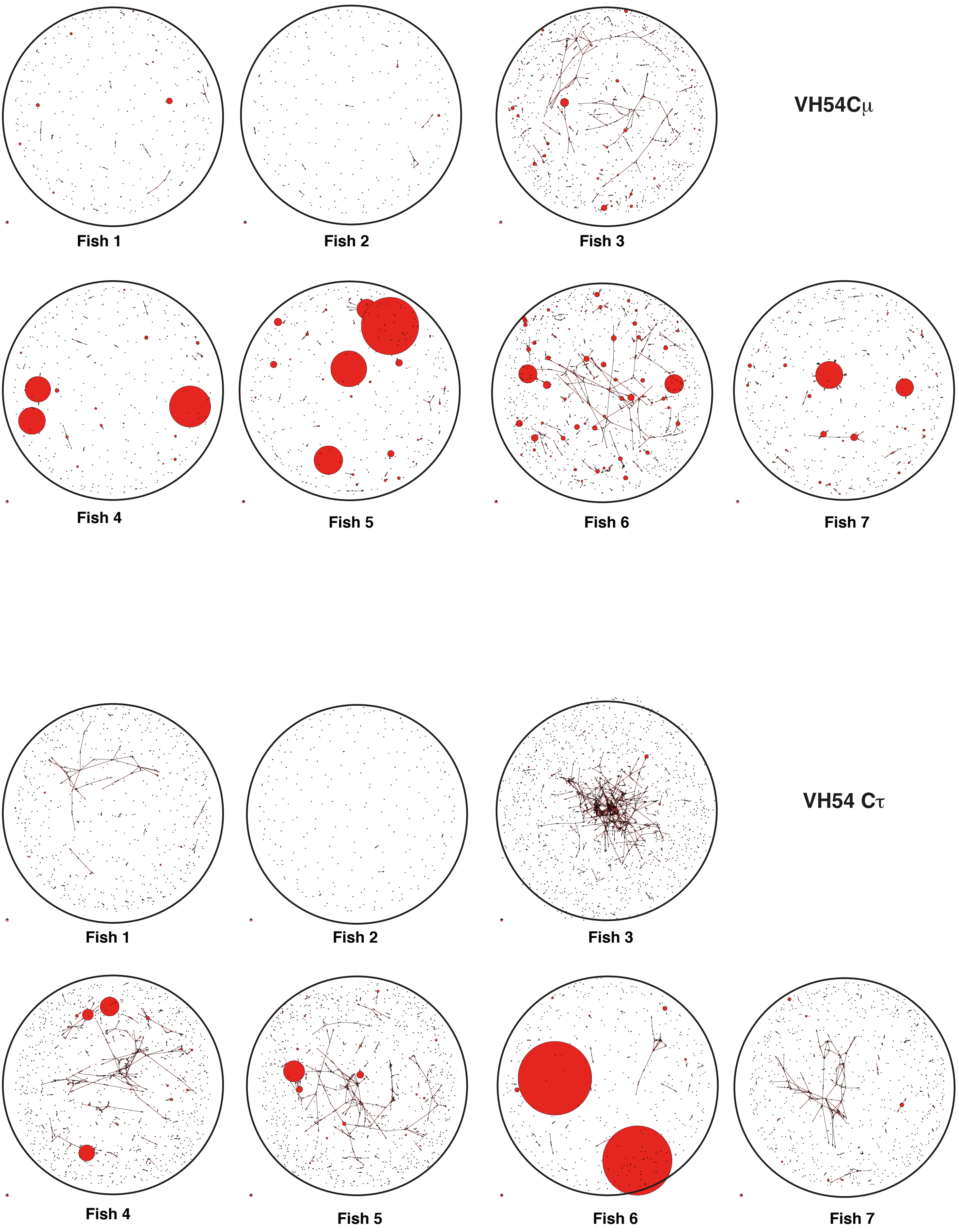

Figure S10. Pajek representations of IgM and IgT repertoires.

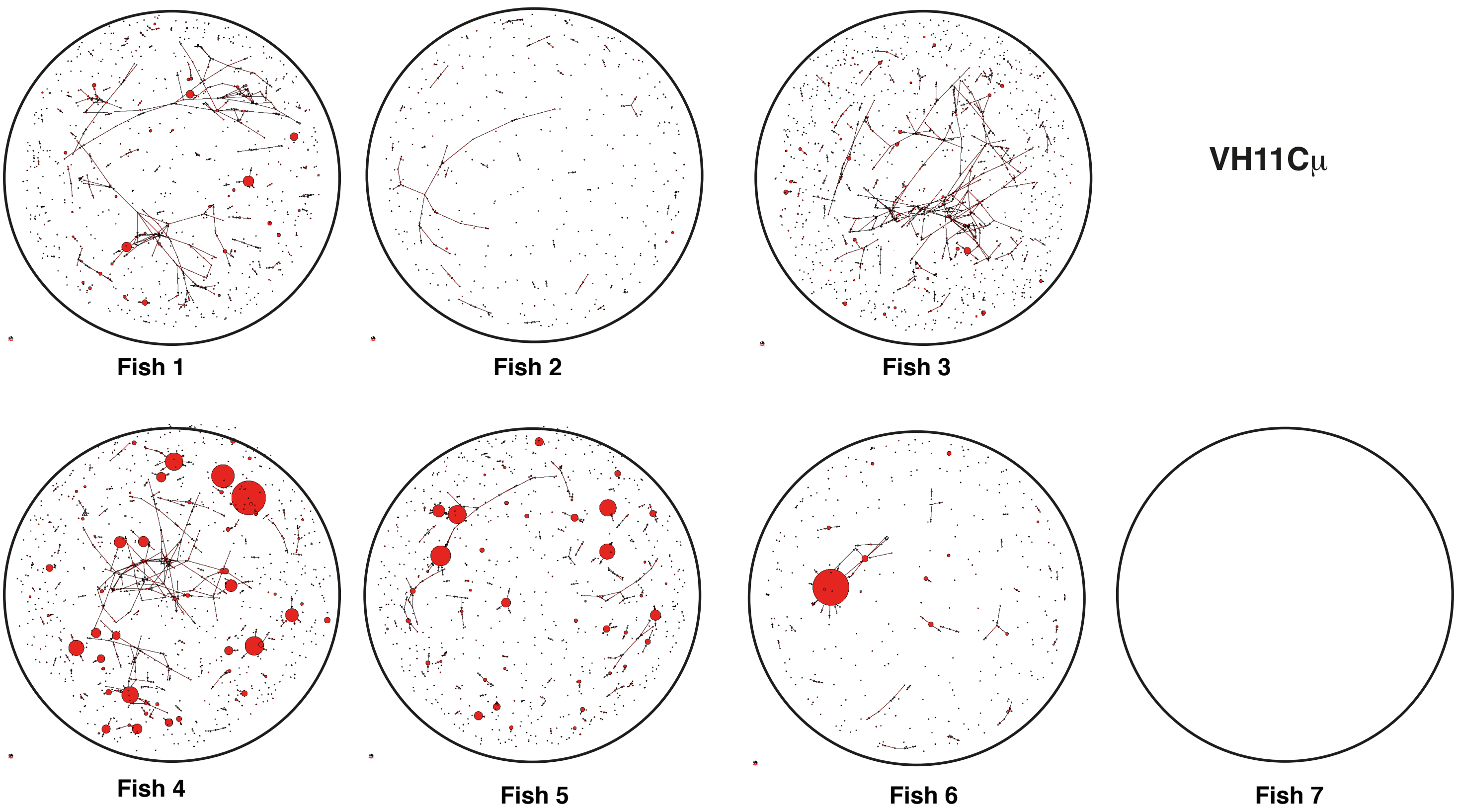

Supplement: Figure S10 — Pajek representations of IgM and IgT repertoires. (PDF) [file ppat.1003098.s010.pdf]
