# Supplementary material for: Teleost Fish Mount Complex Clonal IgM and IgT Responses in Spleen upon Systemic Viral Infection
Source: PLoS Pathog. 2013 Jan 10;9(1):e1003098. doi: 10.1371/journal.ppat.1003098 (PMC3542120; doi:10.1371/journal.ppat.1003098)
Supplement: Figure S11 — JST encoded by different nucleotide sequences. (PDF) [file ppat.1003098.s011.pdf]

Figure S11   JST encoded by different nucleotide sequences

JST encoded by different nucleotide sequences (total read number >50)

VH4-Ctau

Fish #	V-Gene	J-Gene	AA	#unique NT	NT	# sequences	
7	IGHV4S1*01	IGHJ1*01	ARGYTVTVWAFYAFDY	7	gctcgaggctatacagttacagtttgggctttttatgcttttgactac	**20**	
7	IGHV4S1*01	IGHJ1*01	ARGYTVTVWAFYAFDY	7	gctcgagggtatacagttacagtttgggctttttatgcttttgactac	**108**	
7	IGHV4S1*01	IGHJ1*01	ARGYTVTVWAFYAFDY	7	gctcgagggtatacagttacagtttgggctttttatgcttttgactat	1	Presumably technical arrtifact
7	IGHV4S1*01	IGHJ1*01	ARGYTVTVWAFYAFDY	7	gctcgagggtatacagttacggtttgggctttttatgcttttgactac	1	Presumably technical arrtifact
7	IGHV4S1*01	IGHJ1*01	ARGYTVTVWAFYAFDY	7	gcccgagggtatacagttacagtttgggctttttatgcttttgactac	1	Presumably technical arrtifact
7	IGHV4S1*01	IGHJ1*01	ARGYTVTVWAFYAFDY	7	gctcgagggtatacggttacagtttgggctttttatgcttttgactac	1	Presumably technical arrtifact
7	IGHV4S1*01	IGHJ1*01	ARGYTVTVWAFYAFDY	7	gctcggggatatacagttacagtttgggctttttatgcttttgactac	1	Presumably technical arrtifact
7	IGHV4S1*01	IGHJ1*01	ARGYTVTVWAFYAFDY	7	all	133	0.18796992481203

	V-Gene	J-Gene	AA	#unique NT	NT	# sequences	% minorities/majority
4	IGHV4S1*01	IGHJ1*01	ARDIQLQFGLFIRSFDY	2	gctcgagatatacagttacagtttgggctttttattcgatcttttgacta	**11**	
4	IGHV4S1*01	IGHJ1*01	ARDIQLQFGLFIRSFDY	2	gctcgagatatacagttacagtttgggctttttattcgaagttttgacta	**47**	
4	IGHV4S1*01	IGHJ1*01	ARDIQLQFGLFIRSFDY	2	all	58	0.189655172413793

7	IGHV4S1*01	IGHJ1*01	ARGYTVTVWAFYAFDY	18	gctcgaggctatacagttacagtttgggctttttatgcttttgactac	**116**	type 1
7	IGHV4S1*01	IGHJ1*01	ARGYTVTVWAFYAFDY	18	gctcgagggtatacagttacagtttgggctttttatgcttttgactac	**669**	type 2
7	IGHV4S1*01	IGHJ1*01	ARGYTVTVWAFYAFDY	18	gctcgcggctatacagttacagtttgggctttttatgcttttgactac	1	Presumably technical arrtifact
7	IGHV4S1*01	IGHJ1*01	ARGYTVTVWAFYAFDY	18	gctcgagggtatacagttacggtttgggctttttatgcttttgactac	2	Presumably technical arrtifact
7	IGHV4S1*01	IGHJ1*01	ARGYTVTVWAFYAFDY	18	gctcgagggtatacagttacagtttgggctttttacgcttttgactac	1	Presumably technical arrtifact
7	IGHV4S1*01	IGHJ1*01	ARGYTVTVWAFYAFDY	18	gctcgagggtatacagttaccgtttgggctttttatgcttttgactac	1	Presumably technical arrtifact
7	IGHV4S1*01	IGHJ1*01	ARGYTVTVWAFYAFDY	18	gctcgagggtatacagttacagtttgggctttttatgctttcgactac	1	Presumably technical arrtifact
7	IGHV4S1*01	IGHJ1*01	ARGYTVTVWAFYAFDY	18	gctcgaggatatacagttacagtttgggctttttatgcttttgactac	4	Presumably technical arrtifact
7	IGHV4S1*01	IGHJ1*01	ARGYTVTVWAFYAFDY	18	gctcgagggtatacagttacagtttgggccttttatgcttttgactac	2	Presumably technical arrtifact
7	IGHV4S1*01	IGHJ1*01	ARGYTVTVWAFYAFDY	18	gctcgggggtatacagttacagtttgggctttttatgcttttgactac	1	Presumably technical arrtifact
7	IGHV4S1*01	IGHJ1*01	ARGYTVTVWAFYAFDY	18	gcccgagggtatacagttacagtttgggctttttatgcttttgactac	3	Presumably technical arrtifact
7	IGHV4S1*01	IGHJ1*01	ARGYTVTVWAFYAFDY	18	gctcgagggtatacagttacagtctgggctttttatgcttttgactac	1	Presumably technical arrtifact
7	IGHV4S1*01	IGHJ1*01	ARGYTVTVWAFYAFDY	18	gctcggggctatacagttacagtttgggctttttatgcttttgactac	1	Presumably technical arrtifact
7	IGHV4S1*01	IGHJ1*01	ARGYTVTVWAFYAFDY	18	gctcgtggctatacagttacagtttgggctttttatgcttttgactac	1	Presumably technical arrtifact
7	IGHV4S1*01	IGHJ1*01	ARGYTVTVWAFYAFDY	18	gctcgagggtatacggttacagtttgggctttttatgcttttgactac	2	Presumably technical arrtifact
7	IGHV4S1*01	IGHJ1*01	ARGYTVTVWAFYAFDY	18	gctcgagggtacacagttacagtttgggctttttatgcttttgactac	3	Presumably technical arrtifact
7	IGHV4S1*01	IGHJ1*01	ARGYTVTVWAFYAFDY	18	gctcgagggtatacagttacagtttgggctttttatgcctttgactac	4	Presumably technical arrtifact
7	IGHV4S1*01	IGHJ1*01	ARGYTVTVWAFYAFDY	18	gctcgagggtatacagttacagtttgggctttctatgcttttgactac	2	Presumably technical arrtifact
7	IGHV4S1*01	IGHJ1*01	ARGYTVTVWAFYAFDY	18	all	815	0.179141104294479
**This JST has been found in another infected fish (#4), encoded by one of the most frequent nucleotide junction in fish#7(type 1)**							
4	IGHV4S1*01	IGHJ1*01	ARGYTVTVWAFYAFDY	1	gctcgaggctatacagttacagtttgggctttttatgcttttgactac	**2**	type 1

JST encoded by different nucleotide sequences (total read number <50)

VH1-Cmu

	V-Gene	J-Gene	AA	#unique NT	NT	# sequences	% minorities/majority
4	IGHV1S1*01	IGHJ5*01	ARDNYNAFDY	6	gccagagataactacaatgcttttgactac	26	
4	IGHV1S1*01	IGHJ5*01	ARDNYNAFDY	6	gccagagataactacaatgcctttgactac	2	
4	IGHV1S1*01	IGHJ5*01	ARDNYNAFDY	6	gccagagacaactacaatgcttttgactac	1	
4	IGHV1S1*01	IGHJ5*01	ARDNYNAFDY	6	gccagagataactacaacgcttttgactac	1	
4	IGHV1S1*01	IGHJ5*01	ARDNYNAFDY	6	gccagagataactacaatgctttcgactac	1	
4	IGHV1S1*01	IGHJ5*01	ARDNYNAFDY	6	gccagggataactacaatgcttttgactac	1	
4	IGHV1S1*01	IGHJ5*01	ARDNYNAFDY	6	all	32	0.1875

	V-Gene	J-Gene	AA	#unique NT	NT	# sequences	% minorities/majority
4	IGHV1S1*01	IGHJ4*01	ARGGYNYFDY	3	gccagagggggggtacaactactttgactac	9	
4	IGHV1S1*01	IGHJ4*01	ARGGYNYFDY	3	gccaggggggggctacaactactttgactac	2	
4	IGHV1S1*01	IGHJ4*01	ARGGYNYFDY	3	gccagagggggctacaactactttgactac	2	
4	IGHV1S1*01	IGHJ4*01	ARGGYNYFDY	3	all	13	0.307692307692308

	V-Gene	J-Gene	AA	#unique NT	NT	# sequences	% minorities/majority
4	IGHV1S1*01	IGHJ7*01	ARITTAYAAFDS	2	gccaggataactaccgcttacgctgcttttgactcc	6	
4	IGHV1S1*01	IGHJ7*01	ARITTAYAAFDS	2	gccaggataaccaccgcttacgctgcttttgactcc	5	
4	IGHV1S1*01	IGHJ7*01	ARITTAYAAFDS	2	all	11	0.454545454545455

6	V-Gene	J-Gene	AA	#unique NT	NT	# sequences	% minorities/majority
6	IGHV1S1*01	IGHJ5*01	ARAESNNGRHAFDY	2	gccagagcggagtcgaataacgggcgtcatgcttttgactac	6	
6	IGHV1S1*01	IGHJ5*01	ARAESNNGRHAFDY	2	gccaggcggagtcgaataacgggcgtcatgcttttgactac	5	
6	IGHV1S1*01	IGHJ5*01	ARAESNNGRHAFDY	2	all	11	0.454545454545455

	V-Gene	J-Gene	AA	#unique NT	NT	# sequences	% minorities/majority
6	IGHV1S1*01	IGHJ6*01	ARGQNNGRFDY	2	gccagagggcagaataacggccgctttgactac	9	type1
6	IGHV1S1*01	IGHJ6*01	ARGQNNGRFDY	2	gccagagggcagaataacggccgttttgactac	5	type2
6	IGHV1S1*01	IGHJ6*01	ARGQNNGRFDY	2	all	14	0.357142857142857
**This JST has been found in another infected fish (#), encoded by one of the most frequent nucleotide junction in fish# (type 2)**							
4	IGHV1S1*01	IGHJ6*01	ARGQNNGRFDY	1	gccagagggcagaataacggccgttttgactac	6	

VH4-Cmu

	V-Gene	J-Gene	AA	#unique NT	NT	# sequences	% minorities/majority
	IGHV4S1*01	IGHJ7*01	ARERRPAPIELAAFDS	2	gctcgagagcgccggcccgctcccatagagctcgctgcttttgactcc	7	
	IGHV4S1*01	IGHJ7*01	ARERRPAPIELAAFDS	2	gctcgagagcgccggcccgctcccatagagcttgctgcttttgactcc	3	
	IGHV4S1*01	IGHJ7*01	ARERRPAPIELAAFDS	2	all	10	0.3

VH4-Ctau

	V-Gene	J-Gene	AA	#unique NT	NT	# sequences	% minorities/majority
1	IGHV4S1*01	IGHJ1*01	AREFGHMGWGKNAFDY	5	gctcgagagtttggtcatatggggtggggaaagaatgcttttgacta	31	
1	IGHV4S1*01	IGHJ1*01	AREFGHMGWGKNAFDY	5	gctcgagagtttggtcatatggggtgggggaagaatgcttttgacta	3	
1	IGHV4S1*01	IGHJ1*01	AREFGHMGWGKNAFDY	5	gctcgagagtttggtcatatggggtggggaaagaatgcctttgacta	2	

1	IGHV4S1*01	IGHJ1*01	AREFGHMGWGKNAFDY	5	gctcgggagtttggtcatatggggtggggaaagaatgcttttgacta	1	
1	IGHV4S1*01	IGHJ1*01	AREFGHMGWGKNAFDY	5	gcccgagagtttggtcatatggggtggggaaagaatgcttttgacta	1	
1	IGHV4S1*01	IGHJ1*01	AREFGHMGWGKNAFDY	5	all	38	0.184210526315789

4	IGHV4S1*01	IGHJ1*01	ARGYTVTVWAFYAFDY	2	gctcgagggtatacagttacagtttgggctttttatgcttttgactac	1	
4	IGHV4S1*01	IGHJ1*01	ARGYTVTVWAFYAFDY	2	gctcggggatatacagttacagtttgggctttttatgcttttgactac	7	
4	IGHV4S1*01	IGHJ1*01	ARGYTVTVWAFYAFDY	2	all	8	0.125

	V-Gene	J-Gene	AA	#unique NT	NT	# sequences	% minorities/majority
4	IGHV4S1*01	IGHJ1*01	ARGYTVTVWAFYAFDY	5	gctcggggatatacagttacagtttgggctttttatgcttttgactac	10	
4	IGHV4S1*01	IGHJ1*01	ARGYTVTVWAFYAFDY	5	gctcgaggctatacagttacagtttgggctttttatgcttttgactac	7	
4	IGHV4S1*01	IGHJ1*01	ARGYTVTVWAFYAFDY	5	gctcggggatatacagttacagtttgggctttttatgctttcgactac	1	
4	IGHV4S1*01	IGHJ1*01	ARGYTVTVWAFYAFDY	5	gctcgagggtatacagttacagtttgggctttttatgcttttgactac	2	
4	IGHV4S1*01	IGHJ1*01	ARGYTVTVWAFYAFDY	5	gcacggggatatacagttacagtttgggctttttatgcttttgactac	1	
4	IGHV4S1*01	IGHJ1*01	ARGYTVTVWAFYAFDY	5	all	21	0.523809523809524

	V-Gene	J-Gene	AA	#unique NT	NT	# sequences	% minorities/majority
7	IGHV4S1*01	IGHJ1*01	AREGIQLQFGLFIRDAFDY	2	gctcgagaggggatacagttacagtttgggctttttattcgagatgctt	7	
7	IGHV4S1*01	IGHJ1*01	AREGIQLQFGLFIRDAFDY	2	gctcgagagggtatacagttacagtttgggctttttattcgagatgctt	4	
7	IGHV4S1*01	IGHJ1*01	AREGIQLQFGLFIRDAFDY	2	all	11	0.363636363636364

	V-Gene	J-Gene	AA	#unique NT	NT	# sequences	% minorities/majority
7	IGHV4S1*01	IGHJ1*01	ARGYTVTVWTFYAFDY	3	gctcgagggtatacagttacagtttggactttttatgcttttgactac	7	
7	IGHV4S1*01	IGHJ1*01	ARGYTVTVWTFYAFDY	3	gctcgaggctatacagttacagtttggactttttatgcttttgactac	4	
7	IGHV4S1*01	IGHJ1*01	ARGYTVTVWTFYAFDY	3	gctcgaggctatacagttacagtctggactttttatgcttttgactac	2	
7	IGHV4S1*01	IGHJ1*01	ARGYTVTVWTFYAFDY	3	all	13	0.461538461538462

	V-Gene	J-Gene	AA	#unique NT	NT	# sequences	% minorities/majority
7	IGHV4S1*01	IGHJ1*01	ARGYTVTVWVFYAFDY	4	gctcgaggctatacagttacagtttgggtcttttatgcttttgactac	5	type 1
7	IGHV4S1*01	IGHJ1*01	ARGYTVTVWVFYAFDY	4	gctcgagggtatacagttacagtttgggtcttttatgcttttgactac	4	type 2
7	IGHV4S1*01	IGHJ1*01	ARGYTVTVWVFYAFDY	4	gctcgagggtatacagttacagtttgggttttttatgcttttgactac	1	
7	IGHV4S1*01	IGHJ1*01	ARGYTVTVWVFYAFDY	4	gctcgagggtatacagttacggtttgggtcttttatgcttttgactac	1	
7	IGHV4S1*01	IGHJ1*01	ARGYTVTVWVFYAFDY	4	all	11	0.545454545454545
**This JST has been found in another infected fish (#5), encoded by one of the most frequent nucleotide junction in fish#7(type 1)**							
4	IGHV4S1*01	IGHJ1*01	ARGYTVTVWVFYAFDY	1	gctcgaggctatacagttacagtttgggtcttttatgcttttgactac	1	type 1
