# Supplementary material for: Teleost Fish Mount Complex Clonal IgM and IgT Responses in Spleen upon Systemic Viral Infection
Source: PLoS Pathog. 2013 Jan 10;9(1):e1003098. doi: 10.1371/journal.ppat.1003098 (PMC3542120; doi:10.1371/journal.ppat.1003098)

Figure S12. Impact of virus infection on the expression of τ1, τ2, τ3 subtypes, δ isotype and transcription factors blimp1 and Pax5. Relative expression of the constant region of the different Ig isotypes in VHSv infected and in control fish. Results were normalized to EF--1α and β--actin and relative expression was calculated using the Pfaffl method . No amplification of the τ2 subtype was found our clonal fish. Each value is the fold increase as an average of the results of comparison of one infected fish with the results of 3 control fish. Control values are set at 1.
* indicates significant increase with respect to controls (p<0.05).

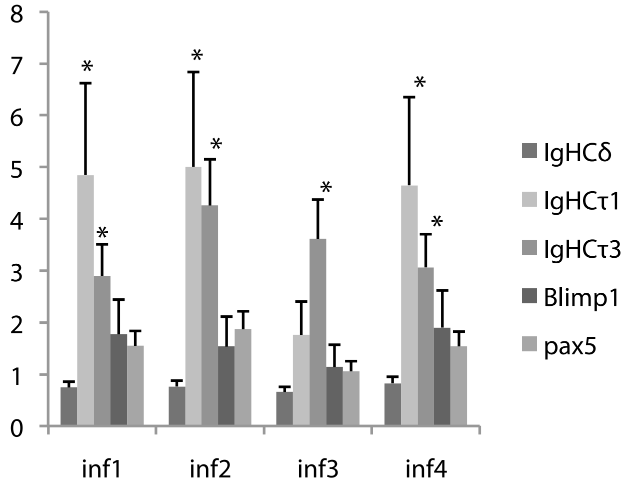

Supplement: Figure S12 — Impact of virus infection on the expression of τ1, τ2, τ3 subtypes, δ isotype and transcription factors blimp1 and Pax5. Relative expression of the constant region of the different Ig isotypes in VHSv infected and in control fish. Results were normalized to EF1α and β-actin and relative expression was calculated using the Pfaffl method. Each value is the fold increase as an average of the results of comparison of one infected fish with the results of 3 control fish. No amplification of the τ2 subtype was found our clonal fish. Control values are set at 1. * indicates significant increase with respect to controls (p<0.05). (PDF) [file ppat.1003098.s012.pdf]
