# Supplementary material for: Teleost Fish Mount Complex Clonal IgM and IgT Responses in Spleen upon Systemic Viral Infection
Source: PLoS Pathog. 2013 Jan 10;9(1):e1003098. doi: 10.1371/journal.ppat.1003098 (PMC3542120; doi:10.1371/journal.ppat.1003098)
Supplement: Figure S13 — Average sequence composition and biochemical properties of VHJμ and VHJτ JST in control and infected fish. VHJμ and VHJτ JST composition and biochemical properties are modified by infection but do not show drastic differences between isotypes. (DOCX) [file ppat.1003098.s013.docx]

**Figure S13. Average sequence composition and biochemical properties of VHJμ and VHJτ JST in control and infected fish**

VHJμ and VHJτ JST composition and biochemical properties are modified by infection but do not show drastic differences between isotypes.

|  | **Isoelectric point** | | | |  |  |  |  |  |  |  |  |  |  |  |  |  |  |  |  |  |  |  |  |  |
| --- | --- | --- | --- | --- | --- | --- | --- | --- | --- | --- | --- | --- | --- | --- | --- | --- | --- | --- | --- | --- | --- | --- | --- | --- | --- |
|  | IGHV1.1 | | | | | | IGHV4 | | | | | | IGHV5.1 | | | | | | IGHV5.4 | | | | | | ctrl |
|  | ctrl | | | inf | | | ctrl | | | inf | | | ± | | | inf | | | ctrl | | | inf | | |  |
| IGHJ3*01 - Chm | 5.71 | ± | 0.20 | 6.08 | ± | 0.49 | 6.3 | ± | 0.0 | 5.4 | ± | 0.6 | 5.68 | ± | 0.45 | 6.12 | ± | 0.47 | 5.26 | ± | 0.43 | 5.49 | ± | 0.28 |  |
| IGHJ4*01 - Chm | 5.70 | ± | 0.08 | 5.62 | ± | 0.37 | 5.9 | ± | 0.1 | 6.0 | ± | 0.8 | 5.59 | ± | 0.18 | 5.67 | ± | 0.26 | 5.48 | ± | 0.38 | 5.35 | ± | 0.44 |  |
| IGHJ5*01 - Chm | 5.70 | ± | 0.22 | 5.73 | ± | 0.19 | 5.9 | ± | 0.3 | 6.8 | ± | 0.8 | 6.01 | ± | 0.06 | 5.22 | ± | 0.31 | 5.60 | ± | 0.25 | 5.22 | ± | 0.08 |  |
| IGHJ6*01 - Chm | 6.96 | ± | 0.28 | 6.84 | ± | 0.37 | 7.2 | ± | 0.1 | 7.2 | ± | 0.6 | 7.25 | ± | 0.11 | 7.60 | ± | 0.64 | 6.35 | ± | 0.14 | 6.87 | ± | 0.50 |  |
| IGHJ7*01 - Chm | 5.78 | ± | 0.07 | 5.52 |  | 0.17 | 5.8 | ± | 0.2 | 5.8 | ± | 0.5 | 6.14 | ± | 0.41 | 5.83 | ± | 0.17 | 4.99 | ± | 0.20 | 5.52 | ± | 0.42 |  |
|  |  |  |  |  |  |  |  |  |  |  |  |  |  |  |  |  |  |  |  |  |  |  |  |  |  |
| IGHJ1*01 - Chtaucom |  |  |  |  |  |  | 5.9 | ± | 0.1 | 5.8 | ± | 0.3 | 6.2 | ± | 0.1 | 6.1 | ± | 0.1 | 5.5 | ± | 0.0 | 5.5 | ± | 0.1 |  |
| IGHJ2*01 - Chtaucom |  |  |  |  |  |  | 6.3 | ± | 0.4 | 5.4 | ± | 0.8 | 6.3 | ± | 0.1 | 6.4 | ± | 0.2 | 5.7 | ± | 0.1 | 5.3 | ± | 0.5 |  |
|  |  |  |  |  |  |  |  |  |  |  |  |  |  |  |  |  |  |  |  |  |  |  |  |  |  |
|  | **Residues negatively charged** | | | | | |  |  |  |  |  |  |  |  |  |  |  |  |  |  |  |  |  |  |  |
|  | IGHV1.1 | | | | | | IGHV4 | | | | | | IGHV5.1 | | | | | | IGHV5.4 | | | | | | ctrl |
|  | ctrl | | | inf | | | ctrl | | | inf | | | ± | | | inf | | | ctrl | | | inf | | |  |
| IGHJ3*01 - Chm | 1.26 | ± | 0.12 | 1.41 | ± | 0.26 | 1.66 | ± | 0.11 | 1.75 | ± | 0.46 | 1.42 | ± | 0.14 | 1.04 | ± | 0.08 | 1.73 | ± | 0.18 | 1.67 | ± | 0.07 |  |
| IGHJ4*01 - Chm | 1.34 | ± | 0.12 | 1.46 | ± | 0.06 | 1.78 | ± | 0.16 | 1.74 | ± | 0.22 | 1.42 | ± | 0.12 | 1.52 | ± | 0.37 | 1.68 | ± | 0.12 | 1.77 | ± | 0.17 |  |
| IGHJ5*01 - Chm | 1.39 | ± | 0.04 | 1.39 | ± | 0.12 | 1.92 | ± | 0.25 | 1.74 | ± | 0.30 | 1.37 | ± | 0.07 | 1.56 | ± | 0.15 | 1.72 | ± | 0.06 | 1.81 | ± | 0.25 |  |
| IGHJ6*01 - Chm | 1.50 | ± | 0.07 | 1.50 | ± | 0.17 | 1.91 | ± | 0.03 | 1.91 | ± | 0.09 | 1.43 | ± | 0.08 | 1.38 | ± | 0.28 | 1.87 | ± | 0.09 | 1.65 | ± | 0.23 |  |
| IGHJ7*01 - Chm | 1.17 | ± | 0.11 | 1.23 |  | 0.13 | 1.90 | ± | 0.18 | 2.06 | ± | 0.53 | 1.06 | ± | 0.08 | 1.00 | ± | 0.26 | 1.77 | ± | 0.17 | 1.64 | ± | 0.36 |  |
|  |  |  |  |  |  |  |  |  |  |  |  |  |  |  |  |  |  |  |  |  |  |  |  |  |  |
| IGHJ1*01 - Chtaucom |  |  |  |  |  |  | 2.04 | ± | 0.09 | 1.80 | ± | 0.29 | 1.25 | ± | 0.06 | 1.26 | ± | 0.08 | 1.84 | ± | 0.05 | 1.70 | ± | 0.23 |  |
| IGHJ2*01 - Chtaucom |  |  |  |  |  |  | 2.12 | ± | 0.08 | 2.13 | ± | 0.22 | 1.31 | ± | 0.04 | 1.27 | ± | 0.06 | 1.75 | ± | 0.04 | 1.82 | ± | 0.06 |  |
|  |  |  |  |  |  |  |  |  |  |  |  |  |  |  |  |  |  |  |  |  |  |  |  |  |  |
|  | **Residues positively charged** | | | | | |  |  |  |  |  |  |  |  |  |  |  |  |  |  |  |  |  |  |  |
|  | IGHV1.1 | | | | | | IGHV4 | | | | | | IGHV5.1 | | | | | | IGHV5.4 | | | | | | ctrl |
|  | ctrl | | | inf | | | ctrl | | | inf | | | ± | | | inf | | | ctrl | | | inf | | |  |
| IGHJ3*01 - Chm | 1.060 | ± | 0.19 | 1.37 | ± | 0.36 | 1.66 | ± | 0.13 | 1.28 | ± | 0.29 | 1.19 | ± | 0.09 | 1.15 | ± | 0.17 | 1.20 | ± | 0.12 | 1.28 | ± | 0.20 |  |
| IGHJ4*01 - Chm | 1.10 | ± | 0.04 | 1.16 | ± | 0.20 | 1.60 | ± | 0.19 | 1.59 | ± | 0.29 | 1.15 | ± | 0.03 | 1.25 | ± | 0.30 | 1.22 | ± | 0.12 | 1.21 | ± | 0.05 |  |
| IGHJ5*01 - Chm | 1.14 | ± | 0.08 | 1.16 | ± | 0.05 | 1.66 | ± | 0.08 | 1.98 | ± | 0.53 | 1.31 | ± | 0.07 | 1.10 | ± | 0.03 | 1.28 | ± | 0.09 | 1.17 | ± | 0.11 |  |
| IGHJ6*01 - Chm | 1.94 | ± | 0.15 | 1.87 | ± | 0.15 | 2.47 | ± | 0.06 | 2.43 | ± | 0.41 | 2.00 | ± | 0.03 | 2.11 | ± | 0.29 | 1.95 | ± | 0.03 | 1.98 | ± | 0.02 |  |
| IGHJ7*01 - Chm | 1.062 | ± | 0.087 | 0.98 |  | 0.05 | 1.58 | ± | 0.27 | 1.81 | ± | 0.32 | 1.13 | ± | 0.18 | 1.00 | ± | 0.07 | 1.06 | ± | 0.06 | 1.28 | ± | 0.23 |  |
|  |  |  |  |  |  |  |  |  |  |  |  |  |  |  |  |  |  |  |  |  |  |  |  |  |  |
| IGHJ1*01 - Chtaucom |  |  |  |  |  |  | 1.76 | ± | 0.07 | 1.47 | ± | 0.26 | 1.36 | ± | 0.06 | 1.31 | ± | 0.02 | 1.40 | ± | 0.04 | 1.29 | ± | 0.10 |  |
| IGHJ2*01 - Chtaucom |  |  |  |  |  |  | 2.11 | ± | 0.04 | 1.61 | ± | 0.46 | 1.41 | ± | 0.04 | 1.47 | ± | 0.08 | 1.41 | ± | 0.01 | 1.21 | ± | 0.38 |  |
|  |  |  |  |  |  |  |  |  |  |  |  |  |  |  |  |  |  |  |  |  |  |  |  |  |  |
|  | **GRAVY** | |  |  |  |  |  |  |  |  |  |  |  |  |  |  |  |  |  |  |  |  |  |  |  |
|  | IGHV1.1 | | | | | | IGHV4 | | | | | | IGHV5.1 | | | | | | IGHV5.4 | | | | | | ctrl |
|  | ctrl | | | inf | | | ctrl | | | inf | | | ± | | | inf | | | ctrl | | | inf | | |  |
| IGHJ3*01 - Chm | -0.37 | ± | 0.029 | -0.36 | ± | 0.0032 | -0.70 | ± | 0.049 | -0.35 | ± | 0.34 | -0.61 | ± | 0.064 | -0.62 | ± | 0.12 | -0.75 | ± | 0.090 | -0.51 | ± | 0.087 |  |
| IGHJ4*01 - Chm | -0.58 | ± | 0.063 | -0.61 | ± | 0.020 | -0.79 | ± | 0.059 | -0.80 | ± | 0.258 | -0.717 | ± | 0.071 | -0.76 | ± | 0.13 | -0.78 | ± | 0.071 | -0.69 | ± | 0.166 |  |
| IGHJ5*01 - Chm | -0.35 | ± | 0.076 | -0.31 | ± | 0.096 | -0.57 | ± | 0.17 | -0.47 | ± | 0.32 | -0.50 | ± | 0.041 | -0.79 | ± | 0.093 | -0.53 | ± | 0.043 | -0.41 | ± | 0.20 |  |
| IGHJ6*01 - Chm | -0.71 | ± | 0.091 | -0.70 | ± | 0.16 | -0.95 | ± | 0.03 | -0.92 | ± | 0.16 | -0.97 | ± | 0.076 | -1.050 | ± | 0.23 | -0.92 | ± | 0.066 | -0.70 | ± | 0.075 |  |
| IGHJ7*01 - Chm | -0.054 | ± | 0.051 | -0.11 |  | 0.080 | -0.38 | ± | 0.06 | -0.28 | ± | 0.42 | -0.25 | ± | 0.019 | -0.25 | ± | 0.036 | -0.17 | ± | 0.059 | -0.32 | ± | 0.088 |  |
|  |  |  |  |  |  |  |  |  |  |  |  |  |  |  |  |  |  |  |  |  |  |  |  |  |  |
| IGHJ1*01 - Chtaucom |  |  |  |  |  |  | -0.26 | ± | 0.0088 | 0.094 | ± | 0.084 | -0.21 | ± | 0.014 | -0.16 | ± | 0.083 | -0.29 | ± | 0.0070 | -0.17 | ± | 0.19 |  |
| IGHJ2*01 - Chtaucom |  |  |  |  |  |  | -0.30 |  | 0.060 | -0.27 | ± | 0.17 | -0.49 | ± | 0.00083 | -0.46 | ± | 0.042 | -0.51 | ± | 0.079 | -0.50 | ± | 0.11 |  |
