# Supplementary material for: Teleost Fish Mount Complex Clonal IgM and IgT Responses in Spleen upon Systemic Viral Infection
Source: PLoS Pathog. 2013 Jan 10;9(1):e1003098. doi: 10.1371/journal.ppat.1003098 (PMC3542120; doi:10.1371/journal.ppat.1003098)
Supplement: Figure S14 — Estimation of the Ab producing cell frequency from clonal expansions of junctions detected in corrected and unprocessed datasets. (A) Estimation of Ab-producing cell (AbPC) frequency. (B) Calculations with unprocessed data. (C) Comparison of frequencies estimated from unprocessed and corrected datasets. (DOCX) [file ppat.1003098.s014.docx]

**Figure S14. Estimation of the Ab producing cell frequency from clonal expansions of junctions detected in corrected and unprocessed** **datasets.**

A. Estimation of Ab-producing cell (AbPC) frequency

The frequency of Ab-producing cells (AbPC) was estimated as follows:

With

n1= number of transcripts from resting cells in the sample

n2= number of transcripts from AbPC in the sample

N1= number of resting cells in the sample

N2= number of AbPC in the sample

One considered that one AbPC contains 1000 times more transcripts than a resting B cell,

If n1 transcripts are produced by N1 resting cells

then n2 transcripts are produced by N2 cells with:


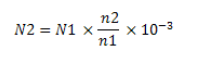


The total number of cells in the samples will be:


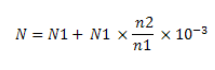


Hence the proportion of resting cells is:


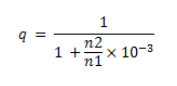


And the proportion of AbPC is:


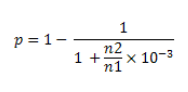


n1 was estimated by the number of sequence reads corresponding to sequence types present less than 5 times

n2 was estimated by the number of sequence reads corresponding to sequence types present 5 times or more

The threshold 5 is well below the number of reads per JST for which the one-sided p values - associated to {H0= This JST is expressed by an activated clone (ie an Ab PC)} as calculated in the model presented Figure S9 - are significant. On the other hand, in the same model {H0= This JST is expressed by a resting clone} is rejected for any value >0, ie as soon as a sequence is found once or more! We choose the threshold 5 here to take into account the fact that in pyrosequencing, the emulsion-based PCR from individual molecules makes possible that a sequence may appear a few times instead of only one, making clonality comparisons comparative rather than absolute [38].

Additionally, the calculation was performed in parallel with the observed unprocessed data, and with the corrected dataset to take into account the experimental sequencing error. Both sets of results are presented below and lead to similar conclusions.

The estimation of n1 and n2 depend on the sequencing depth since the number of sequence types present 5 times of more is growing with the total number of sequence reads. We therefore computed here a *minimal* estimation of Ab producing cells.

Importantly, to compare the proportions of AbPC in different datasets, for example between control and infected fish and for IgM or IgT with a given VH , it was necessary to compute the estimations from datasets of equivalent size (i. e. similar numbers of sequence reads).

To do so, the total number of sequences was computed for the situations to compare ( i. e. control or infected , μ or τ, , for a given VH ). We then considered the minimum number (MIN) of sequence reads among these four situations.

We constituted for each of the other three situations a randomly chosen subset composed of MIN sequences, to simulate a dataset with the same depth for all the situations. Then we counted for each situation the number of sequence reads corresponding to sequence types present less than 5 times in each of these simulated datasets (hereafter called n1mins and n2mins).

We iterated this process 1000 times and computed the mean and the standard deviation of nmins for each situation. In fact, the process converges rapidly and a 1000-time repeat constituted a very robust number of trials.

These mean values of the n1mins and n2mins provided the estimations of the number of sequence reads corresponding to sequence types present less than 5 times and 5 times or more, respectively.

**B) Calculations with unprocessed data**

Simulated dataset and frequency calculations:

| 454 dataset | Threshold | Average | Standard deviation | Nb seq< T | NbSeq> T | Freq of resting Cells | Freq of Ab Prod. cells | |
| --- | --- | --- | --- | --- | --- | --- | --- | --- |
| VH4_Chm_control | 5 | 780 | 13.67 | 780 | 415,00 | 9,99E-01 | | 5,32E-04 |
| VH4_Chm_infected | 5 | 439 | 14.56 | 439 | 756,00 | 9,98E-01 | | 1,72E-03 |
| VH4_Chtaucom_control | 5 | 1190 | 0,00 | 1190 | 5,00 | 1,00E+00 | | 4,20E-06 |
| VH4_Chtaucom_infected | 5 | 404 | 13.32 | 404 | 791,00 | 9,98E-01 | | 1,95E-03 |
|  |  |  |  |  |  |  | |  |
| VH51_Chm_control | 5 | 1378 | 14.63 | 1368 | 587 | 1,00E+00 | | 4,29E-04 |
| VH51_Chm_infected | 5 | 373 | 13.83 | 373 | 1592 | 9,96E-01 | | 4,25E-03 |
| VH51_Chtaucom_control | 5 | 1823 | 0,00 | 1823 | 142 | 1,00E+00 | | 7,79E-05 |
| VH51_Chtaucom_infected | 5 | 1497 | 13.40 | 1497 | 468 | 1,00E+00 | | 3,13E-04 |
|  |  |  |  |  |  |  | |  |
| VH54_Chm_control | 5 | 1944 | 0,00 | 1944 | 994 | 9,99E-01 | | 5,11E-04 |
| VH54_Chm_infected | 5 | 1203 | 19.73 | 1203 | 1735 | 9,99E-01 | | 1,44E-03 |
| VH54_Chtaucom_control | 5 | 2646 | 6.13 | 2646 | 292 | 1,00E+00 | | 1,10E-04 |
| VH54_Chtaucom_infected | 5 | 2082 | 17.32 | 2082 | 856 | 1,00E+00 | 4,11E-04 | |

^1^Average of the number of sequences corresponding to sequence types present less than 5 times. The number corresponding to the configuration with the min value if total read numbers among the set to be compared is in bold.

^2^ Nb seq < (respectively >)T : Nb of sequence reads corresponding to sequence types present less (respectively more than 5 times)

**C) Comparison of frequencies estimated from unprocessed and corrected datasets**

|  |  | **Unprocessed** | **Unprocessed** |  | **Corrected** | **Corrected** |  |
| --- | --- | --- | --- | --- | --- | --- | --- |
| 454 dataset | Threshold | Freq of resting Cells | Freq of Ab Prod. cells |  | Freq of resting Cells | Freq of Ab Prod. cells |  |
| VH4_Chm_control | 5 | 9,99E-01 | 5,32E-04 |  | 9,99E-01 | 6,28E-04 |  |
| VH4_Chm_infected | 5 | 9,98E-01 | 1,72E-03 |  | 9,97E-01 | 3,35E-03 |  |
| VH4_Chtaucom_control | 5 | 1,00E+00 | 4,20E-06 |  | 1,00E+00 | 1,61E-05 |  |
| VH4_Chtaucom_infected | 5 | 9,98E-01 | 1,95E-03 |  | 9,95E-01 | 4,91E-03 |  |
|  |  |  |  |  |  |  |  |
| VH51_Chm_control | 5 | 1,00E+00 | 4,29E-04 |  | 1,00E+00 | 4,91E-04 |  |
| VH51_Chm_infected | 5 | 9,96E-01 | 4,25E-03 |  | 9,93E-01 | 6,67E-03 |  |
| VH51_Chtaucom_control | 5 | 1,00E+00 | 7,79E-05 |  | 1,00E+00 | 9,20E-05 |  |
| VH51_Chtaucom_infected | 5 | 1,00E+00 | 3,13E-04 |  | 1,00E+00 | 4,88E-04 |  |
|  |  |  |  |  |  |  |  |
| VH54_Chm_control | 5 | 9,99E-01 | 5,11E-04 |  | 9,99E-01 | 5,84E-04 |  |
| VH54_Chm_infected | 5 | 9,99E-01 | 1,44E-03 |  | 9,98E-01 | 1,74E-03 |  |
| VH54_Chtaucom_control | 5 | 1,00E+00 | 1,10E-04 |  | 1,00E+00 | 1,25E-04 |  |
| VH54_Chtaucom_infected | 5 | 1,00E+00 | 4,11E-04 |  | 9,99E-01 | 5,86E-04 |  |
